# Dye-Sensitized Solar Cells Based on Cu(I) Complexes Containing Catechol Anchor Groups That Operate with Aqueous Electrolytes

**DOI:** 10.1021/jacsau.5c00601

**Published:** 2025-07-29

**Authors:** Lars E. Burmeister, Florian Doettinger, Kurt J. Haseloff, Christian Kleeberg, Mohammed Boujtita, Simon Pascal, Fabrice Odobel, Stefanie Tschierlei, Yann Pellegrin, Michael Karnahl

**Affiliations:** † Department of Energy Conversion, Institute of Physical and Theoretical Chemistry, Technische Universität Braunschweig, Rebenring 31, 38106 Braunschweig, Germany; ‡ Institute of Inorganic and Analytical Chemistry, Technische Universität Braunschweig, Hagenring 30, 38106 Braunschweig, Germany; § Nantes Université, CNRS, CEISAM, UMR6230, F-44000 Nantes, France

**Keywords:** Cu(I) photosensitizers, catechol anchor groups, dye-sensitized solar cells (DSSCs), type-II sensitization, aqueous electrolytes

## Abstract

We report the design and synthesis of two novel phenanthroline-based ligands, a catechol-functionalized derivative (**L1**: 4,7-(4-catechol)-2,9-dimethyl-1,10-phenanthroline) ligand and its methoxy analogue (**L1′**). These ligands were used to prepare four Cu­(I) complexes: two homoleptic bis-diimine Cu­(I) complexes (**C1** and **C1′**) and two xantphos-based heteroleptic diimine-diphosphine derivatives (**C2** and **C2′**). Their photophysical and electrochemical properties were characterized by steady-state and time-resolved spectroscopy, cyclic voltammetry, and density functional theory (DFT) calculations. Implementation of **L1**, **C1**, and **C2** in n-type DSSCs gave a photoconversion efficiency (PCE) of 1.88% for the heteroleptic Cu­(I) complex **C2**, representing a 35-fold increase compared to previously reported diimine-diphosphine Cu­(I)-based DSSCs. Incident photon-to-current efficiency (IPCE) measurements and electrochemical impedance spectroscopy confirmed efficient photoinduced charge injection and interfacial electron transfer. Moreover, the strong binding affinity of the catechol anchors enabled the fabrication of DSSCs with an aqueous electrolyte, which showed stable performance for at least 10 days. The working principle of these cells is described as a dual chromophore system, where the catechol-TiO_2_ interaction, operating through a type-II sensitization mechanism, acts as the primary chromophore, while the Cu­(I) complex serves as an antenna chromophore.

## Graphical abstract

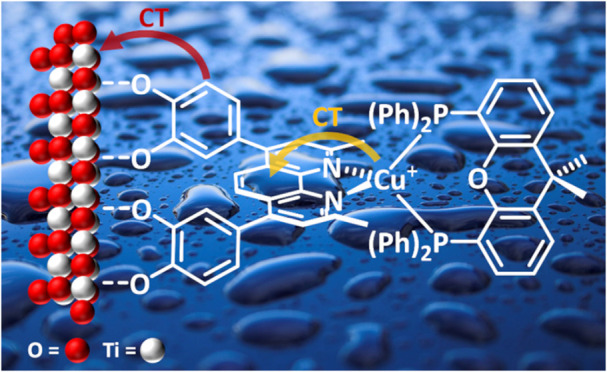

## Introduction

Since the pioneering work by Grätzel and O’Regan in 1991,^1^ dye-sensitized solar cells (DSSCs) have emerged as a promising alternative to silicon-based photovoltaics.^2,3^ With optimized molecular dyes and device architectures, photoconversion efficiencies (PCEs) of up to 15% have been achieved in recent years.^4^ Among the various classes of sensitizers, Cu­(I)-based complexes bearing two diimine ligands have been extensively studied in photocatalysis^5–9^ or DSSCs.^9–12^ They have been established as a promising alternative to complexes based on rare and expensive noble metals such as Ru­(II) or Ir­(III), as Cu is more abundant in Earth’s crust.^3,13–17^ Further research was then directed toward the substitution of one diimine ligand by a diphosphine ligand to form heteroleptic Cu­(I) complexes of the type [Cu­(NN)­(PP)]^+^, as they provide some advantageous photophysical properties, *i.e.*, higher quantum yields, longer emission lifetimes and higher excited-state energies.^7,18–21^ In spite of these superior properties, this type of complexes has hardly been used as photosensitizers in DSSCs. To the best of our knowledge, only one reference by Robertson and co-workers reported the use of [Cu­(dcbpy)­(DPEPhos)]^+^ (dcbpy = 4,4′-dicarboxy-2,2′-bipyridine, DPEPhos = bis­[(2-diphenylphosphino) phenyl]­ether) to sensitize TiO_2_ and the implementation of the resulting photoanode in a DSSC (*cf*. Figure S14.11 and Table S14.6).^22^ Despite of significant thermodynamic driving force for electron injection and dye regeneration, only a very low photoconversion efficiency (PCE) was obtained (0.053%). This was caused by the low light-harvesting efficiency (LHE) of the photoanode.^22^ In fact, the absorption maxima of common heteroleptic Cu­(I) complexes are usually in the bluest part of the visible spectrum.^7,20,21^ Significant efforts have been made to shift the absorption further into the visible, by tuning the electronic properties of the diimine ligand with different electron-donating or electron-accepting groups^23–26^ or by extending the π-system.^27–30^ However, to the best of our knowledge, DSSCs with such chemically engineered complexes have never been realized so far.

In this contribution, a promising strategy to improve the LHE of the photoanode by using catechol substituents (1,2-dihydroxybenzene) as anchor groups is presented.^31–33^ Catechol-type anchors are rarer than carboxylic or phosphonic acids anchors, though they exhibit two important advantages: First, the interaction between catechols and TiO_2_ results in a new absorption band spreading in the visible (called “type-II sensitization”),^34–38^ which improves the light-harvesting efficiency. Second, catechol groups are tightly bound to TiO_2_, resulting in very stable dye monolayers.^31,37–42^ To date, no examples of heteroleptic [Cu­(NN)­(PP)]^+^ complexes featuring catechol anchor groups have been implemented in DSSCs.

Therefore, this contribution reports our efforts to combine the excellent photophysical properties of heteroleptic Cu­(I) complexes with the advantageous features of catechol anchors. For the first time, we have functionalized the widely studied 2,9-dimethyl-1,10-phenanthroline ligand (dmp)^43,44^ with two catechol substituents at the 4,7-positions (**L1**). In addition, the corresponding homoleptic Cu­(I) complex [Cu­(**L1**)_2_]^+^ **C1** and the heteroleptic counterpart [Cu­(**L1**)­(xantphos)]^+^ **C2** (xantphos = (9,9-dimethyl-9*H*-xanthene-4,5-diyl)­bis­(diphenylphosphane), Figure 1) were prepared. The presence of methyl groups at the 2,9-positions is crucial, as it confers significantly longer lifetimes to the resulting Cu­(I) complexes by reducing exciplex quenching.^20,45,46^

**Figure 1:**
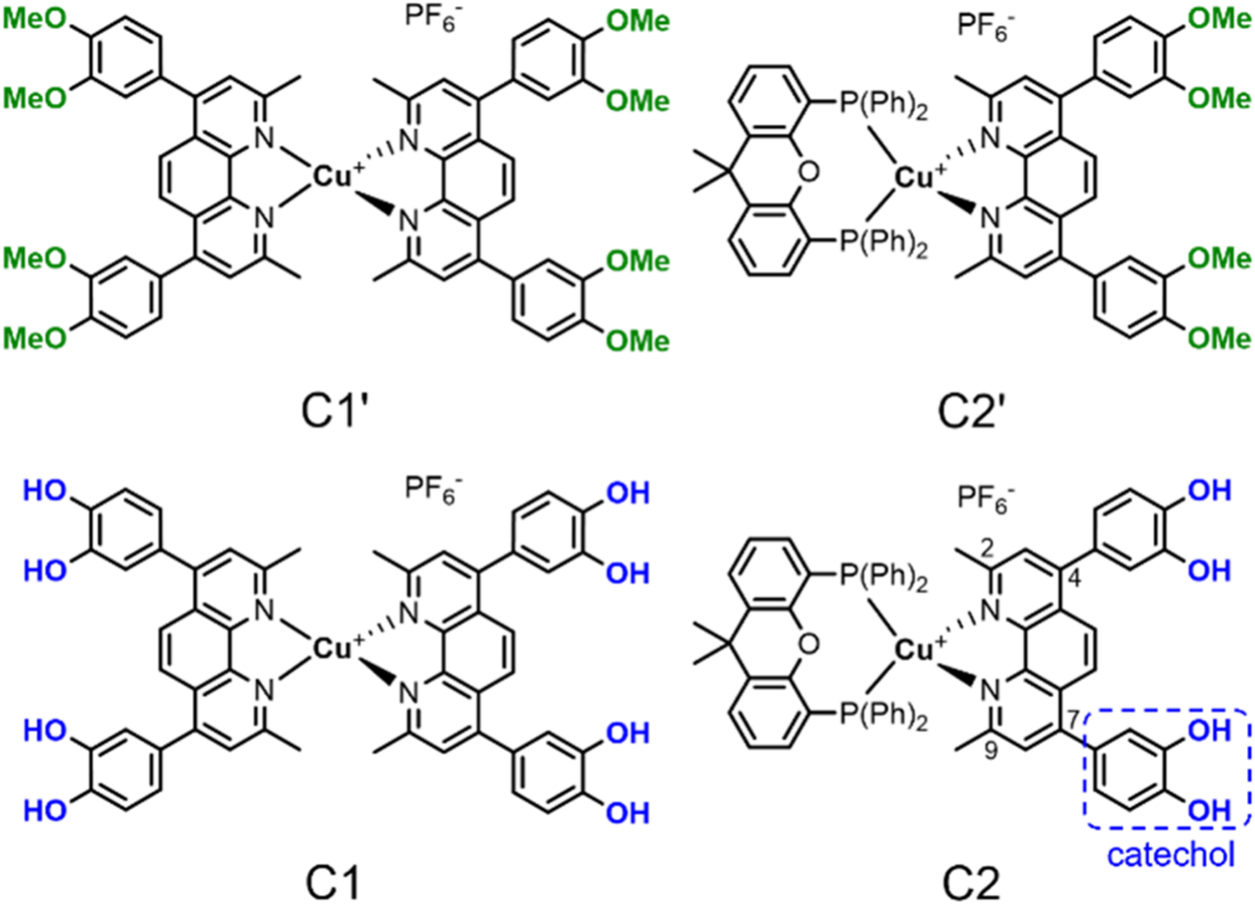
Molecular structures of the novel homoleptic bisdiimine Cu­(I) complexes **C1′** and **C1** (left) and their heteroleptic diimine-diphosphine counterparts **C2′** and **C2** (right) containing the xantphos ligand. The different positions of the substituents at the phenanthroline ligand are indicated by 2, 4, 7, and 9 (bottom right).

To obtain a comprehensive picture, the photophysical and electrochemical properties of the novel dyes as well as of the 3,4-dimethoxyphenyl-substituted precursors **L1′**, **C1′**, and **C2′** were investigated in detail. This included steady-state and time-resolved absorption/emission spectroscopy, cyclic voltammetry and density functional theory (DFT) calculations to gain deeper insights into their electronic structures and excited-state dynamics. Finally, the three novel catechol dyes were implemented in n-type DSSCs, and their performance was systematically studied in both organic and aqueous electrolytes, with a focus on key photovoltaic parameters and device stability. By combining synthetic, spectroscopic, electrochemical and theoretical approaches, this work provides a comprehensive foundation for the further advancement of Cu­(I)-based sensitizers in DSSCs.

## Results and Discussion

### Synthesis

The catechol-functionalized phenanthroline ligand **L1** was synthesized starting from 4,7-dichloro-2,9-dimethyl-1,10-phenanthroline^47^ via a Suzuki–Miyaura cross-coupling with (3,4-dimethoxyphenyl)­boronic acid (Scheme 1).^48^ This reaction was carried out in a basic mixture of tetrahydrofuran and water utilizing a XPhos-Pd-G2 precatalyst (see Supporting Information (SI)–Chapter 2 for further details).^49^ The required substrate 4,7-dichloro-2,9-dimethyl-1,10-phenanthroline and the XPhos-Pd-G2 precatalyst were first synthesized according to literature known methods.^47,49^ In a next step, the obtained 4,7-bis­(3,4-dimethoxyphenyl)-2,9-dimethyl-1,10-phenanthroline (**L1′**) was deprotected with pyridinium chloride, adapting a procedure described in the literature.^50^ The desired 4,4′-(2,9-dimethyl-1,10-phenanthroline-4,7-diyl)­bis­(benzene-1,2-diol) (**L1**) (Scheme 1) was then received as a red colored solid in a total yield of 60%.

**Scheme 1:**
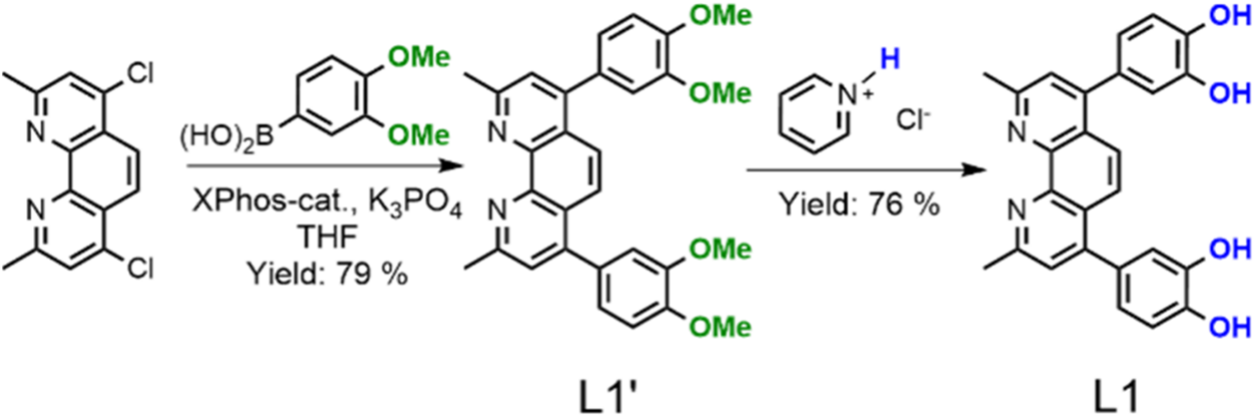
Presentation of the Synthetic Pathway Leading to **L1′** and **L1**, Including the Main Reaction Conditions and Yields

The four corresponding Cu­(I) complexes (Figure 1) were synthesized according to well-established literature procedures.^26,51–53^ The homoleptic complex **C1′** containing the methoxy protecting groups was prepared by coordinating **L1′** to the Cu­(I) precursor [Cu­(MeCN)_4_]­PF_6_ (MeCN = acetonitrile) in dichloromethane (DCM) at room temperature. In contrast, the homoleptic catechol complex **C1** was synthesized by the coordination of **L1** to the Cu­(I) precursor using methanol (MeOH) as a solvent, due to the low solubility of **L1** in less polar solvents.

The heteroleptic complexes **C2′** and **C2** were prepared via a two-step one-pot procedure, where the xantphos ligand is first coordinated to the Cu­(I) precursor, followed by the careful addition of the substituted dmp ligand in DCM at low temperature.^20,26,54^ To avoid the formation of the homoleptic byproduct, an automatic syringe pump was used for the slow (8–12 mL/h) and precise addition of the diimine ligand. For the preparation of **C2**, MeOH was added to the DCM solution to improve **L1**’s solubility. The catechol containing complexes **C1** and **C2** were purified by size exclusion chromatography (Sephadex LH-20, MeOH/DCM (1/1)) yielding 74% of **C1** and 59% of **C2**. Complete synthetic procedures are given in the SI–Chapter 2.

### Structural Characterization

The identity of the dmp-based ligands and their respective homoleptic and heteroleptic Cu­(I) complexes was verified by nuclear magnetic resonance spectroscopy (NMR: ^1^H, ^13^C­{^1^H}, and ^31^P­{^1^H} where appropriate) and electrospray ionization high resolution mass spectrometry (ESI-HRMS). In addition, single crystals of **C2′** could be obtained as the solvate **C2′**(CH_2_Cl_2_)_2_ by layering a concentrated dichloromethane solution with *n*-heptane. The growth of single crystals of sufficient quality for X-ray analysis was completed after 4 days. Unfortunately, crystallization of the remaining complexes could not be achieved despite numerous attempts. In addition, DFT calculations (B3LYP-D3/def2-TZVP, CPCM­(MeCN)) were conducted to investigate possible structural differences between the compounds and the impact of the catechol anchor groups. To ensure the reliability of the computed structures, the DFT-predicted structure of **C2′** was compared with its experimentally determined solid-state structure (*cf*. Figure 2 and Table 1).

**Figure 2:**
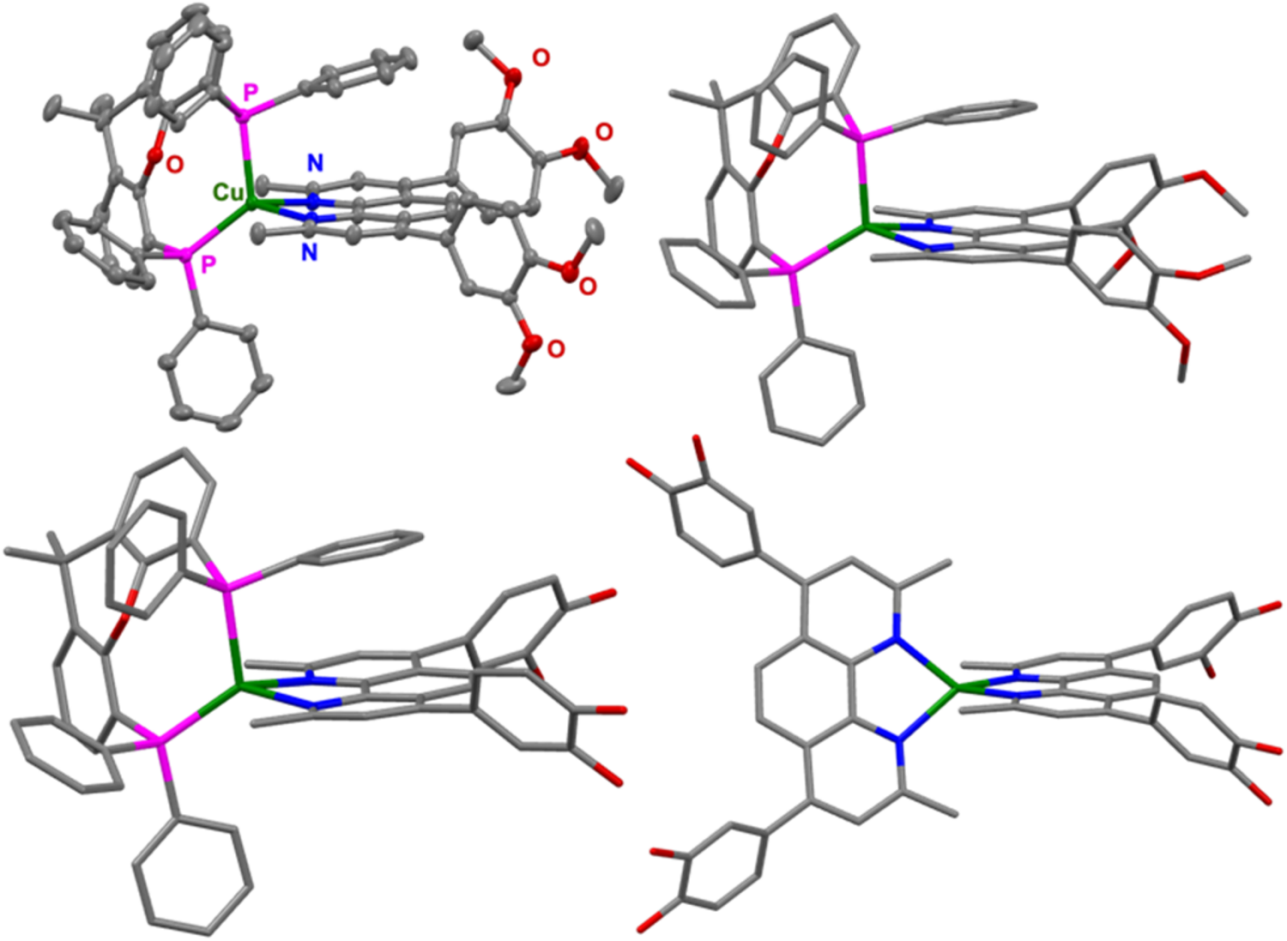
Solid-state structure of **C2′** obtained from single crystal X-ray diffraction analysis (top left) and its optimized geometry predicted by DFT calculations (B3LYP-D3/def2-TZVP, CPCM­(MeCN)) (top right). Optimized geometries of **C2** (bottom left) and **C1** (bottom right) are depicted below for comparison.

**Table 1:** Selected Bond Lengths (pm), Bite Angles (deg), Interplane Angles between the N–Cu–N/P–Cu–P Planes (NN–Cu–NN or PP–Cu–NN, deg) and Torsion Angles τ_sub_ (deg) of the Structure of **C2′** Obtained from X-ray Diffraction Studies and of the Structures of **C1**–**C2′** Predicted by DFT Calculations

	**C1**	**C2**	**C1′**	**C2′**	
	calc.	calc.	calc.	calc.	exp.
Cu–N	205.7	212.0	204.8	212.1	208.5(10)
	205.8	213.2	205.6	213.2	209.0(10)
	206.0		205.7		
	206.0		206.4		
Cu–P		226.6		226.6	224.5(3)
		230.8		230.7	230.6(4)
C–O_para_	136.1	136.2	137.2	137.2	136.3(16)/136.5(15)
C–O_meta_	136.7	136.7	135.6	135.6	135.7(16)/136.0(16)
N–Cu–N	81.0	78.8	81.1	78.9	80.4(4)
	81.1		81.1		
P–Cu–P		119.0		119.0	122.6(13)
NN–Cu–NN	79.9		84.8		
PP–Cu–NN		85.2		85.3	87.7
τ_sub,1_	47.9	52.4	51.4	56.0	55.5(18)
τ_sub,2_	49.5	54.7	51.5	58.2	91.8(17)
τ_sub,3_	50.5		52.5		
τ_sub,4_	51.4		53.9		

Comparison of the calculated Cu–N and Cu–P bond lengths as well as of the N–Cu–N and P–Cu–P bite angles (*cf*. Table 1) reveal a good agreement with the values obtained from X-ray structure determination for **C2′**. This supports the suitability of the method used for the DFT calculations and ensures reasonable predictions. The complexes show a distorted tetrahedral geometry around the copper center, indicated by a small N–Cu–N bite angle (*ca*. 80°) and a wide P–Cu–P bite angle (*ca*. 120°), which is common for these types of compounds.^26,52,55^ In the heteroleptic complexes the Cu–P bond length is significantly longer than Cu–N, *e.g*. about 230 pm *vs*. 209 pm for **C2′**. The two homoleptic complexes **C1** and **C1′** show a somewhat more flattened geometry, indicated by slightly smaller interplane angles (Figure S5.1). The substituents in the 4,7-position of the 1,10-phenanthroline moiety possess torsion angles τ_sub_ of around 50° toward the dmp-plane, implying a low degree of π-conjugation.^54,55^ The torsion angle of 91.8° of one 3,4-dimethoxyphenyl substituent found in the crystal structure of **C2′** is assumed to originate from crystal packing effects. A comparison of the C–O bond lengths reveals comparable values, indicating that the presence of methoxy or hydroxy groups does not exert a significant structural influence neither in the homoleptic nor the heteroleptic complexes.

### Photophysical Properties of the Dyes in Solution

To elucidate their capabilities as sensitizers in DSSCs, the three catechol dyes (**L1**, **C1**, and **C2**) and their methoxy protected derivatives (**L1′**, **C1′**, and **C2′**) were first investigated regarding their photophysical properties in solution (see Table 2). Due to the low solubility of the catechol derivatives in MeCN, which is required for comparison with other Cu­(I) complexes,^21,26,55^ the photophysical characterization was additionally conducted in MeOH.

**Table 2:** Summary of the Photophysical Properties of the Investigated Dyes in Acetonitrile (MeCN) and Methanol (MeOH) Solutions^a^

	λ_abs_/nm (ε/10^3^ M^–1^ cm^–1^)		λ_em_/nm (ϕ_em_/%)		τ_em_/μs^e^	
	MeCN	MeOH	MeCN	MeOH	MeCN	MeOH
**L1′**	280	280	394^b^	411^b^	≤0.01^f^	≤0.01^f^
	(38.1)	(29.5)	(14.0)	(0.5)		
**L1**	not soluble	283	not soluble	not emissive^b^	not soluble	not emissive
		(26.2)				
**C1′**	478	477	not emissive^c^	not emissive^c^	not emissive	not emissive
	(11.4)	(15.3)				
**C1**	not soluble	476	not soluble	not emissive^c^	not soluble	not emissive
		(14.6)				
**C2′** ^h^	400	400	573^d^	571^d^	0.84^g^	14.37^g^
	(6.1)	(7.4)	(2.4)	(38.1)		
**C2** ^h^	400	400	569^d^	565^d^	0.90^g^	6.50^g^
	(5.2)	(9.0)	(1.8)	(2.9)		

a All data were determined under inert conditions at room temperature, apart from the absorption.

b Excited at λ = 310 nm.

c Excited at λ = 470 nm.

d Excited at λ = 400 nm.

e Excited at λ = 355 nm.

f Detected at λ = 400 nm.

g Detected at λ = 570 nm.

h Due to a steady decrease of the absorption, no maximum can be determined.

The ligands **L1′** and **L1** exhibit two intense UV absorption bands, with a prominent higher-energy band around 280 nm and a lower-energy band near 325 nm (see Figure 3 and SI–Chapter 8). The lower-energy band is primarily attributed to pure intraligand charge transfer transitions from the catechol (**L1**) or the 3,4-dimethoxyphenyl substituent (**L1′**) to the phenanthroline moiety, while the higher-energy band also involves mixed π–π* transitions localized on the phenanthroline core (ligand centered, see SI–Chapter 7). Both transitions are solvent independent and are also present in the corresponding Cu­(I) dyes. The introduction of a Cu­(I) center induces an additional absorption band in the visible range due to a metal-to-ligand charge transfer (MLCT) transition, which is well-known for such Cu­(I) compounds.^20,21,45,56^ Compared to the heteroleptic complexes **C2′** and **C2**, the homoleptic complexes **C1′** and **C1** show a red shift of the MLCT transition band of about 100 nm, while the attenuation coefficients are roughly two times higher (*e.g.* ε = 11.4 × 10^3^ M^–1^ cm^–1^ and 6.1 × 10^3^ M^–1^ cm^–1^ for **C1′** and **C2′** in MeCN, *cf*. Table 2). All observations are consistent with the transitions predicted by TDDFT (see SI–Chapter 7). Therefore, the homoleptic complex appears to be the more efficient light harvester for application in DSSCs.

**Figure 3:**
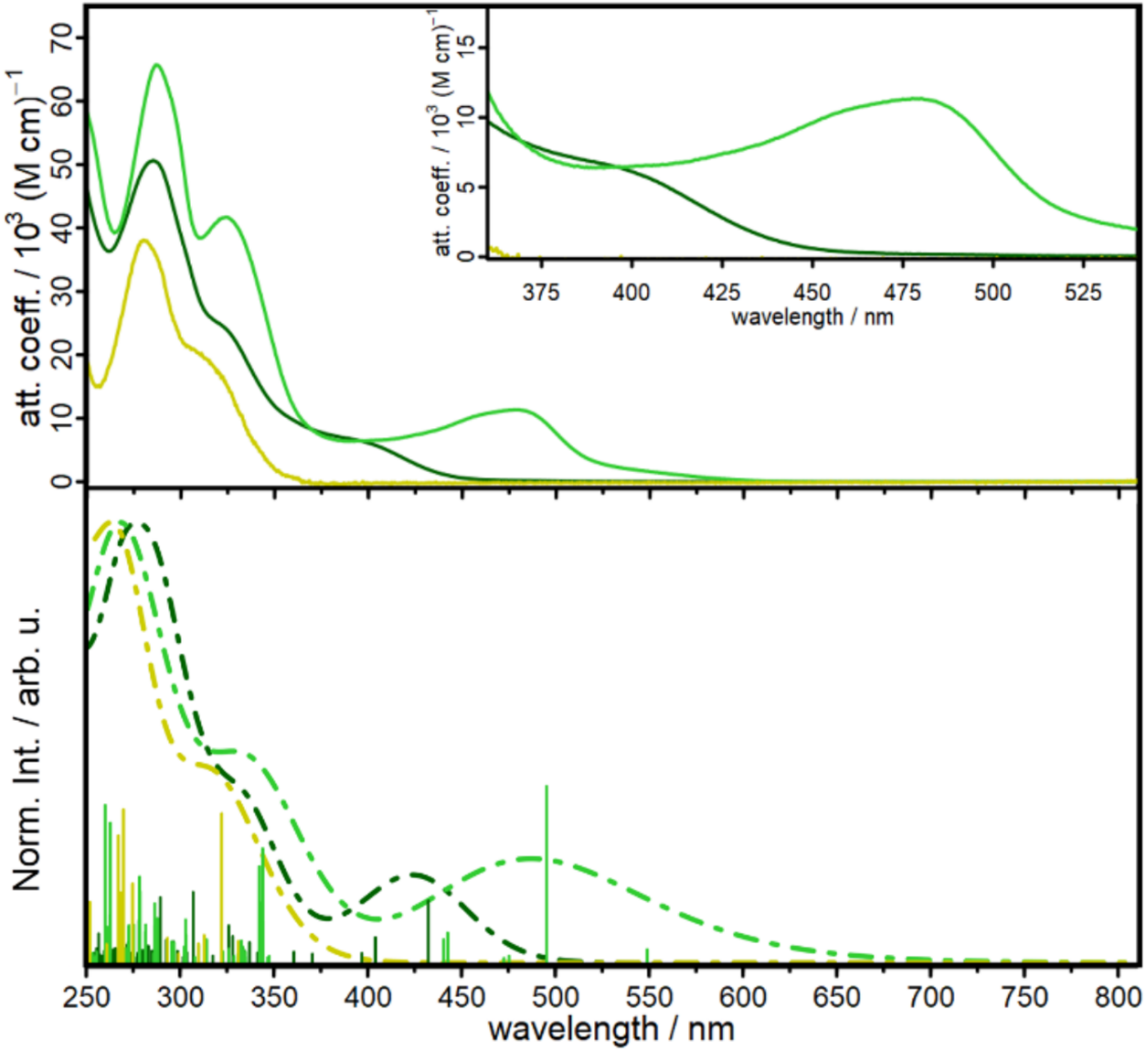
UV/vis absorption spectra of the dyes **L1′** (yellow), **C1′** (green) and **C2′** (dark green) in acetonitrile solution (top) and respective calculated TDDFT spectra (bottom, B3LYP-D3/def2-TZVP, CPCM­(MeCN)).

To elucidate the photostability of the complexes in solution, changes in the UV/vis absorption spectra of **C1′** and **C2′** were recorded in MeCN while irradiated with visible light over a period of 24 h (see SI–Chapter 13 for further details). Only minor spectral changes for **C1′** and **C2′** could be observed, confirming high stability of the Cu­(I) complexes in solution.

Excitation of **L1′** (λ_exc_ = 310 nm) results in an emission at around 400 nm, with a significantly higher quantum yield in MeCN compared to MeOH (ϕ_em,MeCN_ = 14.0% and ϕ_em,MeOH_ = 0.5%, *cf*. Table 2). The lifetime of the emissive excited state was determined to be shorter than 10 ns (*cf*. Figure S10.1), indicating emission from a singlet state.^57,58^ In contrast to **L1′**, no emission was detected for **L1**.

Similarly, the homoleptic complexes **C1′** and **C1** exhibit no emission in either MeCN or MeOH (Table 2), which is consistent with exclusive relaxation through nonradiative decay. This behavior is well-known for homoleptic bisdiimine Cu­(I) complexes, where efficient excited-state deactivation prevents radiative decay.^45,46,59–61^

Bulky diphosphine ligands are an extensively studied tool to increase the radiative decay rate constants.^19–21^ In accordance with this, the heteroleptic complexes **C2′** and **C2** show a broad emission around 570 nm, which commonly originates from the ^3^MLCT state (see Table 2 and Figure 4).^6,20,21^

**Figure 4:**
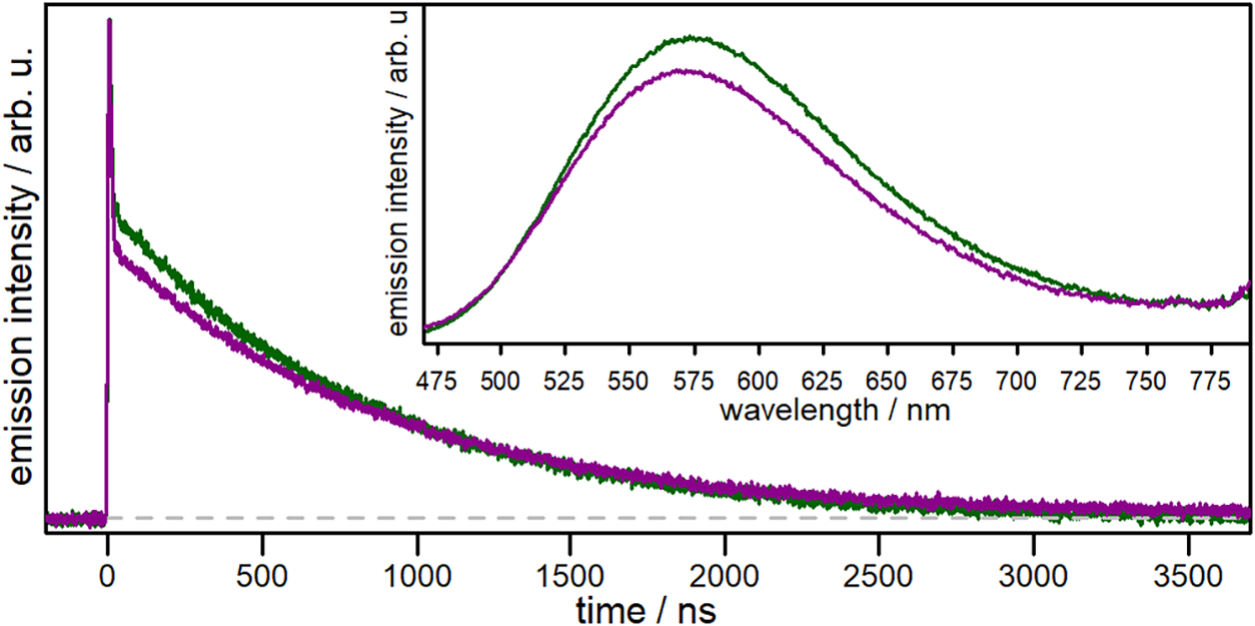
Emission decay curves of **C2′** (dark green) and **C2** (magenta) upon excitation at λ = 355 nm in inert MeCN. The inset shows the respective steady-state emission spectra.

Among all investigated complexes, the highest quantum yield was determined for **C2′** (ϕ_em,MeOH_ = 38.1%) in MeOH. Changing the solvent from MeOH to MeCN significantly decreases the emission quantum yield (ϕ_em,MeCN_ = 2.4% for **C2′**, *cf*. Table 2). This can be explained by the more coordinating nature of MeCN, which is known to promote the formation of an exciplex by nucleophilic attack, that quenches the excited state.^7,45,46,62^ Furthermore, for the complexes **C2′** and **C2** it was found, that the substitution of the methoxy by a hydroxy group decreases the quantum yield significantly in MeOH (ϕ_em,MeOH_ = 38.1% *vs*. 2.9%) and moderately in MeCN (ϕ_em,MeCN_ = 2.4% *vs*. 1.8%). Nevertheless, the introduction of both, methoxy and hydroxy groups increases the emission quantum yields of these complexes. For instance, the quantum yield of the literature known reference complex [Cu­(bcp)­(xant)]­PF_6_ (**CRef**, with bcp = 2,9-dimethyl-4,7-diphenyl-1,10-phenanthroline) is about 2-fold lower (ϕ_em,MeCN_ = 1.35%)^7^ compared to **C2′** in MeCN.

A clear trend is also observed for the emission lifetimes of **C2** (τ_em,MeCN_ = 0.90 μs, τ_em,MeOH_ = 6.50 μs) and **C2′** (τ_em,MeCN_ = 0.84 μs, τ_em,MeOH_ = 14.37 μs, Table 2). The introduction of methoxy groups in **C2′** leads to a 3-fold increase of the excited state lifetime compared to **CRef** (τ_em,MeCN_ = 0.31 μs^7^ and τ_em,MeOH_ = 4.66 μs, Figure S10.2). However, replacing the methoxy by hydroxy groups (**C2′** *vs*. **C2**) has only a minor impact on the emission lifetimes of the heteroleptic complexes in both solvents.

These findings emphasize that the incorporation of electron-donating phenyl-methoxy substituents at the 4,7-positions of the phenanthroline moiety leads to higher quantum yields and longer emission lifetimes, as previously demonstrated in the literature.^26,55^ Nevertheless, the novel heteroleptic Cu­(I)-based dyes exhibit beneficial photophysical properties, highlighting **C2** a promising sensitizer for DSSCs.

### Electrochemical Properties of the Dyes in Solution

The methoxy containing dyes **L1′**, **C1′**, and **C2′** were further studied concerning their electrochemical properties by differential pulse voltammetry and cyclic voltammetry in MeCN solution (see Table 3 and Figure 5). For the compounds with catechol anchor groups **L1**, **C1** and **C2** it was not possible to obtain suitable experimental results due to problems with solubility and aggregation on the electrode surface.^11^

**Figure 5:**
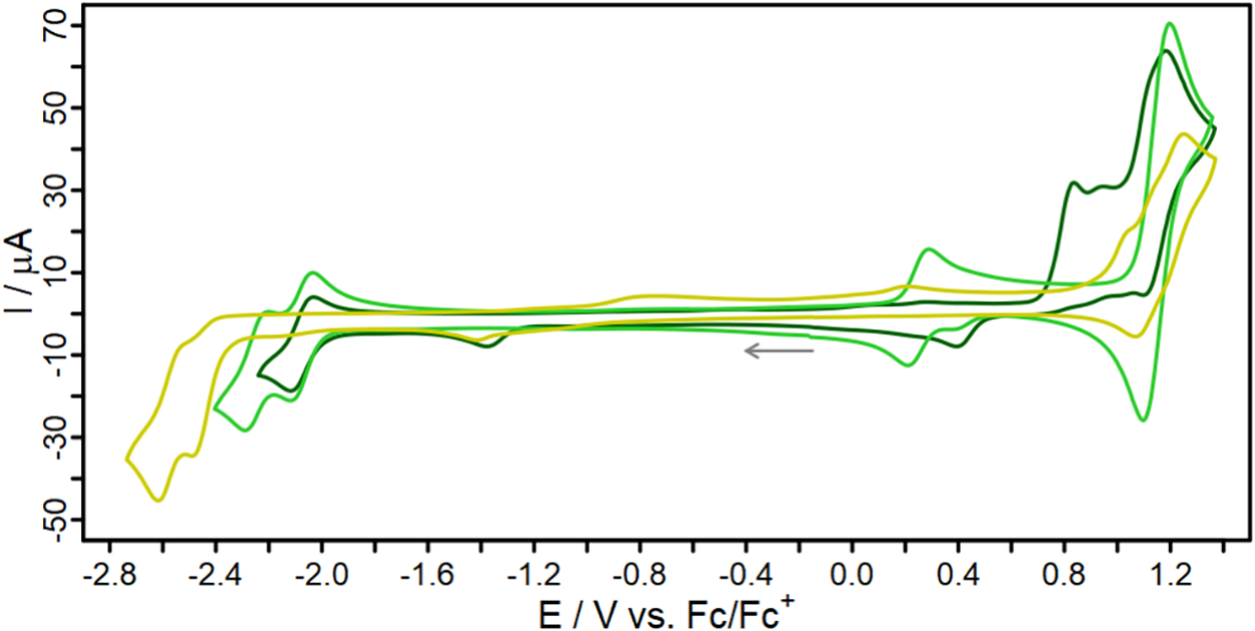
Cyclic voltammograms of the dyes **L1′** (yellow) **C1**′ (green) and **C2′** (dark green) in deaerated MeCN solution (*c* = 1 mM) with [Bu_4_N]­[PF_6_] (0.1 M) as supporting electrolyte referenced *vs*. the ferrocene/ferrocenium (Fc/Fc^+^) couple. The initial scan direction is marked by an arrow.

**Table 3:** Ground State Reduction and Oxidation Potentials of the Investigated Dyes **L1′**–**C2′** Obtained in Deaerated MeCN

	*E*^red^/V *vs*. Fc/Fc^+^	*E*^ox^/V *vs*. Fc/Fc^+^
**L1′**	–2.44 (irreversible)	0.99 (irreversible)
	–2.56 (irreversible)	
**C1′**	–2.06 (reversible)	0.23 (partially reversible)
	–2.24 (reversible)	
**C2′**	–2.07 (reversible)	0.78 (irreversible)

The ligand **L1′** shows an irreversible reduction at −2.44 V and an irreversible oxidation at 0.99 V (see Figure 5). The reduction potentials of the two complexes are anodically shifted (−2.06 V for **C1′** and −2.07 V for **C2′**) with respect to **L1′**. At the same time, the introduction of the Cu­(I) center causes a cathodic shift of the first oxidation event to 0.78 V for **C2′** (irreversible) and 0.23 V for **C1′** (partially reversible). This trend can be rationalized by predictions from the DFT calculations. The highest occupied molecular orbital (HOMO) of the complexes **C1′** and **C2′** is mainly localized at the copper center, while the HOMO of **L1′** is primarily located on one of the dimethoxyphenyl-substituents (see Figure S6.1). **L1′** and **C1′** both possess a second reduction in the scanned window (Table 3 and Figure 5). The appearance of this second reduction in **C1′** is most likely due to the reduction of the second phenanthroline ligand within the already singly reduced homoleptic complex (corresponding to the LUMO+1, Figure S6.3). Similar behavior has been reported previously.^63^ This process is absent in **C2′**, as this complex lacks the second phenanthroline. The second reduction in **L1′** is assigned to the reduction of a higher lying LUMO of the phenanthroline ligand. Notably, this second event can be observed in all compounds (**L1′**, **C1′**, and **C2′**) by differential pulse voltammetry (Figure S11.1). For **C1′**, this event reflects the reduction of the LUMO+2 (Figure S6.3), while in **C2′** it corresponds to the LUMO+1 (Figure S6.5).

The irreversible oxidation behavior of **C2′** can be explained by a dissociation of the diphosphine ligand, as the HOMO is partially localized on the phosphorus atoms. This irreversible oxidation is common for such heteroleptic Cu­(I) complexes.^20,26,52,64^

The significant difference between the oxidation potentials (Δ*E* = 0.55 V) of the complexes **C1′** and **C2′** is noteworthy. The higher oxidation potential of heteroleptic diimine-diphosphine complexes compared to their homoleptic bisdiimine counterparts is well-known and caused by the stabilization of the HOMO by π-backbonding of the diphosphine ligand as well as the higher steric hindrance destabilizing a square-planar geometry.^7,20^ Apart from that, the comparison of **C2′** with **CRef** ^26^ shows that the additional methoxy substituents have no major influence on the electrochemistry and the redox potentials (*E* ^red^ = −2.07 *vs*. −2.04 V).

### Binding of the Dyes to the TiO_2_ Surface

To assess their suitability as sensitizers for n-type DSSCs, the binding behavior of the dyes on TiO_2_ was first investigated. For this purpose, **L1**, **C1**, and **C2** were immobilized on TiO_2_ particles (AEROXIDE P25) by wet impregnation (see SI–Chapter 12 for further details) and subsequently studied by IR spectroscopy.^31,33,65^

The nonimmobilized ligand **L1** exhibits C–OH stretching vibrations at 1282, 1259, and 1239 cm^–1^, while the C–OH bending vibration of the catechol groups appears at 1362 cm^–1^ (*cf*. Figure S12.1).^66^ After immobilization of **L1** on TiO_2_, the C–OH stretching vibrations lose their hyperfine structure and merge into a single prominent band at 1285 cm^–1^. At the same time, the bending vibration decreases in intensity. Both observations strongly indicate covalent binding between the catechol anchor group and the TiO_2_ particles. A similar behavior has been reported in the literature for catechol–TiO_2_ composites.^66–68^

As the TiO_2_ substrates exhibit inherent O–H vibrations in the range above 3000 cm^–1^, it was not possible to determine whether the ligand binds to the TiO_2_ via one or both catechol anchors. The investigation of the respective complexes **C1** and **C2** revealed a similar IR absorption behavior (see Figures S12.2 and S12.3).

### Application in DSSCs with Organic Electrolytes

Encouraged by the experimental results described above, **L1**, **C1**, and **C2** were tested as sensitizers in TiO_2_-based DSSCs using organic electrolytes. Mesoporous TiO_2_ substrates were prepared by screen-printing TiO_2_ ink onto fluorine-doped tin oxide (FTO) glass, followed by sintering and dye sensitization in methanolic solutions of **L1**, **C1** or **C2** (see SI–Chapter 1 for full details).^69^ DSSCs were then assembled with platinum counter-electrodes and an electrolyte containing iodine/triiodide in MeCN.

Although the **L1** dyeing bath itself is colorless, deep red TiO_2_ substrates formed after one night of chemisorption (see inset Figure 6), indicating a type-II sensitization mechanism through strong electronic coupling between the catechol anchors and the TiO_2_ surface.^34,35,70^ The resulting **L1**-based DSSCs exhibited a PCE of 1.20% (Table 4), which is comparable to the best reported catechol-based DSSCs, particularly considering the open-circuit potential (*V*_oc_) of ≈500 mV.^38^ This relatively high *V*_oc_ may be attributed to the electron-withdrawing effect of the phenanthroline moiety, which is known to suppress charge recombination to some extent.^38,68,71^ However, charge collection efficiency remained a limiting factor, leading to a moderate short-circuit current (*J*_sc_) despite efficient light harvesting (Figure 6, top). Incident photon-to-current efficiency (IPCE) measurements confirmed the type-II charge injection mechanism, as photocurrent generation is dominated by the catechol-to-TiO_2_ transition (Figure 6, bottom). *J*_sc_ values obtained by integrating the IPCE were consistent with those extracted from *J*–*V* curves, confirming that photocurrent results from a photosensitization process (Table S14.5).

**Table 4:** Overview of the Performance Indicators Short-Circuit Current (*J*_sc_), Open-Circuit Potential (*V*_oc_), Fill Factor (FF) and Photoconversion Efficiency (PCE) of the DSSCs Using **L1**, **C1**, and **C2** as Photosensitizers with Two Different Organic Electrolytes E1 and E2^a^,^b^

	electrolyte	*J*_sc_/mA·cm^–2^	*V*_oc_/mV	FF/%	PCE/%
**L1**	E1	3.86 ± 0.14	496 ± 8	63 ± 1	1.20 ± 0.04
**C1**	E1	1.33 ± 0.01	523 ± 3	69 ± 3	0.48 ± 0.01
	E2	1.58 ± 0.01	512 ± 1	68 ± 1	0.55 ± 0.01
**C2**	E1	4.60 ± 0.12	493 ± 3	67 ± 1	1.53 ± 0.03
	E2	5.18 ± 0.28	468 ± 22	68 ± 1	1.64 ± 0.00
	E2 + 0.5 M TBP	4.40 ± 0.10	604 ± 1	71 ± 1	1.88 ± 0.05
N719	E1	12.62 ± 0.14	471 ± 2	66 ± 0	3.93 ± 0.07

a For further conditions and additives see SI–Chapter 14.

b E1: LiI (100 mM), I_2_ (50 mM), 1,2-dimethyl-3-ethylimidazolium iodide (600 mM) in acetonitrile. E2: LiI (100 mM), I_2_ (50 mM), 1,2-dimethyl-3-ethylimidazolium iodide (600 mM), guanidinium thiocyanate (100 mM) in acetonitrile. Values for the reference dye N719 (ditetrabutylammonium *cis*-bis­(isothiocyanato)­bis­(2,2′-bipyridyl-4,4′-dicarboxylato) ruthenium­(II)) are given for comparison.

**Figure 6:**
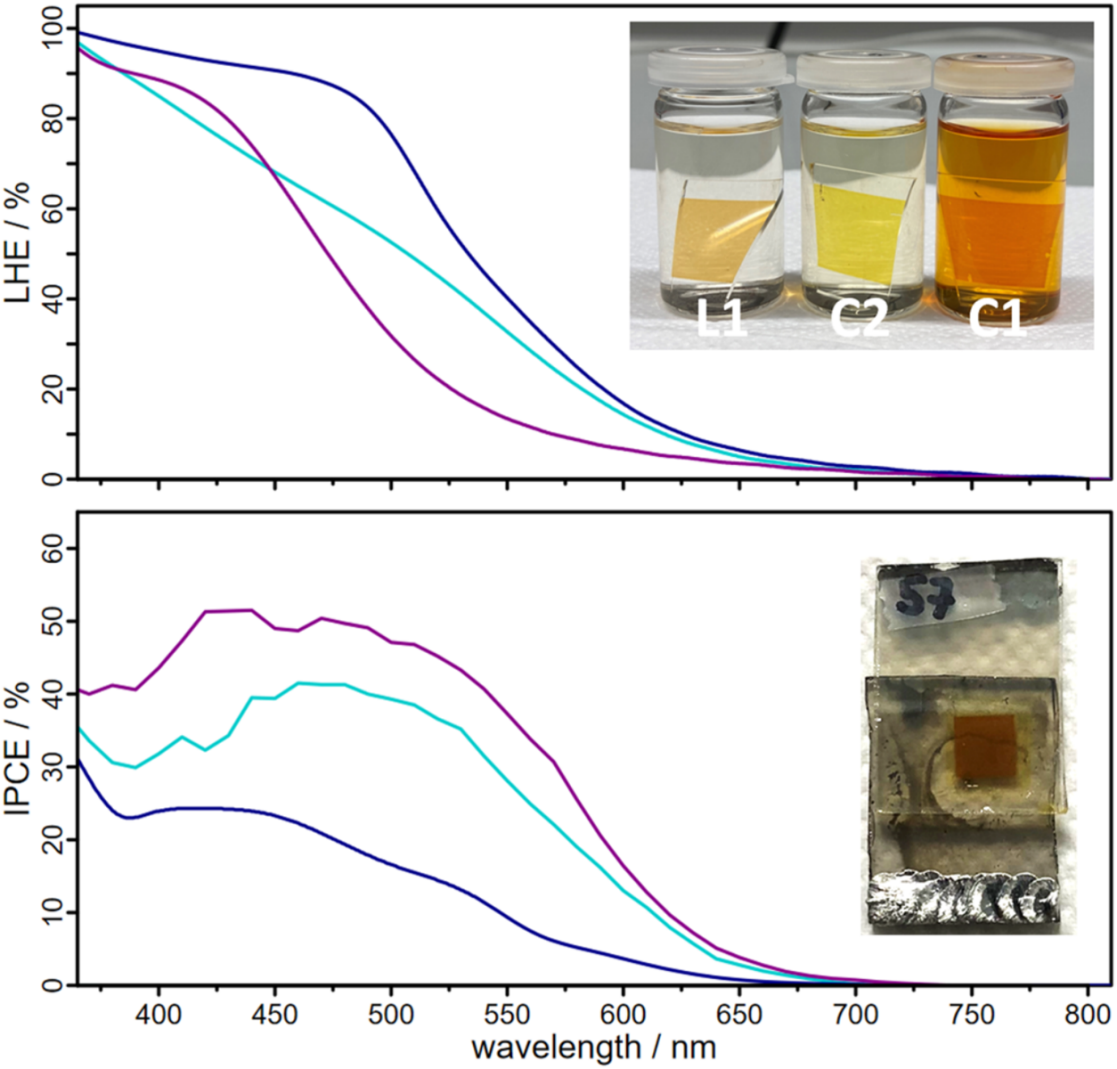
Light-harvesting efficiencies (LHE, top) and incident photon-to-current efficiencies (IPCE, bottom) of the assembled DSSCs based on **L1** (light blue), **C1** (dark blue) and **C2** (magenta). IPCE measurements were performed on cells containing **L1** with E1 as electrolyte and on cells with **C1** and **C2** with E2 as electrolyte. The inset shows substrates for the determination of the LHE of the respective dyes **L1**, **C1**, and **C2** after one night of chemisorption (top) and a **C2**-based DSSC (bottom).

The influence of electrolyte additives on the DSSC performance was then investigated. Adding 0.5 M 4-*tert*-butylpyridine (TBP) drastically reduced *J*_sc_ from 3.60 to 1.17 mA·cm^–2^, while *V*_oc_ improved by 90 mV (Table S14.1). This effect is attributed to TBP adsorption on the TiO_2_ surface, inducing a negative conduction band shift.^72–74^ While this process raises the Fermi level, it simultaneously reduces the electron injection driving force, ultimately leading to a net decrease in overall efficiency. Even at a lower TBP concentration (0.1 M), *J*_sc_ was more than halved, confirming the detrimental effect of TBP in **L1**-based DSSCs.

Polyaromatic dyes are prone to aggregation on TiO_2_, which can lead to self-quenching of the excited state. To mitigate this effect, chenodeoxycholic acid (CDCA)^75–78^ was tested as an antiaggregation additive. However, no significant improvement was observed in **L1**-based DSSCs (see Table S14.2). Increasing CDCA concentrations up to 20 mM had no effect, while 100 mM CDCA drastically reduced *J*_sc_ due to lower LHE (see Figure S14.1). Overall, under optimized conditions, the best **L1**-based DSSC achieved: *J*_sc_ = 3.95 mA·cm^–2^, *V*_oc_ = 502 mV, fill factor (FF) = 62% and PCE = 1.24%, which classifies **L1** as an efficient dye for type-II DSSCs.

### C1- and C2-Based DSSCs with Organic Electrolytes

DSSCs using **C1** and **C2** as sensitizers were assembled under identical conditions, revealing markedly different performances. While **C1**-based DSSCs exhibited low *J*_sc_ and poor overall efficiency,^9^ **C2**-based DSSCs, which employ a heteroleptic Cu­(I) complex, significantly outperformed both **C1**- and **L1**-based devices, particularly in terms of *J*_sc_ (Table 4).

The addition of guanidinium thiocyanate (GuSCN) and TBP as electrolyte additives further enhanced the performance of **C2**-based DSSCs. GuSCN is known to interact with TiO_2_ surfaces and dye monolayers, reducing recombination rates and improving electron collection efficiency.^79–83^ In the case of **C2**, GuSCN increased the *J*_sc_ from 4.60 to 5.18 mA/cm^2^ in the presence of 0.1 M TBP. Additionally, TBP gradually improved *V*_oc_, albeit at the cost of a slight *J*_sc_ reduction (Table S14.3). Conveniently, this compromise resulted in an overall efficiency increase, with PCE reaching 1.88%. Notably, GuSCN had no beneficial effect in **L1**-based DSSCs (see Table S14.1).

As in **L1**-based DSSCs, IPCE measurements confirmed a type-II charge injection mechanism for **C2**-based devices. Despite its limited visible-light absorption, similar to other heteroleptic diimine-diphosphine Cu­(I) complexes,^7,20,21^ the **C2**-sensitized TiO_2_ electrodes displayed a significantly broadened absorption onset (≈600 nm, Figure 6), confirming strong catechol-to-TiO_2_ charge transfer. Interestingly, while CDCA had no effect on **L1**-based DSSCs, small amounts (0.5 mM) in **C2** dyeing baths drastically reduced *J*_sc_ (see Table S14.4). This is attributed to a decrease in LHE, as confirmed by the lower absorbance of TiO_2_ substrates dyed with **C2** in the presence of CDCA (see Figure S14.2). Finally, the best-performing **C2**-based DSSC achieved: *J*_sc_ = 4.50 mA·cm^–2^, *V*_oc_ = 605 mV, FF = 71% and PCE = 1.93%. These results mark the highest reported performance for a DSSC using a heteroleptic Cu­(I) complex, representing a 35-fold increase in PCE compared to previous diimine-diphosphine Cu­(I) DSSCs (PCE = 0.053%).^22^ Notably, the high *V*_oc_ of 605 mV is among the best values reported for type-II DSSCs.^38^

The superior efficiency of **C2**-based DSSCs compared to **L1**-based DSSCs is somewhat unexpected, as **L1** exhibits superior LHE (Figure 6). However, several factors likely contribute to this effect: (i) IPCE spectra of **L1**- and **C2**-based DSSCs share similar profiles, but **C2** exhibits an additional photocurrent contribution around 400 nm, likely originating from Cu­(I)-to-**L1** MLCT transitions. (ii) The presence of Cu­(I) within the **L1** cavity may enhance the electron-withdrawing effect of the catechol groups, thereby improving charge collection efficiency. This is confirmed by electrochemical impedance spectroscopy (EIS). EIS is a powerful tool to analyze the interfacial charge transfers in DSSCs of all kinds.^84–88^ Indeed, the Nyquist diagram of the **C2**-based device is dominated by the middle-frequency semicircle, corresponding to heterogeneous electron transfer at the TiO_2_–**C2**/electrolyte interface (Figure S14.3) and associated with charge recombination at this interface (*R*_rec_). In contrast, the Nyquist plot for **L1**-based DSSC exhibits a distorted profile, which is typically observed for DSSCs with strong charge recombination processes.^89^ Electron lifetimes (τ_e_ = 9.4 ms for **L1**-based and 13.9 ms for **C2**-based DSSCs, see Figure S14.4) estimated from the Bode spectra, further confirm the enhanced charge collection efficiency for **C2**-based DSSCs. Given the rather large *V*_oc_ of **L1**-based DSSC, and the fact that both **L1**- and **C2**-based DSSCs exhibit similar *R*_rec_ values, we hypothesize that these observations may be due to geminate charge recombination (*i.e*., recombination between e_(CBTiO_2_)_^–^ and oxidized dye **L1** ^+^). These factors provide a plausible explanation for the better performance of **C2**-based DSSCs, despite the higher LHE of **L1**.

In contrast, the catechol anchor appears unsuitable for homoleptic Cu­(I) complexes, as **C1**-based DSSCs possess significantly lower *J*_sc_ than **C2**-based DSSCs. This cannot be attributed to differences in LHE, as **C1**-sensitized photoanodes display a comparable or even red-shifted absorption compared to **C2** or **L1**. The underlying reason remains unclear, but several key differences between **C1** and **C2** may help to explain this observation. As a heteroleptic complex, **C2** is anchored to TiO_2_ via the phenanthroline ligand **L1**, which results in a built-in dipole that facilitates photoinduced electron injection.^90^ On the contrary, the homoleptic bisdiimine complex **C1** lacks this charge transfer vectorization, as confirmed by frontier molecular orbital calculations (see Figures S6.4 and S6.6). While the LUMO of **C2** is almost exclusively localized on **L1**, in **C1** it is distributed over both ligands, which likely reduces electronic coupling with TiO_2_. This was corroborated by EIS measurements, revealing that **C1**-based DSSC exhibited (like **L1**-based DSSC, see above) a distorted Nyquist spectrum (Figure S14.5), which is characteristic of strong charge recombination. The larger *R*_rec_ observed for **C1**-DSSC is in line with a weaker *J*_sc_, indicating that recombination is not the only factor limiting device performance. The electron lifetimes (τ_e_ = 1.3 ms for **C1**-based and 9.4 ms for **C2**-based DSSC, see Figure S14.6) confirm the superior charge collection efficiency of the heteroleptic system. The satisfying *V*_oc_ of these devices points toward favored geminate charge recombination for **C1**-based devices.

Moreover, discussing the frontier orbitals of the isolated molecules alone is insufficient in the context of type-II sensitization, as it neglects the new electronic states arising from catechol-TiO_2_ interactions. We propose that the dyes in this study function as a dual-chromophore system, comprising a primary TiO_2_–catechol (TiO_2_–Cat) chromophore and a secondary Cu­(I) complex antenna chromophore (either with **C1** or **C2**). The adsorbed dyes can thus be described as TiO_2_–Cat-**C1** and TiO_2_–Cat-**C2** (Figure 7). Upon photoexcitation of the TiO_2_–Cat chromophore, a charge-transfer state TiO_2_^–^–Cat^+^ is formed. The energy of this state can be estimated from the difference between the onset oxidation potential of the TiO_2_–Cat chromophore and the conduction band potential of TiO_2_ (−0.7 V *vs*. SCE). The oxidation potential of TiO_2_–Cat is nearly identical for TiO_2_–Cat-**C1** and TiO_2_–Cat-**C2** (see Figure S14.7), indicating minimal influence of **C1** or **C2** on TiO_2_–Cat energetics. Based on cyclic voltammetry measurements of **C1** and **C2** adsorbed on TiO_2_ (see Figure S14.7), the energy of the TiO_2_^–^–Cat^+^ state is estimated to be ≈2.2 eV. Furthermore, the MLCT excited-state energies of **C1** and **C2** were estimated from the onset of their UV/vis absorption spectra (defined at an absorbance of 10^3^ M^–1^cm^–1^, *cf*. Figure S8.2), yielding values of 2.1 eV for **C1** and 2.6 eV for **C2**.

**Figure 7:**
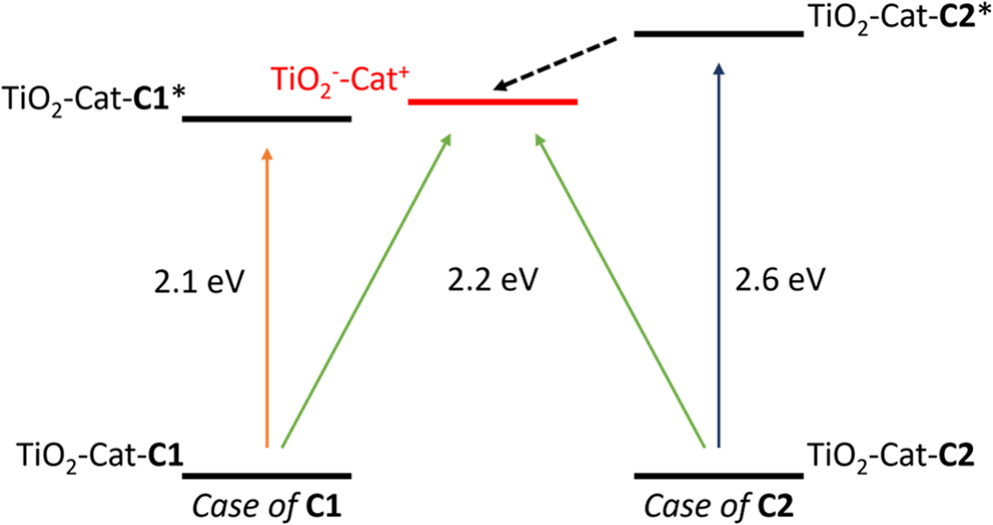
Qualitative energy diagram depicting the respective energies of the TiO_2_^–^–Cat^+^ state (red) and the TiO_2_–Cat-C1* (left) and TiO_2_–Cat-C2* (right) MLCT excited states. Cat = catechol.

Using these values, a qualitative energy diagram was constructed (Figure 7) to illustrate the relative positioning of the different excited and charge-transfer states.

This diagram reveals the excited state of TiO_2_–Cat-C2* to lie above the TiO_2_^–^–Cat^+^ state, enabling a beneficial energy transfer from **C2** to TiO_2_^–^–Cat^+^. In contrast, energy transfer from **C1** to TiO_2_^–^–Cat^+^ is not favorable, as the TiO_2_–Cat-C1* excited state is already lower in energy. Furthermore, direct electron injection from **C1** into the TiO_2_ conduction band (type-I injection) is unlikely due to the lack of LUMO electron density on the anchor groups (*cf*. Figure S6.4), indicating poor electronic coupling between the excited **C1** complex and TiO_2_. In addition, the probably short excited-state lifetime of C1* does not favor type-I charge injection. These insights rationalize that energy transfer from TiO_2_^–^–Cat^+^ to **C1** would lead to a loss of the excited state and could explain the lower performances of **C1**-DSSC compared to **C2**- and **L1**-based DSSCs.

### DSSCs with Aqueous Electrolyte

Catechol groups exhibit significantly stronger anchoring to TiO_2_ than classical carboxylic acids.^37–42^ This was demonstrated by desorption experiments, where **L1**-dyed TiO_2_ photoanodes could not be desorbed even under extreme conditions, including concentrated sulfuric acid and 10 M NaOH in DMF. Given this exceptional binding stability, **L1** and **C2** were investigated as photosensitizers in DSSCs employing an aqueous electrolyte.

Aqueous electrolytes offer key advantages over conventional organic electrolytes, including nonflammability, lower toxicity and improved long-term stability by reducing sealing-related degradation. Although water has historically been considered detrimental to DSSC performance, recent studies have re-evaluated its potential as a renewable electrolyte solvent.^91–93^

To explore this approach, **L1** and **C2** were tested with the previously published optimized aqueous electrolyte E3 (*i.e*., 4 M KI, 20 mM I_2_ in water saturated with CDCA).^94^ The high iodide concentration was necessary to enhance the I_3_^–^ concentration, because the binding between I^–^ and I_2_ is less efficient in aqueous environment.^95^ Additionally, CDCA was added as a surfactant to improve the wetting of the TiO_2_ pores, which tends to be inefficient due to the hydrophobic nature of the dye monolayer.^91,96^

As shown in Table 5, both **L1**- and **C2**-based DSSCs successfully operated with the aqueous electrolyte, albeit with moderate efficiencies (PCE = 0.21 and 0.28%). The lower efficiencies compared to organic-electrolyte DSSCs are consistent with known challenges associated with water-based DSSCs,^92,96^ such as incomplete TiO_2_ wetting and increased charge recombination between I_2_ and conduction-band electrons.

**Table 5:** Overall Performances of DSSCs Using **L1** and **C2** as Photosensitizers and the Aqueous Electrolyte E3^a^,^b^

	electrolyte	*J*_sc_/mA·cm^–2^	*V*_oc_/mV	FF/%	PCE/%
**L1**	E3	0.73 ± 0.06	488 ± 18	59 ± 2	0.21 ± 0.03
**C2**	E3	1.15 ± 0.13	404 ± 9	60 ± 6	0.28 ± 0.07
N719	E3	1.78 ± 0.21	428 ± 5	60 ± 1	0.46 ± 0.06

a The reference dye N719 is given for comparison.

b E3: KI (4 M), I_2_ (20 mM) in water saturated with CDCA.

However, two key observations should be emphasized: (i) Despite the moderate PCE values, these DSSCs outperformed previously reported heteroleptic Cu­(I)-based DSSCs employing organic electrolytes, which achieved significantly lower efficiencies (e.g., a PCE of 0.053%).^22^ (ii) Exceptional device stability was observed. As shown in Figure 8 the performance of aqueous DSSCs remained stable for at least 10 days, whereas DSSCs based on the well-known N719 dye degraded quickly under identical conditions (see SI–Chapter 14 for further details). This finding underscores the benefits of catechol anchors for long-term DSSC stability.

**Figure 8:**
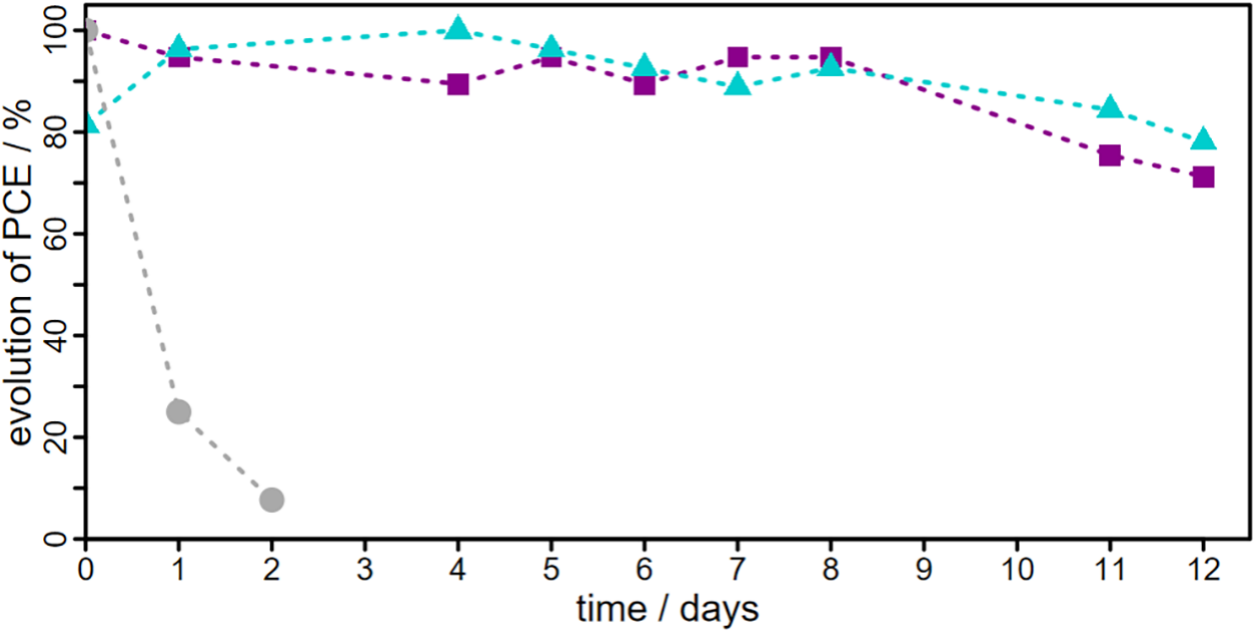
Relative development of the PCE over time for DSSCs using the aqueous electrolyte E3 and **L1** (light blue, triangles), **C2** (magenta, squares) or N719 (gray, circles) as sensitizer.

## Conclusions

This work presents the preparation and systematic investigation of a new class of dye-sensitized solar cells (DSSCs) based on Cu­(I) complexes with catechol anchor groups. The goal was to develop a sustainable alternative to noble metal-based sensitizers and to explore the influence of catechol groups on the performance and stability of these systems.

To this end, a series of novel catechol dyes was synthesized by introducing two catechol units at the 4,7-positions of 2,9-dimethyl-1,10-phenanthroline. The study encompassed not only the pure organic dye **L1**, but also the corresponding homoleptic (**C1**) and heteroleptic (**C2**) Cu­(I) complexes. Their properties were systematically compared with the methoxy-protected precursors and tested as sensitizers in DSSCs.

The introduction of the electron-donating catechol groups significantly improved the photophysical properties with respect to the unsubstituted reference dyes. While the homoleptic complex **C1** has a much better absorptivity in the visible (λ_max_ = 476 nm) compared to its heteroleptic counterpart, it does not show emission. In contrast, the heteroleptic complex **C2** exhibits a broad emission around 565 nm, with a favorable lifetime τ_em_ of 6.5 μs and a quantum yield of 2.9% in methanol solution. Otherwise, the analyses of the electrochemical and structural properties show no significant changes due to the additional catechol groups.

DSSC performance studies demonstrated that the organic dye **L1** already achieved a moderate photoconversion efficiency (PCE) of 1.20 ± 0.04% with an open-circuit potential (*V*_oc_) of nearly 500 mV in organic electrolyte systems. The heteroleptic Cu­(I) complex **C2** outperformed previously reported Cu­(I)-based DSSCs, reaching a PCE of 1.53 ± 0.03%, which was further enhanced to 1.88 ± 0.03% in the presence of guanidinium thiocyanate and 4-*tert*-butylpyridine as additives. This represents a 35-fold improvement over the best DSSCs based on heteroleptic Cu­(I) complexes reported to date. Incident photon-to-current efficiency (IPCE) measurements confirmed efficient photoinduced charge injection and support the proposed injection mechanism. The superior performance of the heteroleptic **C2**-based DSSCs is further supported by electrochemical impedance spectroscopy (EIS), revealing longer electron lifetimes and reduced charge recombination compared to **L1**- and **C1**-based devices.

A particularly noteworthy finding was that both **L1** and **C2** successfully operated in DSSCs with aqueous electrolytes, maintaining their performance for at least 10 days. This highlights the strong binding affinity and long-term stability, underlining their potential for sustainable DSSC applications.

Mechanistic insights based on IPCE spectra, electrochemical data, and frontier orbital analyses support a type-II injection mechanism for all three catechol dyes. We propose that the working principle of these DSSCs can be interpreted as a dual-chromophore system, where the TiO_2_-catechol interaction serves as the primary chromophore, while the Cu­(I) complex acts as an additional antenna chromophore.

Overall, this study demonstrates the feasibility of using Cu­(I) complexes with catechol anchors as effective DSSC sensitizers, particularly in aqueous electrolyte systems. Catechol functionalization provides a powerful strategy to enhance the photophysical properties, stability, and performance of Cu­(I)-based DSSCs. Future studies should focus on optimizing the electronic properties of these dyes and exploring alternative electrolyte systems to push the efficiency limits of Cu­(I)-sensitized DSSCs even further.

## Supplementary Material



## Notes

Experimental, synthetic, computational, spectroscopic and electrochemical details, along with information on the fabricated dye-sensitized solar cells (PDF)

## ∥

Department of Chemistry, University of Basel, St. Johanns-Ring 19, 4056 Basel, Switzerland
