# Supplementary material for: Dye-Sensitized Solar Cells Based on Cu(I) Complexes Containing Catechol Anchor Groups That Operate with Aqueous Electrolytes
Source: JACS Au. 2025 Jul 29;5(8):3960–73. doi: 10.1021/jacsau.5c00601 (PMC12381701; doi:10.1021/jacsau.5c00601)
Supplement: Supplementary file 1 [file au5c00601_si_001.pdf]

Supporting Information

Dye-Sensitized Solar Cells Based on Cu(I) Complexes Containing Catechol Anchor Groups that Operate with Aqueous Electrolytes

Lars E. Burmeister,[1,‡] Florian Doettinger,[1,‡,§] Kurt J. Haseloff,[1] Christian Kleeberg,[2] Mohammed Boujtita,[3] Simon Pascal,[3] Fabrice Odobel,[3] Stefanie Tschierlei,[1] Yann Pellegrin,[3,] Michael Karnahl[1,*]*

[1] Department of Energy Conversion, Institute of Physical and Theoretical Chemistry, Technische Universität Braunschweig, Rebenring 31, 38106 Braunschweig, Germany.
[2] Institute of Inorganic and Analytical Chemistry, Technische Universität Braunschweig, Hagenring 30, 38106 Braunschweig, Germany.
[3] Nantes Université, CNRS, CEISAM, UMR 6230, F-44000 Nantes, France.
[§] Present Address: Department of Chemistry, University of Basel, St. Johanns-Ring 18, 4056 Basel, Switzerland

[‡]These authors have contributed equally to this work and share first authorship.
*Correspondence:
Dr. Yann Pellegrin: yann.pellegrin@univ-nantes.fr
Dr. Michael Karnahl: michael.karnahl@tu-bs.de

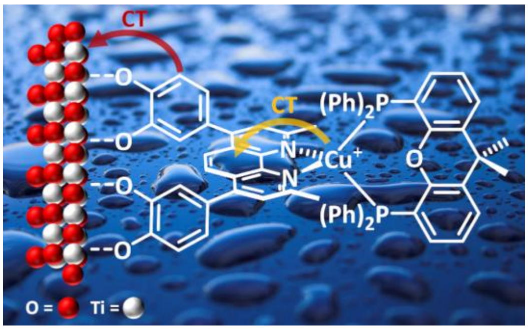

Supporting Information - Table of Contents

1	Experimental details	page 2
2	Synthetic details	page 6
3	NMR spectra	page 13
4	MS spectra	page 23
5	Crystallographic data and solid-state molecular structure of C2'	page 29
6	Density functional theory (DFT)	page 30
7	Time-dependent density functional theory (TDDFT)	page 36
8	UV/vis absorption	page 51
9	Steady-state emission	page 52
10	Time-resolved emission	page 54
11	Electrochemical data	page 55
12	IR spectroscopy	page 56
13	Photostability	page 57
14	Dye-sensitized solar cells	page 58
15	References	page 65

1 Experimental details

NMR spectroscopy. Nuclear magnetic resonance (NMR) spectra were recorded on Bruker series spectrometer of the type Avance III™ HD 500 at frequencies of 500 MHz (^{1}H), 125 MHz (^{13}C) and 202 MHz (^{31}P) by the analytical service of the Institute of Inorganic and Analytical Chemistry at the Technische Universität Braunschweig. The chemical shift δ is given consecutively in parts per million ppm, where $\delta = 0$ corresponds to the internal standard tetramethylsilane (TMS). The NMR solvents used are indicated in each spectrum. All measurements were performed using Wilmad type 528-PP NMR tubes. The signal multiplicities are given sequentially as: *s* (singlet), *d* (doublet), *t* (triplet), *q* (quartet), *sept* (septet), *m* (multiplet). Coupling constants are given in Hz. The obtained spectra were analyzed utilizing the TopSpin software (4.2.0).

Mass spectrometry. Mass spectrometry (MS) measurements were performed by the MS department of the Institute of Inorganic and Analytical Chemistry at the Technische Universität Braunschweig. High-resolution mass spectra were obtained via electrospray ionization (ESI) utilizing an LTQ-Orbitrap Velos Orbitrap mass analyzer manufactured by ThermoFisher Scientific. Mass spectrometry values are given in terms of mass-to-charge ratio (*m/z*).

X-ray diffraction studies. The single crystals were transferred into inert perfluoroether mounted on top of a *CryoLoop* (*Hampton Research*) and placed on the diffractometer in the cold nitrogen gas stream of a *Cryostream 800* cooling system (*Oxford Cryosystems*). The data were collected on a *Rigaku Oxford Diffraction Synergy-S* instrument using either mirror-focused MoKa radiation (*Rigaku PhotonJet* microfocus source). The reflections were indexed, integrated and appropriate absorption corrections were applied as implemented in the CrysAlisPro software package.[1] The structures were solved employing the program SHELXT and refined anisotropically for all non-hydrogen atoms by full-matrix least squares on all F^2 using SHELXL software.[2–4] Hydrogen atoms were refined employing a riding model; methyl groups were treated as rigid bodies and were allowed to rotate about the E–CH_3 bond. During refinement and analysis of the crystallographic data the programs OLEX^2, PLATON, Mercury and Diamond were used.[5–8]
Supplementary crystallographic data can be found under CCDC 2431283. These data can be obtained free of charge by the joint Cambridge Crystallographic Data Centre and Fachinformationszentrum Karlsruhe via www.ccdc.cam.ac.uk/structures/ or by emailing data_request@ccdc.cam.ac.uk or by contacting The Cambridge Crystallographic Data Centre, 12 Union Road, Cambridge CB2 1EZ, UK.

(TD-)DFT calculations. Quantum chemical calculations of density functional theory (DFT) level were performed using the ORCA program package (Version 5.0.3).[9] Geometry optimizations were conducted first using the BP86[10] exchange-correlation functional to perform pre-optimization. Second, the B3LYP[11] hybrid functional was used for final optimization. TD-DFT calculations obtaining theoretical UV/vis spectra and difference density plots were treated similarly. To account for dispersion effects, the D3 correction by S. Grimme including the Becke-Johnson (BJ) damping[12,13] was utilized. As basis sets the Karlsruhe's valence triple-zeta polarization functions basis sets (def2-TZVP) were applied.[14] Solvation effects were accounted for by the conductor-like polarizable continuum model CPCM for acetonitrile.[15]

Optimized geometries were verified as minima on the potential energy surface by frequency calculations (analytical, B3LYP-D3(BJ)/def2-tzvp, CPCM). For all calculations the PF_6^- counterion was neglected. Visualizations of the molecular orbitals and of the electron difference density plots were evoked using the Chemcraft software package (Version 1.8).[16]

Steady-state UV/vis absorption. UV/vis absorption spectra were obtained using a JASCO V-770 spectrophotometer. The compounds were dissolved in acetonitrile or methanol (hplc grade) and the spectra were recorded applying a standard 10 mm fluorescence quartz glass cuvette. All spectra are baseline corrected at 800 nm.

Steady-state emission. Emission spectra were recorded with a Horiba Jobin-Yvon FluoroMax Plus-C emission spectrometer. All samples were measured in dry acetonitrile or methanol under inert conditions using a sealed 10 mm fluorescence quartz glass cuvette. Optical densities (OD) were around 0.1 at the respective excitation wavelength. The emission quantum yields were calculated using the following equation (eq. S1.1).[17]

$$\Phi_c = \Phi_R \left(\frac{\eta_C^2}{\eta_R^2}\right)\left(\frac{A_R}{A_C}\right)\left(\frac{I_C}{I_R}\right) \qquad \text{eq. S1.1}$$

The quantum yield of the compound (Φ_c) is calculated using the refractive indices of the solvents for the compound (η_C) and the reference (η_R), as well as the absorbance of the compound (A_C) and the reference (A_R), along with the integral of the compound's emission (I_C) and the reference's emission (I_R). The quantum yield of the ligands was calculated with pyrene ($\phi = 0.62$) in inert acetonitrile as reference at an excitation wavelength of 310 nm.[18] The emission quantum yield of the heteroleptic complexes was calculated with [Cu(xant)(bathocuproine)]PF_6 ($\phi = 0.0135$) as reference at an excitation wavelength of 400 nm.[19] The refractive indices of acetonitrile ($\eta = 1.3449$) and of methanol ($\eta = 1.3279$) were used.[20]

Time-resolved emission. Emission lifetimes were measured utilizing a Q-switched pulsed Nd:YAG laser system with excitation pulses of approximately 6 ns centered at 355 nm. The laser power was adjusted to around 1.0 mJ per pulse in front of the sample holder. Emission was detected using a photomultiplier tube integrated with an Edinburgh Instruments LP980 spectrometer at the respective emission maximum of the sample. All emission lifetimes were recorded under oxygen free conditions in dry acetonitrile and methanol in sealed 10 mm fluorescence quartz glass cuvettes at room temperature. The optical density of the respective solutions was adjusted to 0.1 ± 0.01 at the excitation wavelength.

Electrochemistry. Cyclic voltammetry and differential pulse voltammetry were obtained using an Autolab potentiostat PGSTAT204 from Metrohm. Measurements were acquired in deaerated acetonitrile with an analyte concentration of 1 mM and $[Bu_4N][PF_6]$ (0.1 M) as the supporting electrolyte. A three-electrode configuration consisting of a glassy carbon disc ($\varnothing = 3$ mm) as a working electrode, a platinum wire as a counter electrode and a non-aqueous Ag/Ag^+ reference electrode was used. Unless stated otherwise. For cyclic voltammetry the scan rate was set to 100 mV/s. Differential pulse voltammetry was measured using steps of 10 mV with an integration time of 0.5 s. All data are referenced against the ferrocene/ferrocenium (Fc/Fc^+) couple, by adding ferrocene to the solution after each measurement. All reduction and oxidation potentials were obtained from differential pulse

voltammograms. The reversibility of the respective events was determined from cyclic voltammograms.

IR spectroscopy. IR spectroscopy was carried out using a JASCO FT/IR-4700 Fourier-transform infrared spectrometer. The compounds were measured as KBr pellets. For the measurement of TiO_2 composites, the respective dyes were immobilized in TiO_2 particles (AEROXIDE® TiO_2 P 25, EVONIK) by wet impregnation in methanol.

Photostability. UV/vis absorption spectra were conducted using an Avantes AvaSpec-ULS2048CL-EVO-RS spectrophotometer. A 150 W xenon arc lamp (LOT-QuantumDesign GmbH, LSE140/160.25C) was operated at 112 W and used as light source. Two OD filters (0.5 and 0.1) were introduced additionally to a cut-off filter (380 nm) to adjust the light intensity at the cuvette to around 1000 W/m^2. The measurements were carried out under ambient conditions in a sealed quartz glass cuvette (10 mm) equipped with a magnetic stir bar. Samples were dissolved in acetonitrile (c = 0.02 mM, V = 3 mL) and stirred at 400 rpm.

Light harvesting efficiencies. To estimate the light harvesting efficiencies of the dyes, glass slides were coated with one layer of TiO_2 and coated with the respective dyes following the same procedure as for DSSCs. The optical density (OD) of the resulting slides was measured and the LHE was calculated using eq. S1.2.[21]

$$\mathrm{LHE}(\lambda) = 1 - 10^{\mathrm{OD}(\lambda)} \qquad \text{eq. S1.2}$$

DSSC fabrication. DSSCs were assembled by adapting a literature known procedure.[22] For the preparation of the DSSCs, fluorine doped tin oxide (FTO) conductive glass substrates (SOLARONIX) were cut into 2 x 1.5 cm pieces and washed in ethanol and acetone, followed by successive sonication in soapy water and in an 0.1 M ethanolic solution of HCl (10 min each). FTO substrates were first air dried and then heated to 375°C for 30 min. The conductive glasses were immersed in an aqueous $TiCl_4$ solution at about 75°C for 15 min. The substrates were then washed with ethanol and dried in air. Three TiO_2 layers were screen printed using transparent colloidal paste and a final light-scattering layer was added. Each layer was dried for around 15 minutes at 80°C. The obtained substrates were sintered following a gradual heating ramp (Table S1.1).

Table S1.1. Specific temperatures and heating gradients applied for sintering of the FTO substrates.

Final temperature / °C (holding time / min)	75 (30)	135 (15)	325 (5)	375 (5)	450 (30)
Heating gradient / °C/min	5	6	19	10	15

The FTO substrates were allowed to cool down, immersed in an aqueous $TiCl_4$ solution for 30 min at around 75°C, washed with ethanol, air-dried and sintered again. After sintering, the still hot FTO substrates (*ca.* 80°C) were dip-coated over night in a 0.1 mM methanolic solution of the respective dye. The freshly dyed substrates were washed with ethanol and dried under a N_2-stream. Solar cells were prepared using the dye-sensitized electrodes as the working electrodes and platinum-coated conducting glass electrodes as counter electrodes. The latter were prepared by chemical deposition of platinum from hexachloroplatinic acid in isopropanol

(10 mg/mL) and subsequent heating at 450°C for 30 minutes. The two electrodes were glued together using a thin transparent film of Surlyn™ polymer (DuPont, 25 µm) as a spacer. The electrolyte was introduced by vacuum back filling through a predrilled hole in the counter electrode. The photovoltaic device was sealed afterwards with Surlyn™ and a cover glass. The active cell area was 0.25 cm^2.

Electrochemical impedance spectroscopy. Electrochemical impedance spectroscopy (EIS) experiments were carried out on the DSSC prepared with **L1**, **C1** and **C2** with electrolytes E1 or E2, to access the charge transfer of the recombination process with a Potentiostat model VSP from Bio-Logic Science Instruments. The DSSC was examined under 1 sun illumination at V_{oc} in the frequency range of 1 kHz to 100 mHz. The photoanodes were connected to the working electrode and the cathode to the counter and reference electrodes.
All values of bias voltage were corrected by subtracting IR ohmic drop. The EIS data was fitted using Zim-Fit (Bio-Logic Science Instrument France) analysis software, with the classical transmission line as model.[23] From the fittings of the Nyquist plots, series resistance (R_s), transport resistance (R_{tr}) and recombination resistance (R_{rec}) were extracted.

2 Synthetic details

Reactions. Air-sensitive and/or moisture-sensitive reactions were carried out in dry glass vessels according to the standard Schlenk technique. For this purpose, the vessels were each heated *in vacuo* at 500 °C and then purged with argon three times.

Thin layer chromatography. Aluminum DC plates (silica matrix) were used to determine the reaction progress and an eluent system for chromatographic purification and were visualized by UV detection (λ = 365 nm).

Column chromatography. For column chromatography, silica gel or aluminum oxide were used as stationary phase. The used solvents were collected and recycled whenever possible. Overpressure was generated by hand using a Peleus ball.

Chemicals and solvents. Solvents and chemicals used were purchased from commercial suppliers (*e.g.* Sigma-Aldrich, Carl-Roth, Merck (TCI), Acros Organics) while some starting materials were self-prepared (explicitly stated). Dry and deoxygenated solvents for complex synthesis were obtained *via* distillation. Deoxygenated water was obtained in a Schlenk flask under bubbling through with argon for several hours while stirring.

Synthesis of L1'

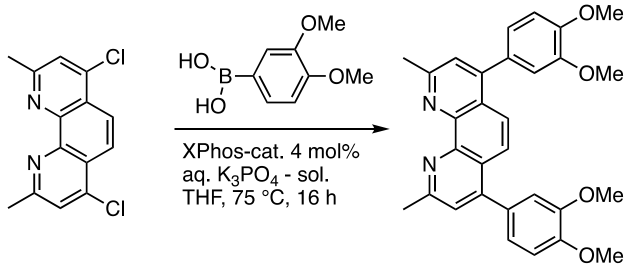

In a dried Schlenk flask equipped with a magnetic stir bar, 4,7-dichloro-2,9-dimethyl-1,10-phenanthroline (160 mg, 0.577 mmol, 1 eq.), 3,4-dimethoxyboronic acid (315 mg, 1.731 mmol, 3 eq.), XPhos-Pd-G2 (17.8 mg, 0.023 mmol, 4 mol-%), and K_3PO_4 (2.08 g, 9.815 mmol, 17 eq.) were combined. The flask was connected to a reflux condenser and the whole apparatus was evacuated and purged with argon three times. Subsequently, dry THF (14 mL) and degassed H_2O (16 mL) were added *via* syringe. The reaction mixture was stirred for 16 hours at 75 °C. Following, THF was removed under reduced pressure. The remaining aqueous phase was subjected to five washes with 2 M aq. NaOH-solution (5 x 20 mL) and DCM (3 x 20 mL). The combined organic phases were dried over $MgSO_4$ and solvent was evaporated under reduced pressure. The crude product was recrystallized from a boiling mixture of DCM and *n*-heptane (ratio 1:30-1:50), filtered, washed with *n*-heptane and stored at 6 °C overnight. After filtration and drying *in vacuo*, the product was obtained as a white solid (220 mg, 0.458 mmol, 79 %).

$C_{30}H_{28}N_2O$ (M = 480.56 g/mol). **^{1}H-NMR** ($CDCl_3$, 500 MHz, 25 °C): δ [ppm] = 7.87 (*s*, 2H, Ar*H*), 7.51 (*s*, 2H, Ar*H*), 7.10 (*dd*, *J* = 8.13 Hz, *J* = 1.98 Hz, 2H, Ar*H*), 7.04 (m, 4H, Ar*H*), 3.98 (s, 6H, C*H*3), 3.93 (s, 6H, C*H*3), 3.10 (s, 6H, C*H*3). **^{13}C-NMR** ($CDCl_3$, 125 MHz, 25 °C): δ [ppm] = 158.1, 149.8, 149.53, 130.3, 125.2, 124.7, 123.54, 122.5, 113.0, 111.4, 56.3, 56.2, 25.5. **DEPT 135-NMR** ($CDCl_3$, 125 MHz, 25 °C): δ [ppm] = 124.5, 123.2, 122.3, 112.8, 111.3, 56.1, 56.1, 25.3. **HRMS (ESI)** *m/z*: calcd. for $[M+H]^+$: 481.2049, found: 481.2126.

Synthesis of L1

The synthesis of the ligand **L1** was conducted according to a literature procedure.[24]

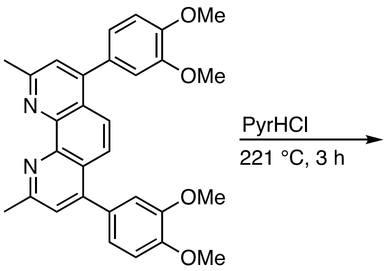

In a dried Schlenk flask equipped with a small magnetic stir bar, the methoxy derivative (200 mg, 0.420 mmol, 1 eq.) and pyridinium hydrochloride (12.1 g, 105 mmol, 250 eq.) were combined. The flask was connected to a reflux condenser and the whole apparatus was evacuated and purged with argon three times. The mixture was heated to 221 °C and refluxed for 3 hours, during which pyridinium hydrochloride undergoes a phase transition to the liquid state, resulting in a yellow solution. The reaction was allowed to cool down to 80 °C, after which 20 mL of water, followed by EtOH (20 mL) and additional water (50 mL) were added to the mixture. The mixture was stirred at 25 °C until a clear solution was achieved. This solution was neutralized with 1 M aq. NaOH-solution (10 mL) to pH 7, leading to the formation of red precipitates over time. After being left undisturbed overnight at 6 °C the solid was collected using a frit. The solid was washed thoroughly with water to remove excess pyridine and inorganic salts. The final purified product was dried in high vacuum (136 mg, 0.320 mmol, 76 %).

$C_{26}H_{20}N_2O$ (M = 424.46 g/mol). **^{1}H-NMR** ($(CD_3)_2SO$, 500 MHz, 25 °C): δ [ppm] = 9.27 (*s*, 2H, OH), 9.20 (*s*, 2H, OH), 7.83 (*s*, 2H, Ar*H*), 7.47 (*s*, 2H, Ar*H*), 6.92 (*m*, 4H, Ar*H*), 6.83 (*dd*, *J* = 8.09 Hz, *J* = 1.98 Hz, 2H, Ar*H*), 2.81 (s, 6H, C*H*$_3$). **^{13}C-NMR** ($(CD_3)_2SO$, 125 MHz, 25 °C): δ [ppm] = 157.6, 148.0, 145.9, 145.7, 145.4, 128.6, 124.0, 123.3, 122.6, 120.8, 117.0, 115.8, 25.0. **DEPT 135-NMR** ($(CD_3)_2SO$, 125 MHz, 25 °C): δ [ppm] = 123.0, 122.4, 120.6, 116.7, 115.6, 24.7. **HRMS (ESI)** *m/z*: calcd. for $[M+H]^+$: 425.1423, found: 425.1499.

Synthesis of C1'

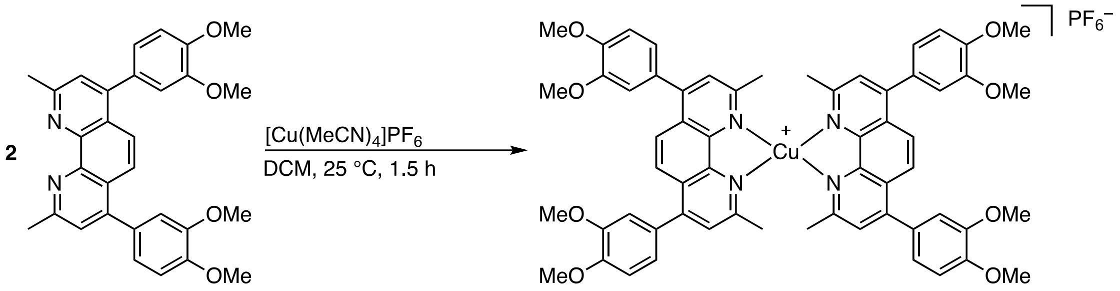

In a dried Schlenk flask equipped with a small magnetic stir bar, 4,7-Bis(3,4-dimethoxyphenyl)-2,9-dimethyl-1,10-phenanthroline (48 mg, 0.1 mmol, 2 eq.) and $[Cu(MeCN)_4]PF_6$ (18.6 mg, 0.05 mmol, 1 eq.) were added. The flask was connected to a reflux condenser and the whole apparatus was evacuated and purged with argon three times. Dry and degassed DCM (20 mL) was added *via* syringe and the reaction mixture was stirred for 1.5 h at 25 °C. Subsequently, *n*-hexane was added (*ca*. 20-30 ml). For purification, solution was first filtrated trough Celite since first precipitation appeared. Further, *n*-hexane was added (3-5 ml) for complete precipitation and mixture was stored overnight at +6 °C. After filtration and drying *in vacuo*, the product was obtained as a red solid (37 mg, 0.03 mmol, 62 %).

$C_{60}H_{56}CuF_6N_4O_8P_3$ (M = 1202.64 g/mol). **^{1}H-NMR** (MeCN-*d_3*, 500 MHz, 25 °C): δ [ppm] = 8.17 (*s*, 4H, Ar*H*), 7.84 (*s*, 4H, ArH), 7.23 (*m*, 8H, Ar*H*), 7.17 (*m*, 4H), 3.92 (*s*, 12H, C*H_3*), 3.89 (*s*, 12H, C*H_3*), 2.52 (*s*, 12H, C*H_3*). **^{13}C-NMR** (MeCN-*d_3*, 125 MHz, 25 °C): δ [ppm] = 158.4, 151.2, 150.5, 150.2, 144.9, 130.3, 126.7, 126.6, 124.7, 123.4, 114.3, 112.9, 56.6, 56.6, 26.1. **DEPT 135-NMR** (MeCN-*d_3*, 125 MHz, 25 °C): δ [ppm] = 126.6, 124.6, 123.3, 114.2, 112.7, 56.5, 56.4, 26.0. **HRMS (ESI)** *m/z*: calcd. $[M-PF_6]^+$: 1023.3389, found: 1023.3399.

Synthesis of C1

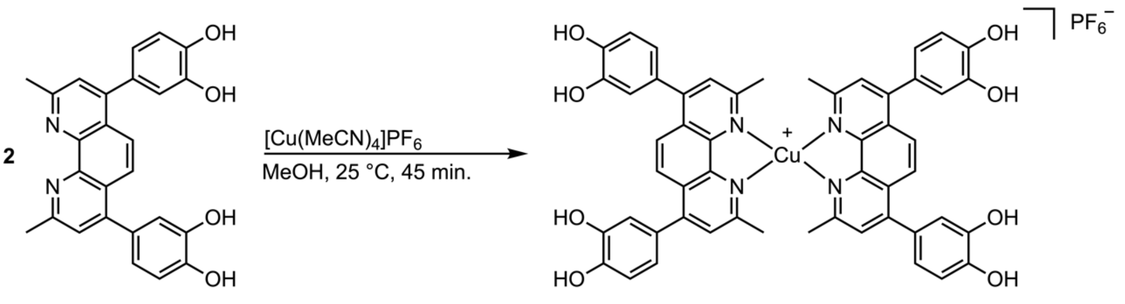

A dry Schlenk flask equipped with a small magnetic stir bar was charged with 4,7-Bis(3,4-dihydroxyphenyl)-2,9-dimethyl-1,10-phenanthroline (48 mg, 0.1 mmol, 2 eq.) and $[Cu(MeCN)_4]PF_6$ (18.6 mg, 0.05 mmol, 1 eq.). The whole apparatus was evacuated and purged with argon three times. Dry and degassed MeOH (15 mL) was added *via* syringe. The mixture was stirred for 45 min. at 25 °C. Subsequently, reaction mixture was constricted and purified by size exclusion chromatography (Sephadex® LH-20, Methanol/DCM (1:1)). After drying *in vacuo*, the final product was obtained as a dark red solid. (47 mg, 0.04 mmol, 74%).

$C_{52}H_{40}CuF_6N_4O_8P$ (M = 1057.42 g/mol). **^{1}H-NMR** (DMSO-d_6, 300 MHz, 25 °C): δ [ppm] = 9.50 (br *s*, 4H, O*H*), 9.36 (br *s*, 4H, O*H*), 8.18 (*s*, 4H, ArH), 7.86 (*s*, 4H, Ar*H*), 7.11 (*s*, 4H, Ar*H*), 7.00 (s, 8H, Ar*H*), 2.48 (*s*, 12H, C*H*$_3$). **^{13}C-NMR** (MeCN-d_3, 125 MHz, 25 °C): δ [ppm] = 156.9, 148.9, 146.7, 145.7, 143.3, 127.4, 125.3, 124.8, 123.6, 121.2, 117.0, 116.0, 25.5. **DEPT 135-NMR** (MeCN-d_3, 125 MHz, 25 °C): δ [ppm] = 125.0, 123.3, 121.0, 116.8, 115.8, 25.2. **HRMS (ESI)** *m/z*: calcd. $[M-PF_6]^+$: 911.2137, found: 911.2145.

Synthesis of C2'

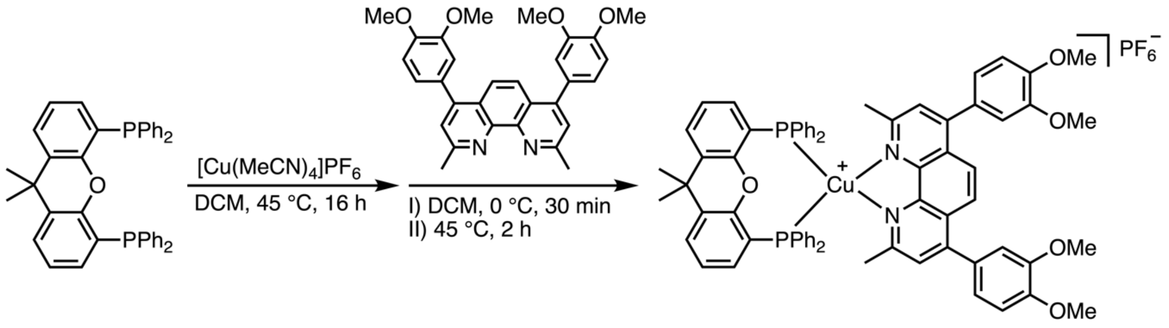

A dried Schlenk tube was charged with Xantphos (58.9 mg, 0.1 mmol, 1 eq.) and $[Cu(MeCN)_4]PF_6$ (37.3 mg, 0.1 mmol, 1 eq.) and dissolved with dry and degassed DCM (15 mL). The solution was heated to 45 °C and stirred for 16 h. Subsequently, the solution was first cooled to 25 °C followed by cooling to 0 °C using an ice bath. A solution of 7-Bis(3,4-dimethoxyphenyl)-2,9-dimethyl-1,10-phenanthroline (48.1 mg, 0.1 mmol, 1 eq.) in dry and degassed DCM (15 mL) was slowly added dropwise at 0 °C using a syringe pump (*ca.* 8-10 mL/h) (**I**). After complete addition, stirring was continued for 30 min at 0 °C followed by 2 h at 45 °C (**II**). Subsequently, the heteroleptic target complex was precipitated with *n*-hexane and was cleared by filtration through Celite. For complete precipitation 3-5 ml of n-hexane was added and solution was stored at + 6 °C overnight. After filtration and drying *in vacuo*, the final complex remained as a yellow solid (73 mg, 0.058 mmol, 58 %).

$C_{69}H_{60}CuF_6N_2O_5P_3$ (M = 1267.71 g/mol). **^{1}H-NMR** (MeCN-d_3, 500 MHz, 25 °C): δ [ppm] = 7.86 (*s*, 2H, Ar*H*), 7.77 (*dd*, *J* = 7.8 Hz, *J* = 1.3 Hz, 2H, Ar*H*), 7.52 (*s*, 2H, Ar*H*), 7.27 (*m*, 6H, Ar*H*), 7.11 (*m*, 22H, Ar*H*), 7.03 (*m*, 2H. Ar*H*), 3.90 (*s*, 6H, CH_3), 3.87 (*s*, 6H, CH_3), 2.31 (*s*, 6H, CH_3), 1.73 (*s*, 6H, CH_3). **^{13}C-NMR** (MeCN-d_3, 125 MHz, 25 °C): δ [ppm] = 158.9, 155.9, 151.2, 150.6, 150.4, 144.7, 134.9, 134.0, 132.6, 131.2, 131.0, 130.1, 129.6, 128.9, 126.6, 126.4, 124.3, 123.4, 122.7, 114.2, 112.8, 56.6, 37.0, 28.9, 27.9. **DEPT 135-NMR** (MeCN-d_3, 125 MHz, 25 °C): δ [ppm] = 133.9, 131.1, 130.8, 129.4, 128.8, 126.3, 126.2, 124.2, 123.2, 114.1, 112.6, 56.5, 56.4, 28.7, 27.7. **^{31}P-NMR** (MeCN-d_3, 202 MHz, 25 °C): δ [ppm] = -12.95 (*sept*, PF_6). **HRMS (ESI)** *m/z*: calcd. for $[M\text{-}PF_6]^+$: 1121.3268, found: 1121.3277.

Synthesis of C2

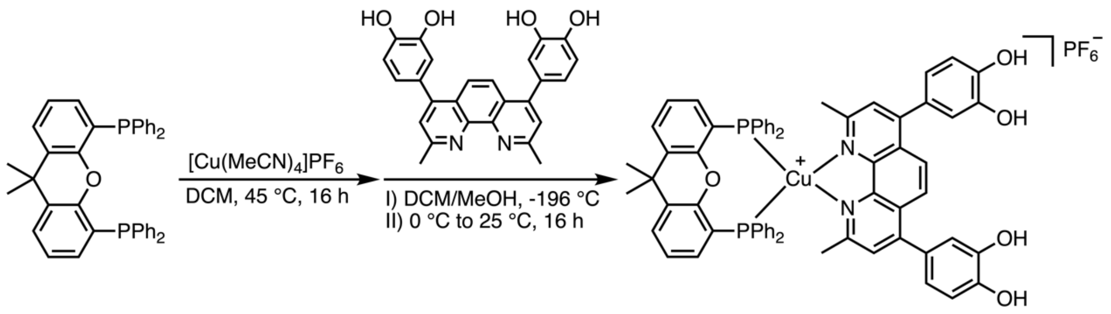

In a dried Schlenk flask Xantphos (139 mg, 0.240 mmol, 1 eq.), $[Cu(MeCN)_4]PF_6$ (88 mg, 0.236 mmol, 1 eq.) were submitted and dry and degassed DCM (20 mL) were added *via* syringe. The reaction mixture was stirred for 16 hours at 45 °C. Subsequently, the solution was first cooled to 25 °C and then dry and degassed Methanol (MeOH) (20 mL) were added *via* syringe. The magnetic stir bar was moved to the top side of the flask with an outsider magnet and the reaction mixture was frozen with a liquid nitrogen bath (**I**). 4,4'-(2,9-dimethyl-1,10-phenanthroline-4,7-diyl)bis(benzene-1,2-diol) (100 mg, 0.236 mmol, 1 eq.) was added as a solid on top of the frozen DCM/MeOH solvent mixture. The mixture was allowed to warm to 25 °C very slowly, first using an NaCl-ice bath at -20 °C (**II**). The solution was stirred for 16 hours at 25 °C. Subsequently, the reaction solution was constricted and purified *via* Sephadex® (DCM/MeOH(1:1). After solvent removal and drying *in vacuo*, the final complex was obtained (168 mg, 0.139 mmol, 59 %).

$C_{65}H_{52}CuF_6N_2O_5P_3$ (M = 1211.60 g/mol)l.**^{1}H-NMR** (MeCN-d_3, 500 MHz, 25 °C): δ [ppm] = 7.83 (*s*, 2H, Ar*H*), 7.76 (*dd*, *J* = 7.75 Hz, *J* = 1.23 Hz, 2H, Ar*H*), 7.45 (*s*, 2H, Ar*H*), 7.27 (*m*, 6H, Ar*H*), 7.08 (*m*, 22H, Ar*H*), 6.91 (*d*, *J* = 8.21 Hz, 2H, Ar*H*), 2.28 (*s*, 6H, C*H*$_3$), 1.72 (*s*, 6H, C*H*$_3$). **^{13}C-NMR** (MeCN-d_3, 125 MHz, 25 °C): δ [ppm] 155.9, 150.7, 146.7, 145.8, 144.6, 134.9, 134.0, 132.6, 131.2, 130.9, 129.8, 129.6, 128.9, 126.6, 126.4, 126.2, 124.3, 123.0, 122.7, 117.7, 116.6, 37.0, 28.9, 27.9. **DEPT 135-NMR** (MeCN-d_3, 125 MHz, 25 °C): δ [ppm] = 133.8, 131.1, 130.8, 129.4, 128.7, 126.2, 126.1, 124.1, 122.8, 117.r5, 116.5, 28.7, 27.7. **^{31}P-NMR** (MeCN-d_3, 202 MHz, 25 °C): δ [ppm] = -13.22 (*sept*, PF_6). **HRMS (ESI)** *m/z*: calcd. for $[M-PF_6]^+$: 1065.2642, found: 1065.2652.

3 NMR spectra

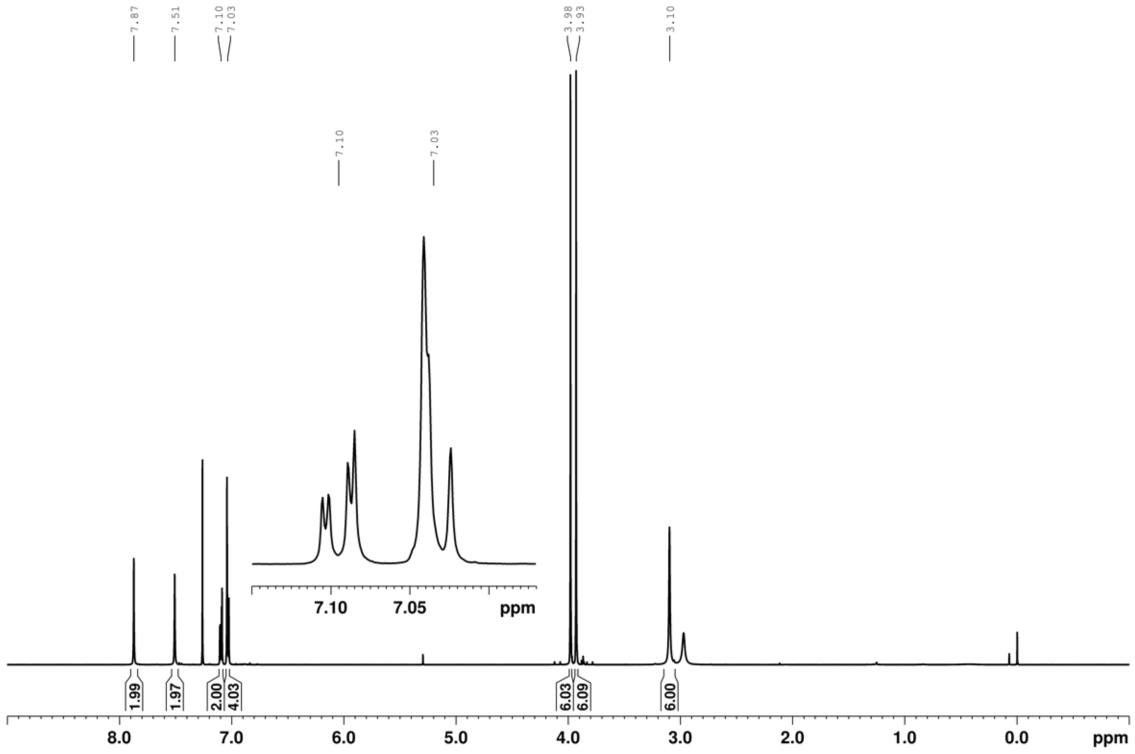

Figure S3.1. ^{1}H-NMR spectrum of **L1'** ($CDCl_3$, 500 MHz).

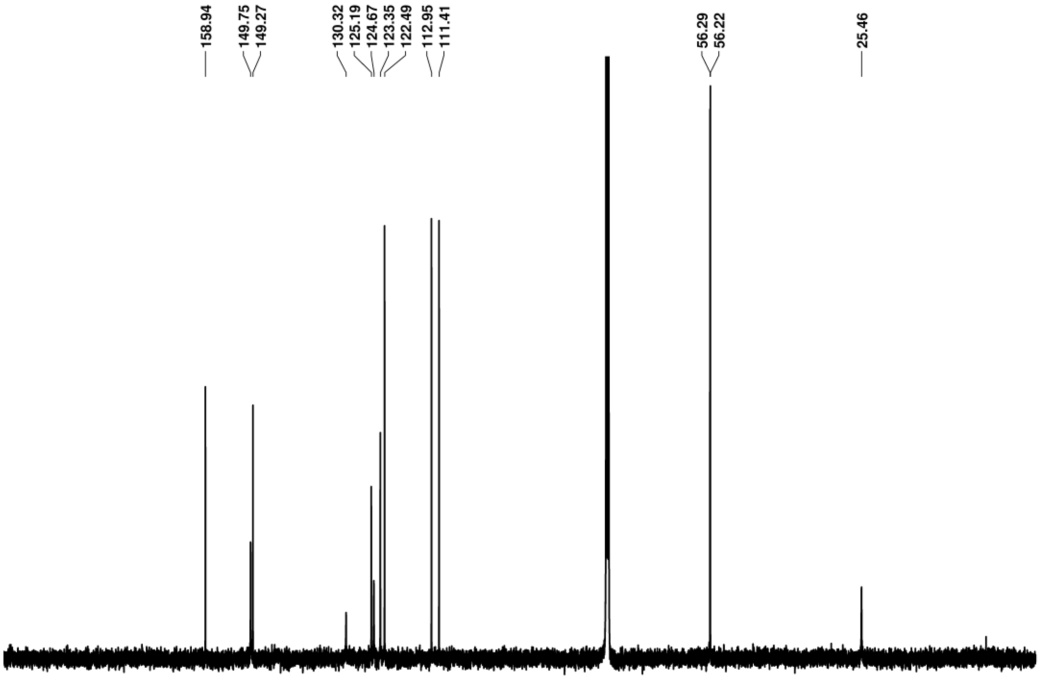

Figure S3.2. $^{13}C\{^{1}H\}$-NMR spectrum of **L1'** ($CDCl_3$, 125 MHz).

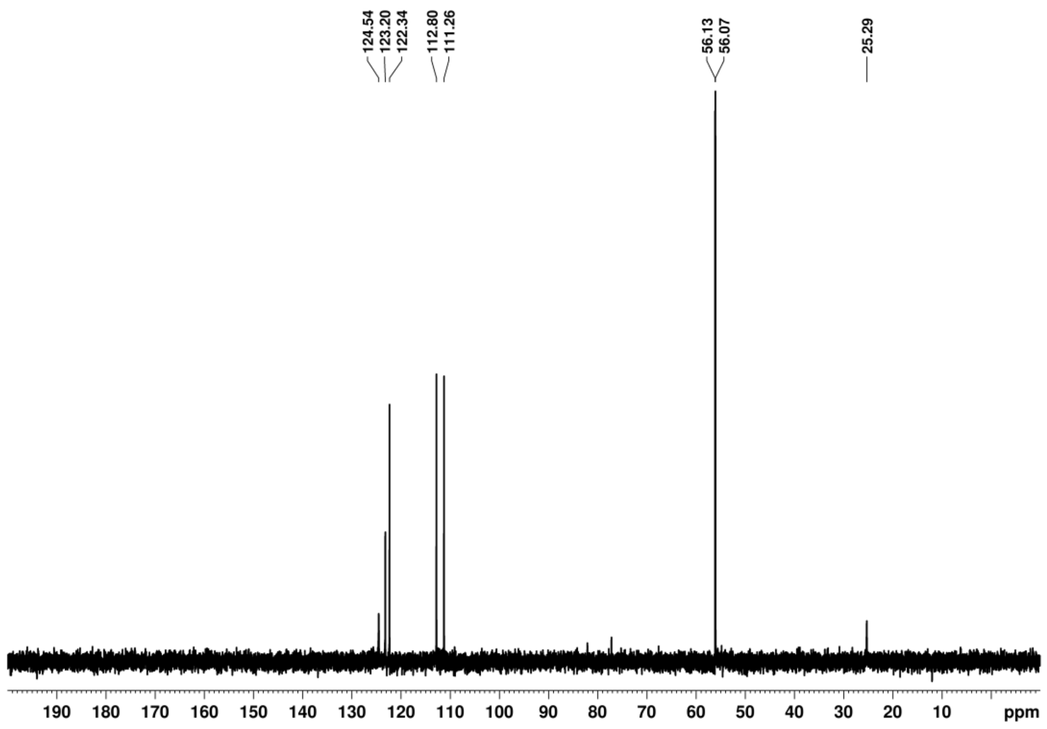

Figure S3.3. DEPT 135{^{1}H}-NMR of **L1'** ($CDCl_3$, 125 MHz).

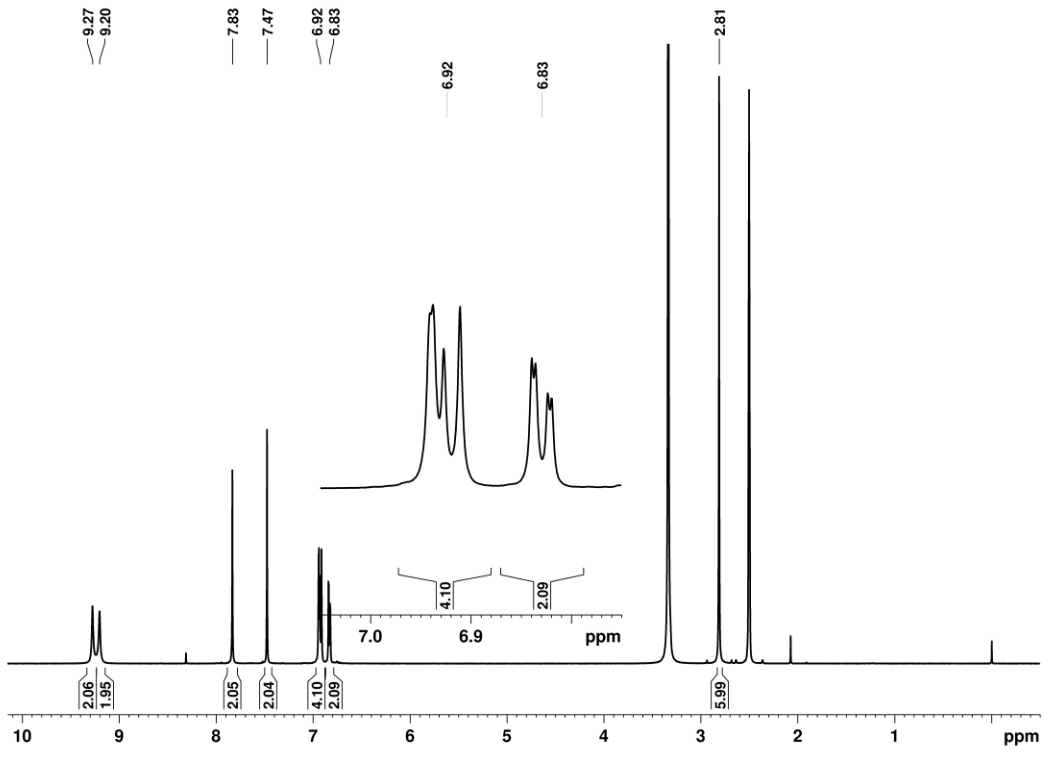

Figure S3.4. ^{1}H-NMR spectrum of **L1** ($(CD_3)_2SO$, 500 MHz).

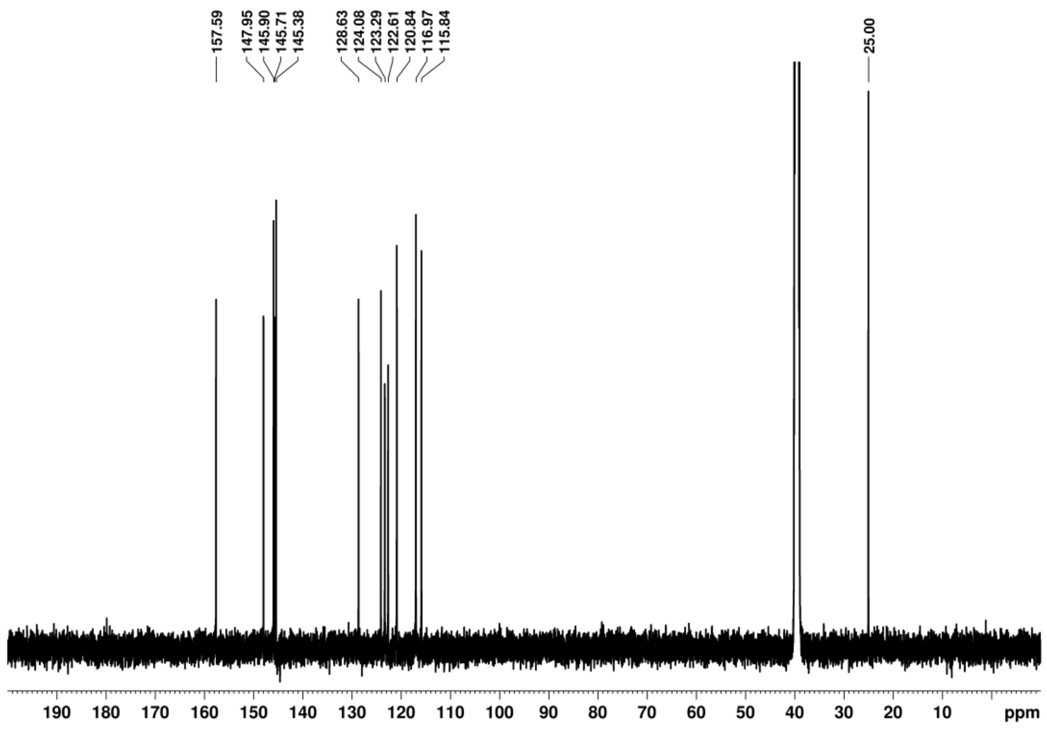

Figure S3.5. $^{13}C\{^1H\}$-NMR spectrum of **L1** ($(CD_3)_2SO$, 125 MHz).

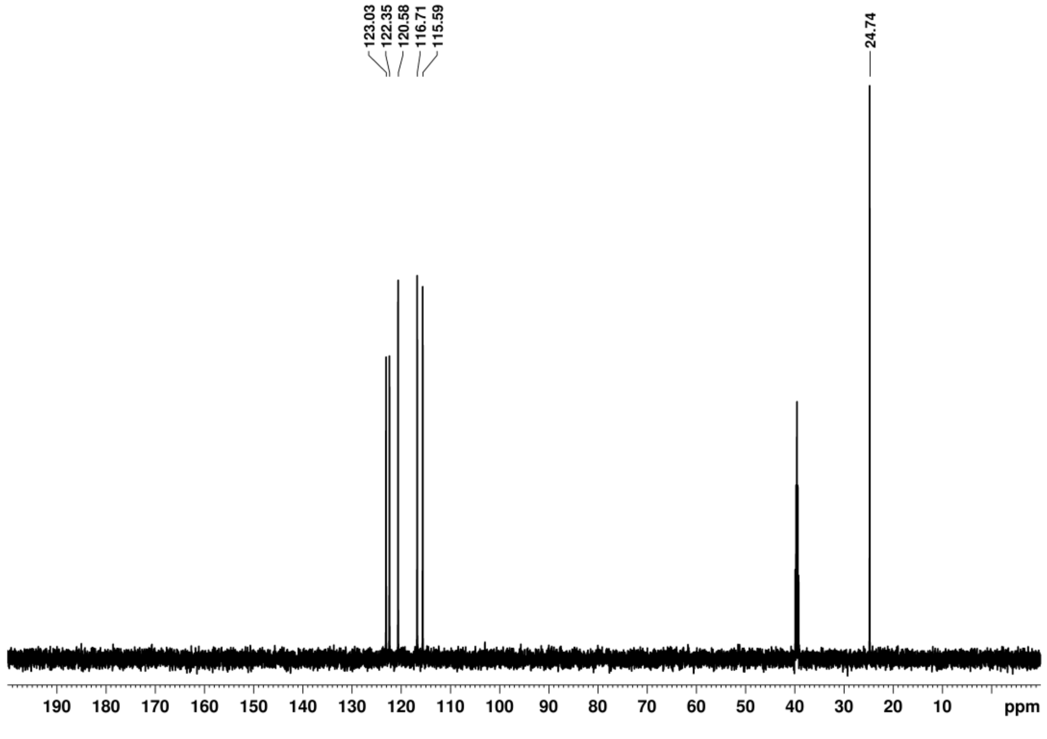

Figure S3.6. DEPT 135$\{^1H\}$-NMR spectrum of **L1** ($(CD_3)_2SO$, 125 MHz).

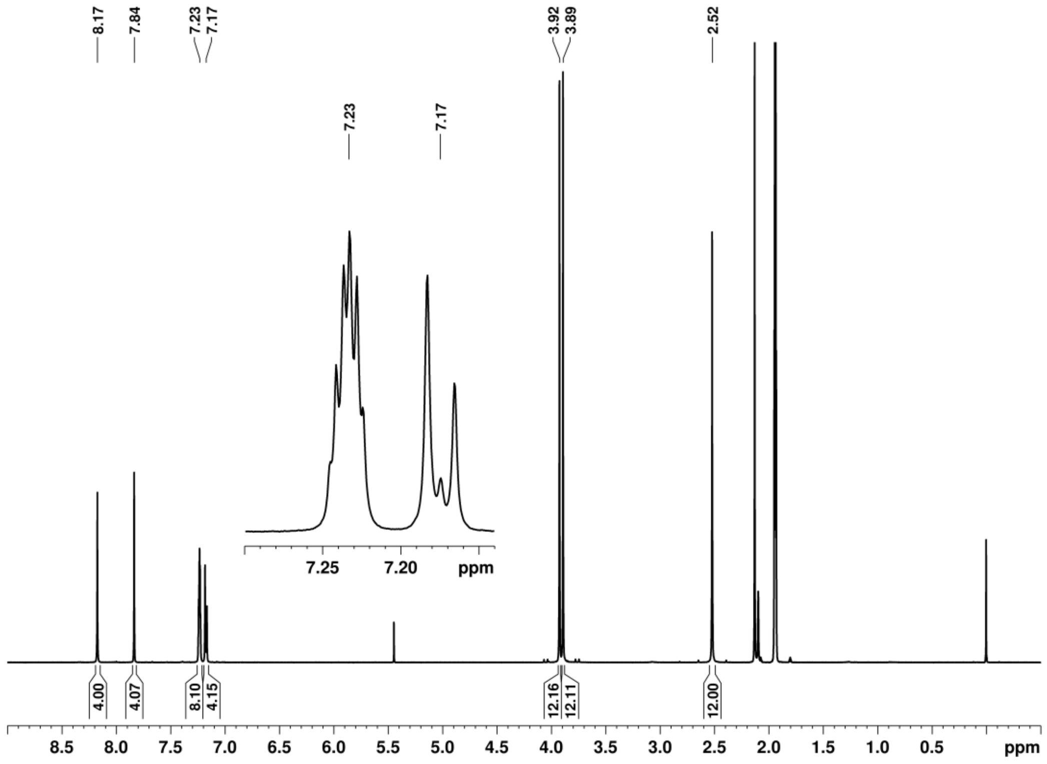

Figure S3.7. ^{1}H-NMR spectrum of **C1'** (CD_3CN. 500 MHz).

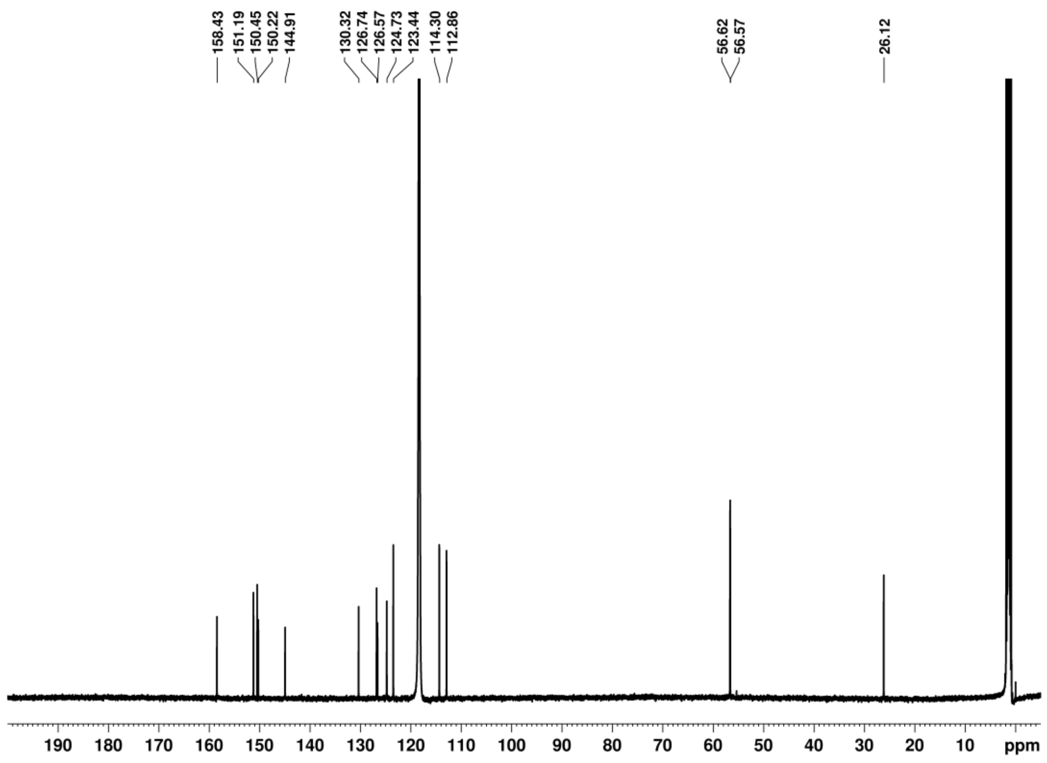

Figure S3.8. $^{13}C\{^{1}H\}$-NMR spectrum of **C1'** (CD_3CN, 125 MHz).

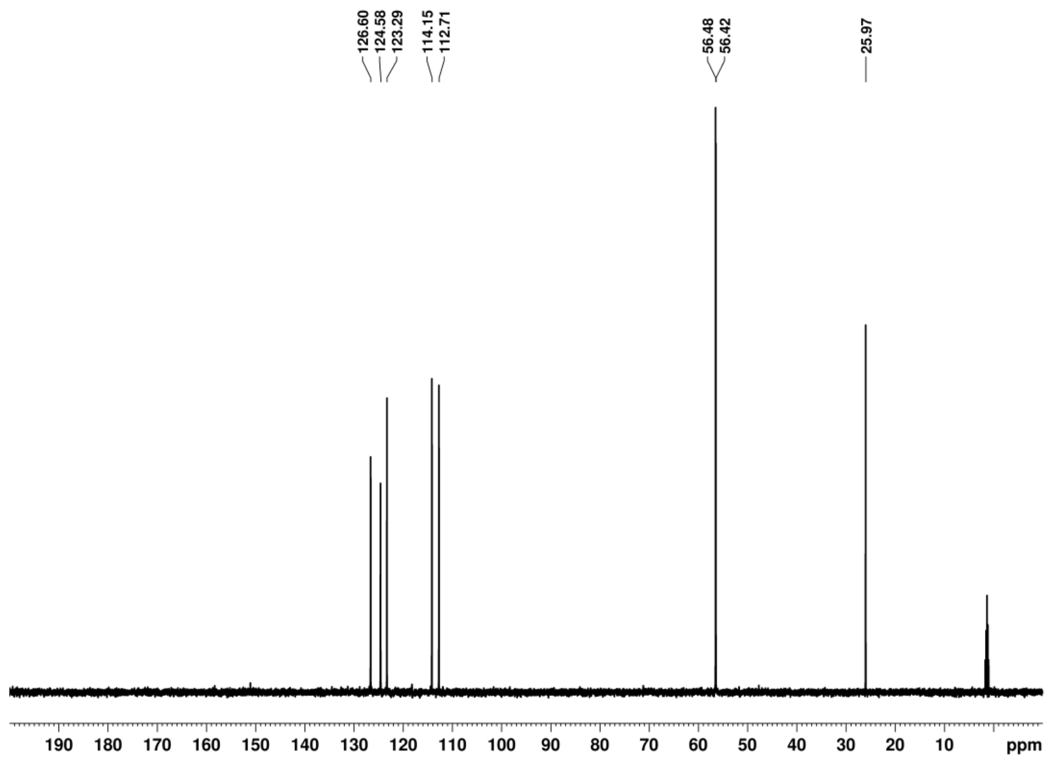

Figure S3.9. DEPT 135{^{1}H}-NMR spectrum of **C1'** (CD_3CN, 125 MHz).

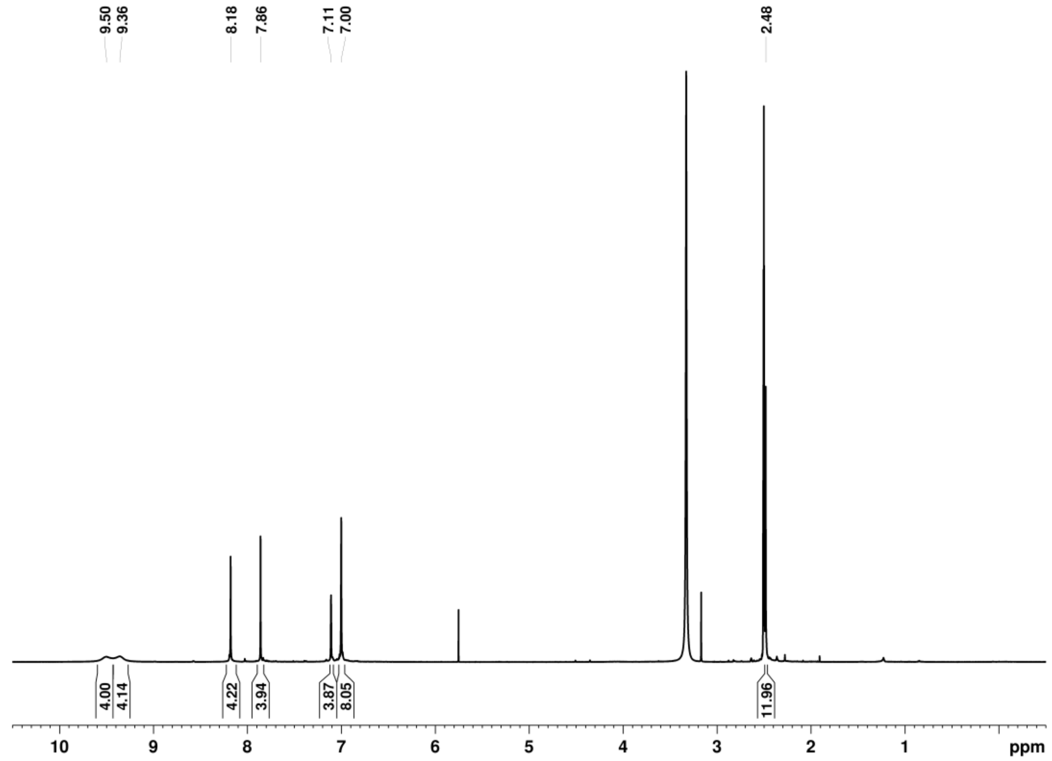

Figure S3.10. ^{1}H-NMR spectrum of **C1** ($(CD_3)_2SO$, 500 MHz).

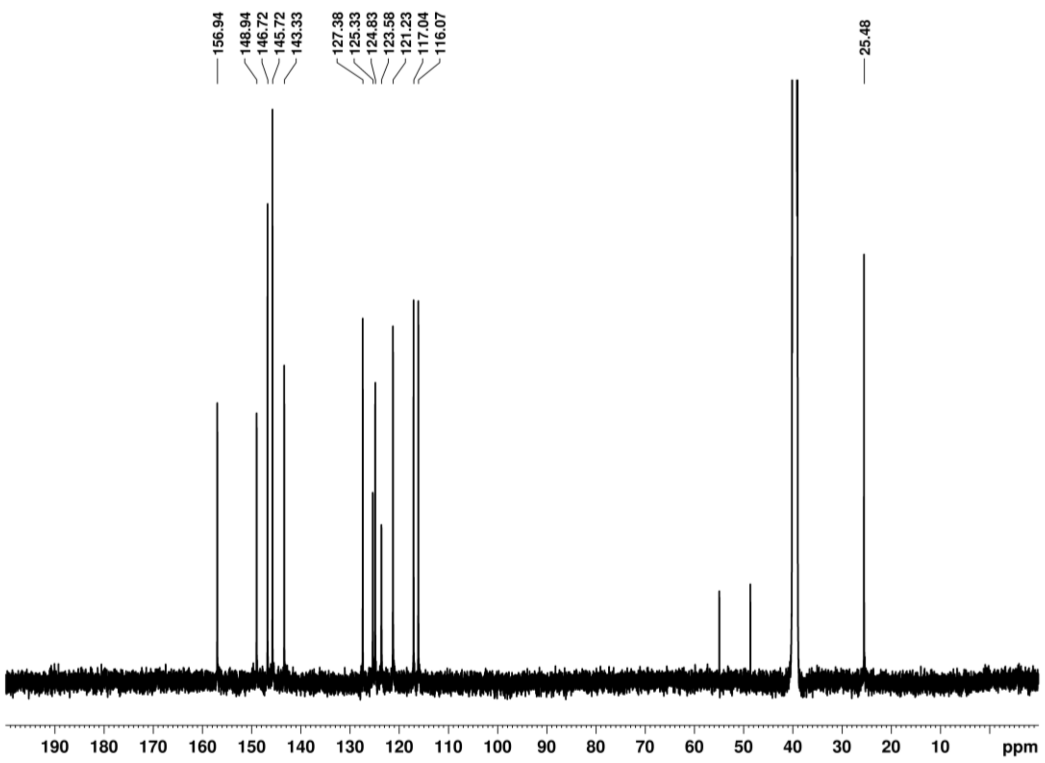

Figure S3.11. $^{13}C\{^{1}H\}$-NMR spectrum of **C1** ($(CD_3)_2SO$, 125 MHz).

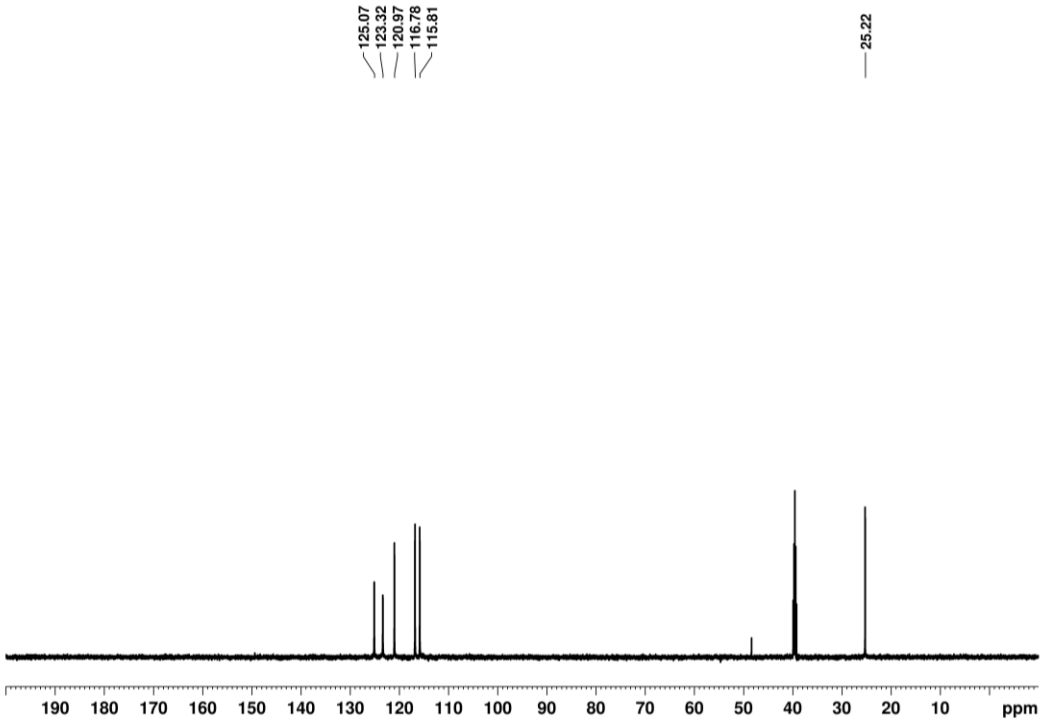

Figure S3.12. DEPT 135$\{^{1}H\}$-NMR spectrum of **C1** ($(CD_3)_2SO$, 125 MHz).

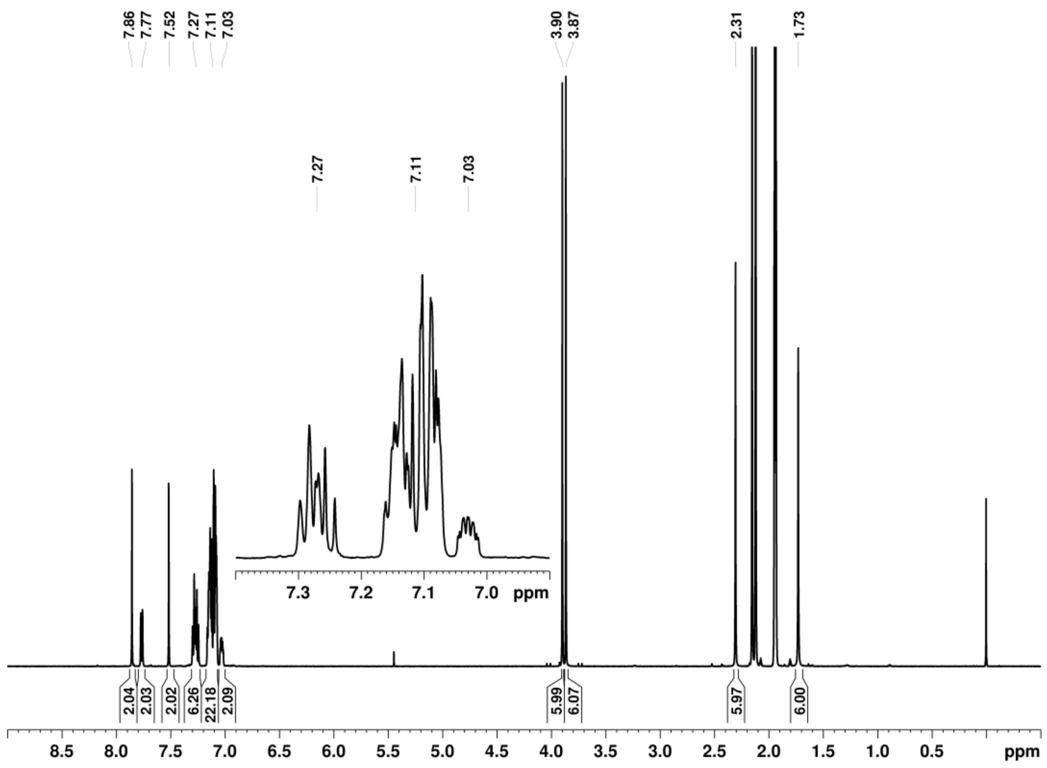

Figure S3.13. ^{1}H-NMR spectrum of **C2'** (CD_3CN, 500 MHz).

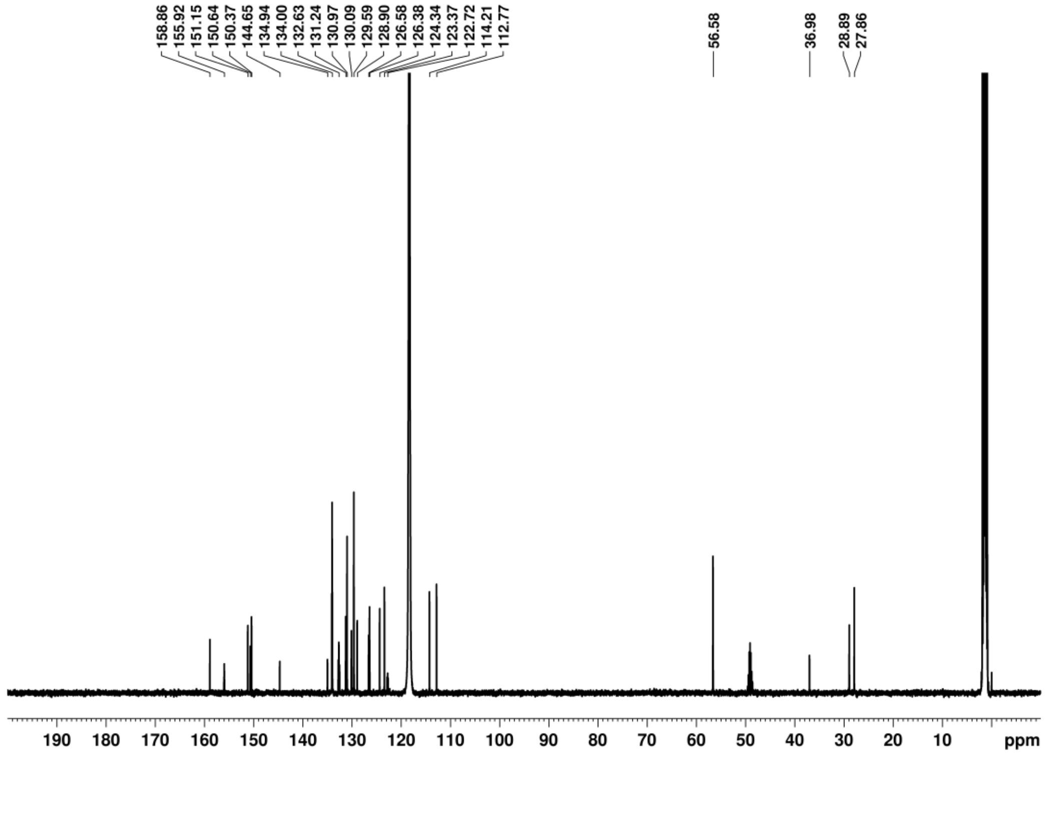

Figure S3.14. $^{13}C\{^{1}H\}$-NMR spectrum of **C2'** (CD_3CN, 125 MHz).

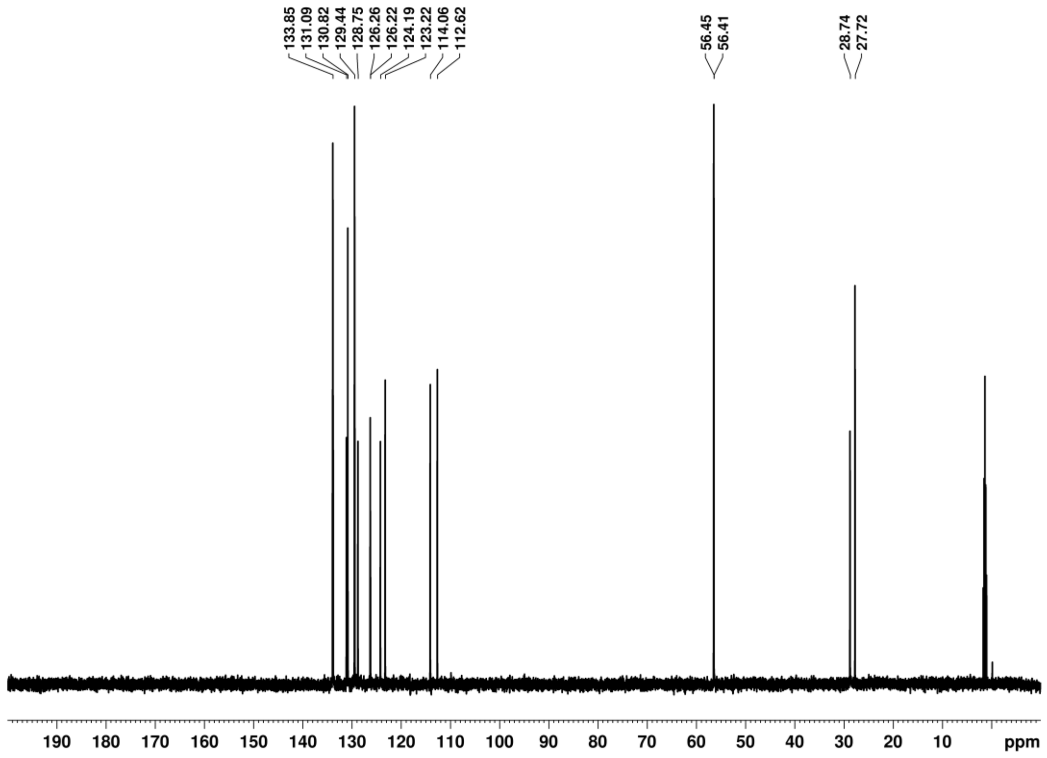

Figure S3.15. DEPT 135{^{1}H}-NMR spectrum of **C2'** (CD_3CN, 125 MHz).

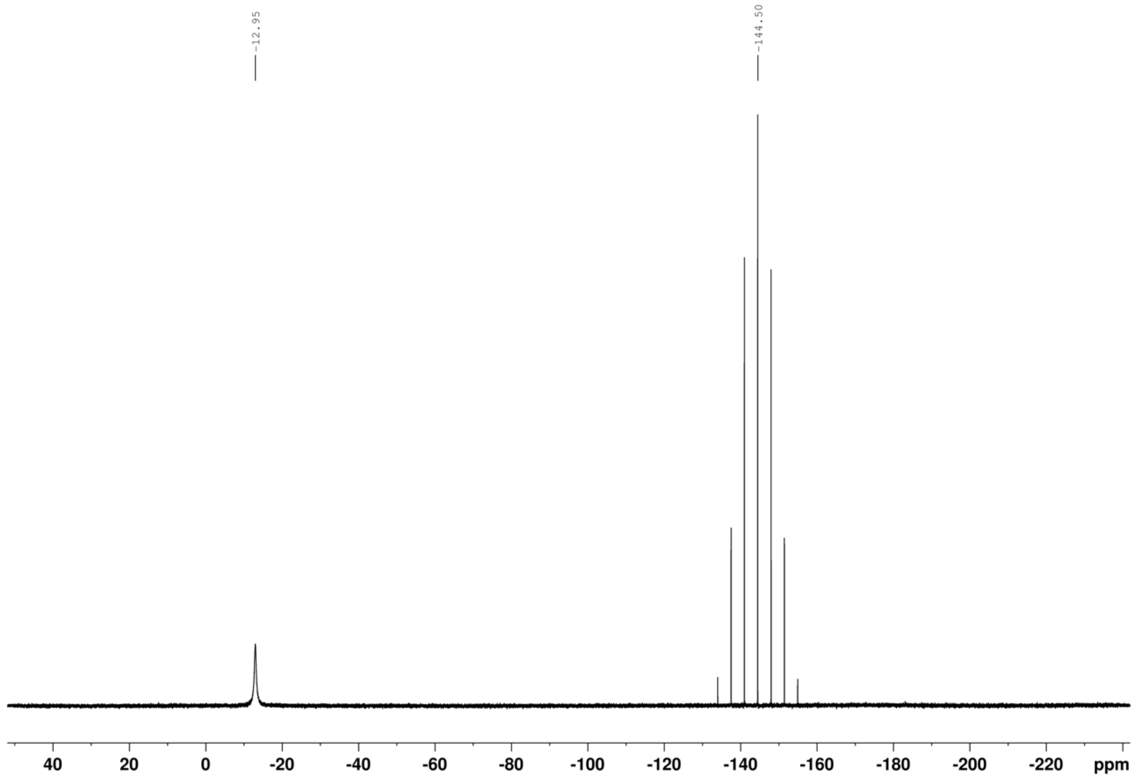

Figure S3.16. ^{31}P{^{1}H}-NMR spectrum of **C2'** (CD_3CN, 202 MHz).

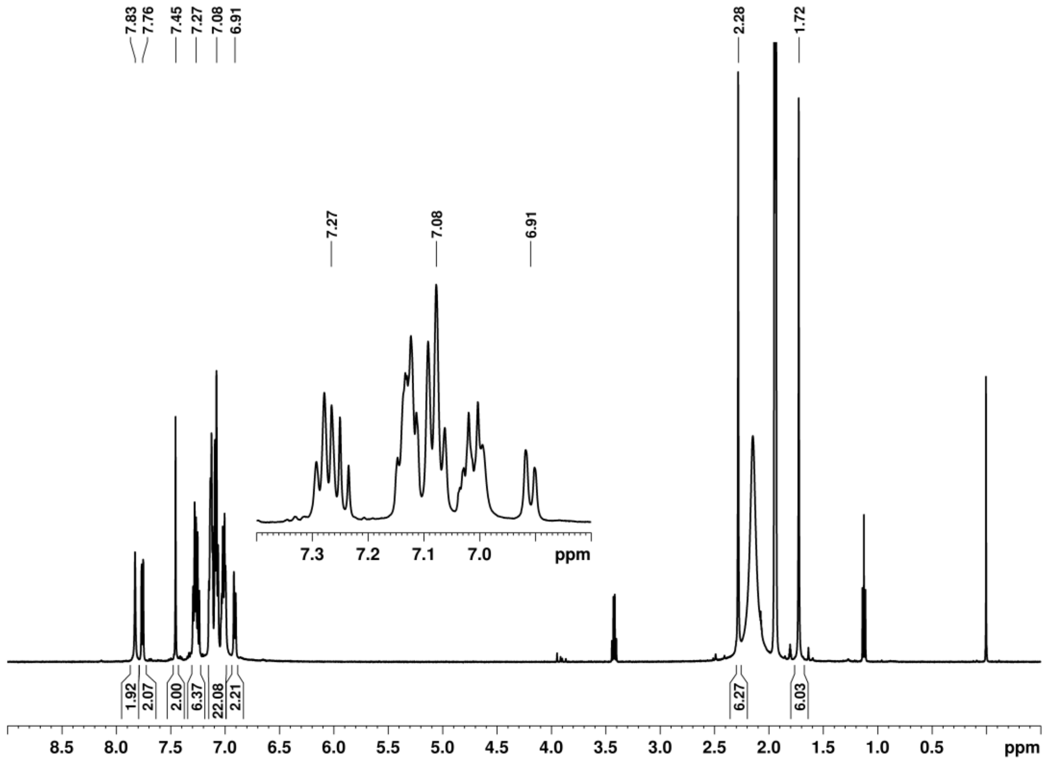

Figure S3.17. ^{1}H-NMR spectrum of **C2** (CD_3CN, 500 MHz).

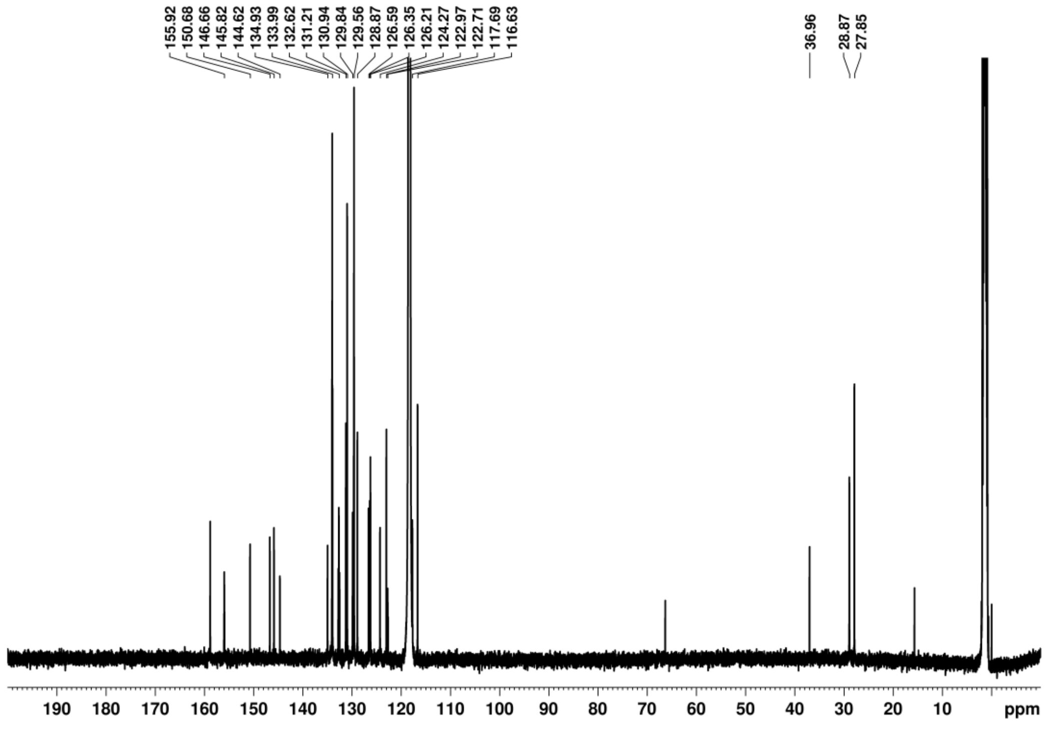

Figure S3.18. $^{13}C\{^{1}H\}$-NMR spectrum of **C2** (CD_3CN, 125 MHz).

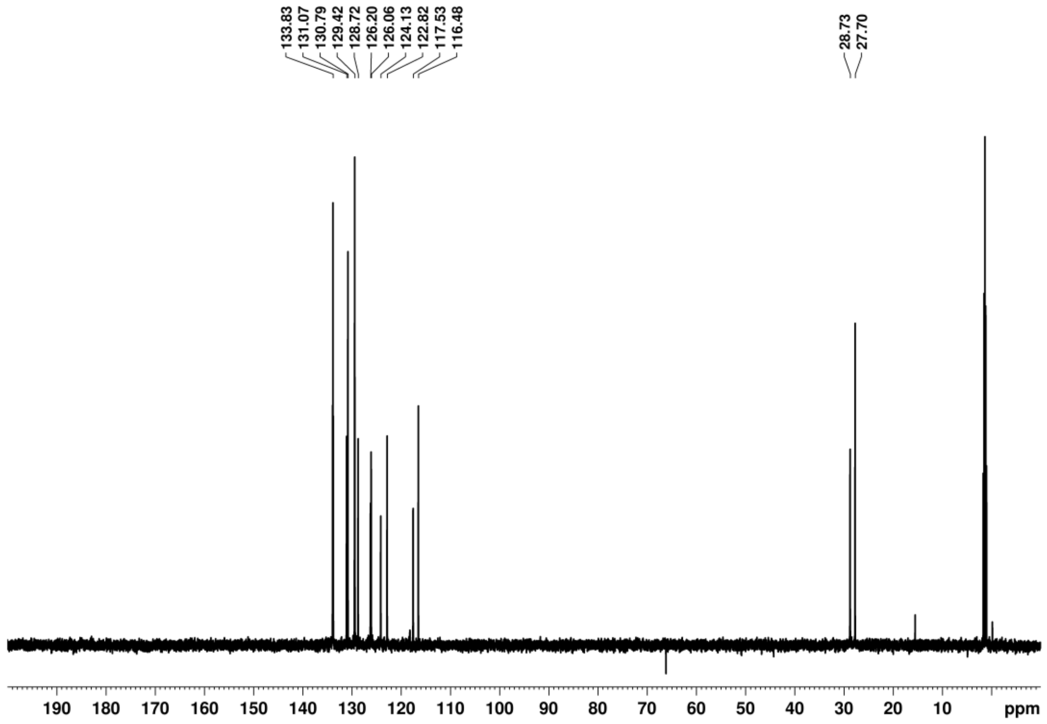

Figure S3.19. DEPT 135{^{1}H}-NMR spectrum of **C2** (CD_3CN, 125 MHz).

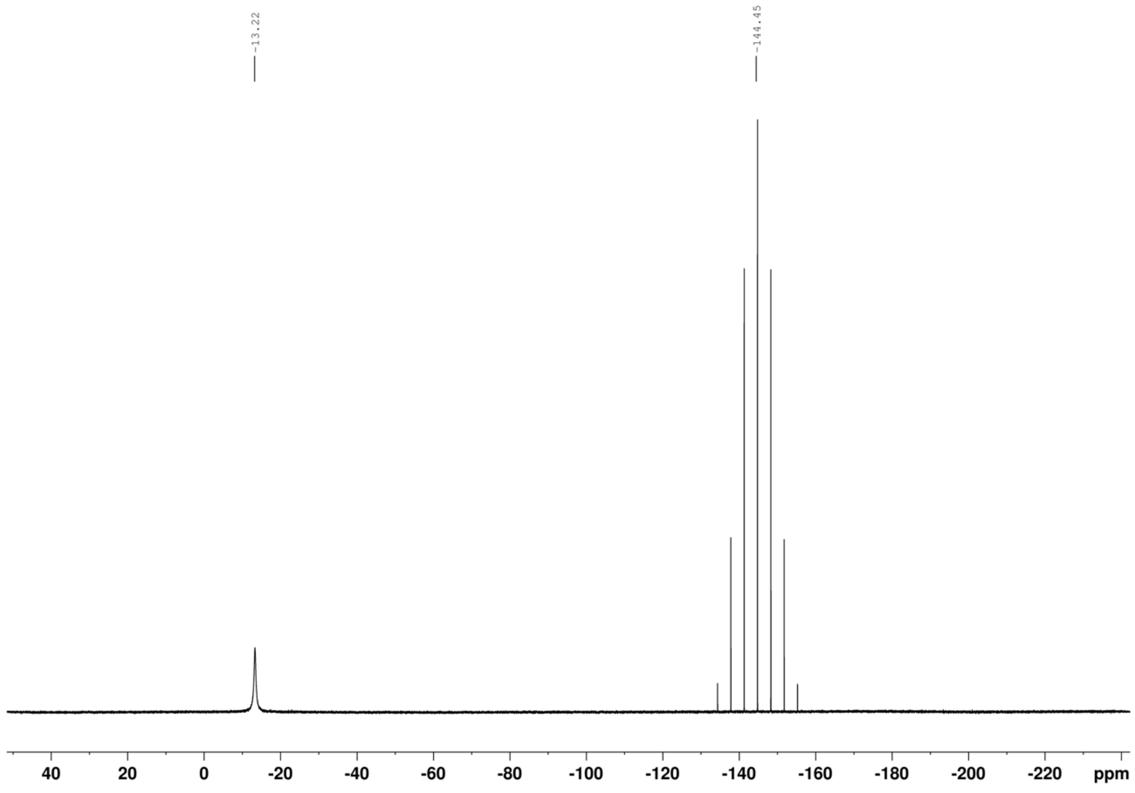

Figure S3.20. ^{31}P{^{1}H}-NMR spectrum of **C2** (CD_3CN, 202 MHz).

4 MS spectra

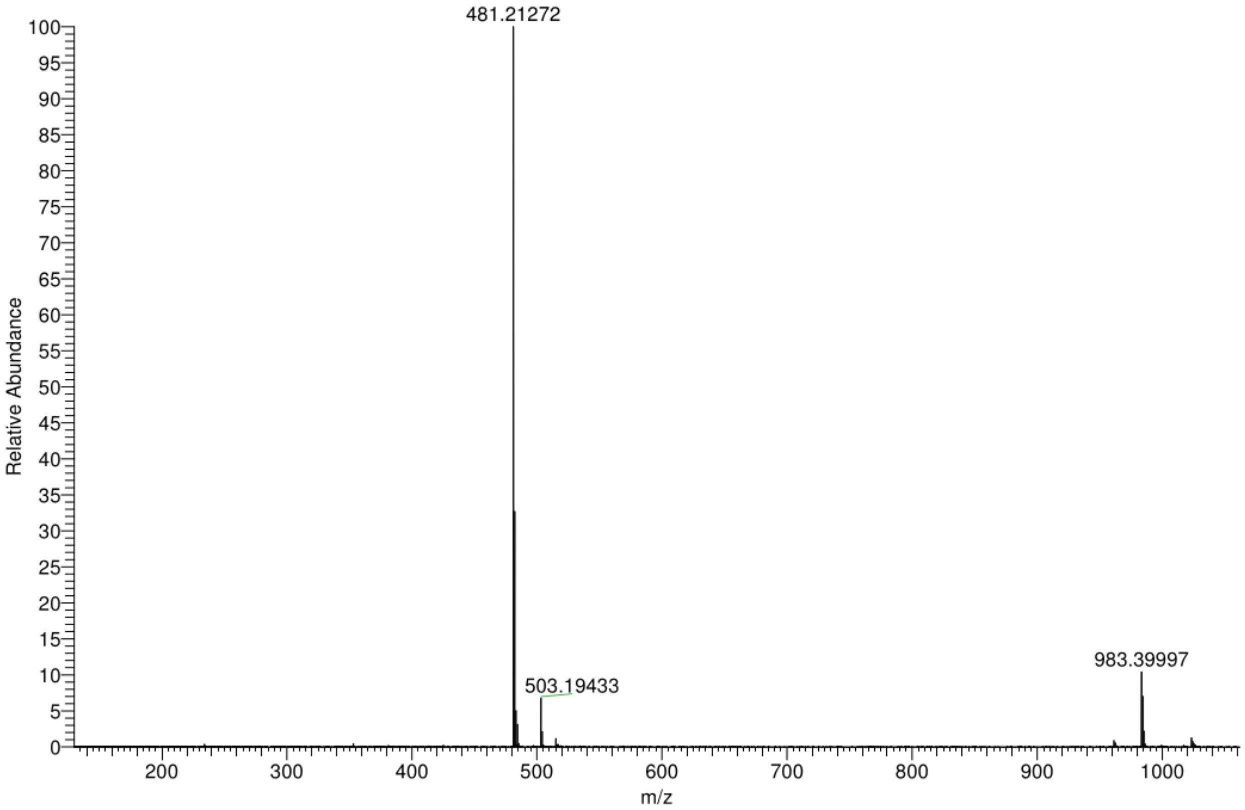

Figure S4.1. HRMS (ESI) of **L1'** in MeOH (fullscan).

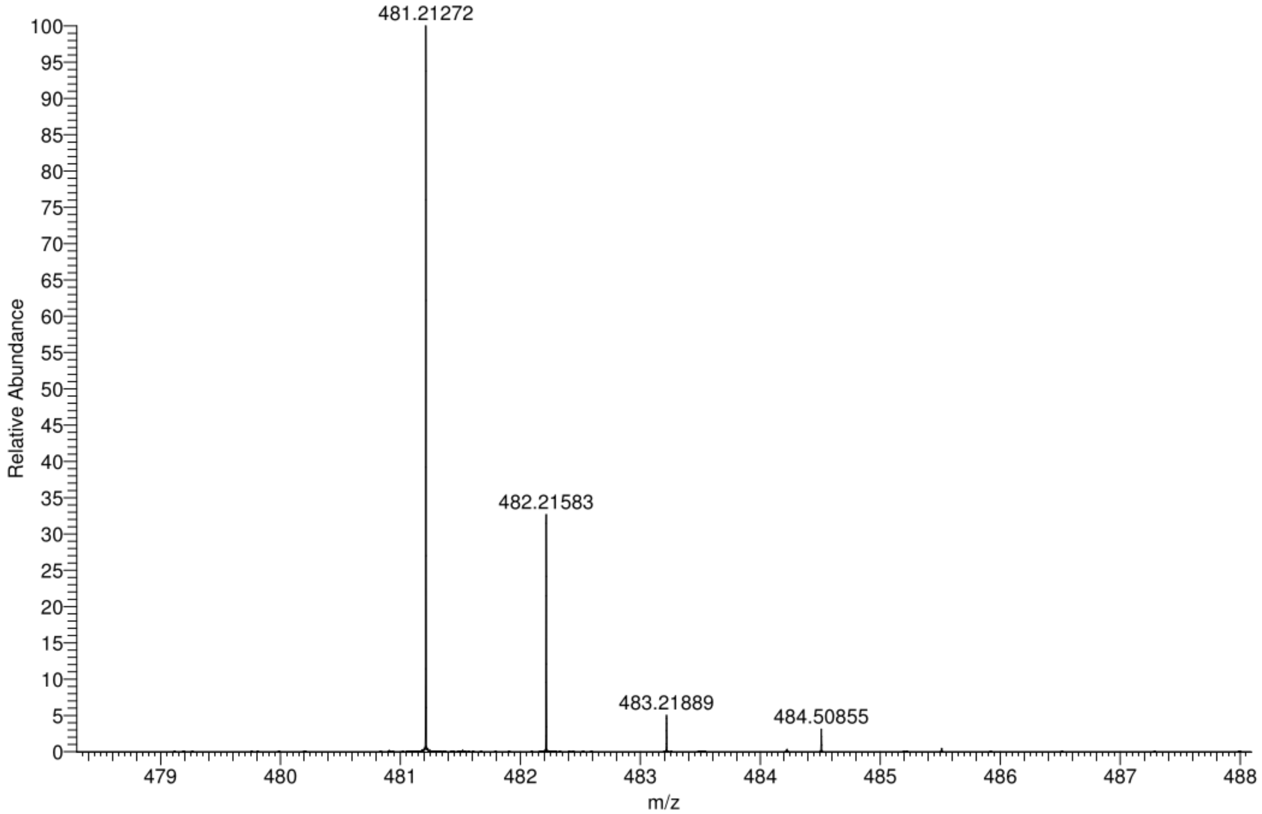

Figure S4.2. HRMS (ESI) of **L1'** in MeOH (zoom).

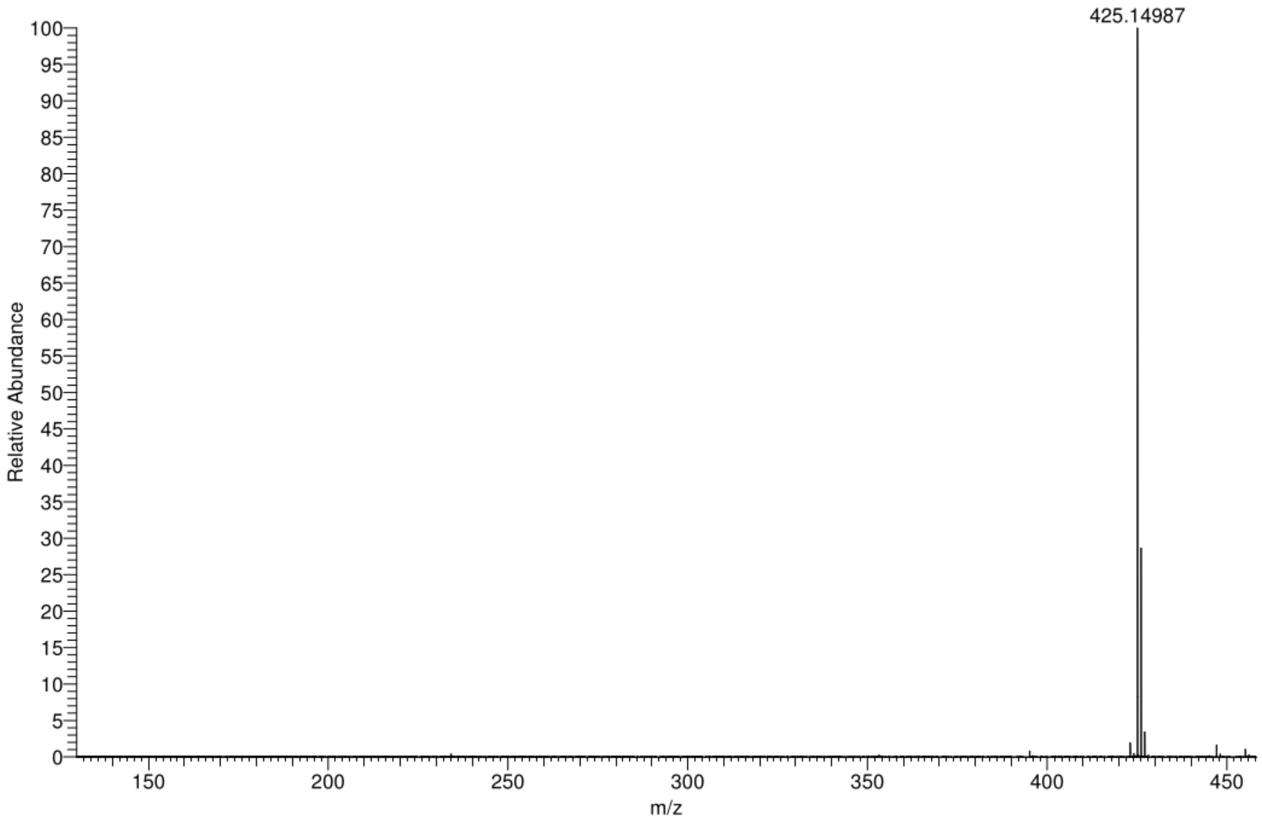

Figure S4.3. HRMS (ESI) of **L1** in MeOH (fullscan).

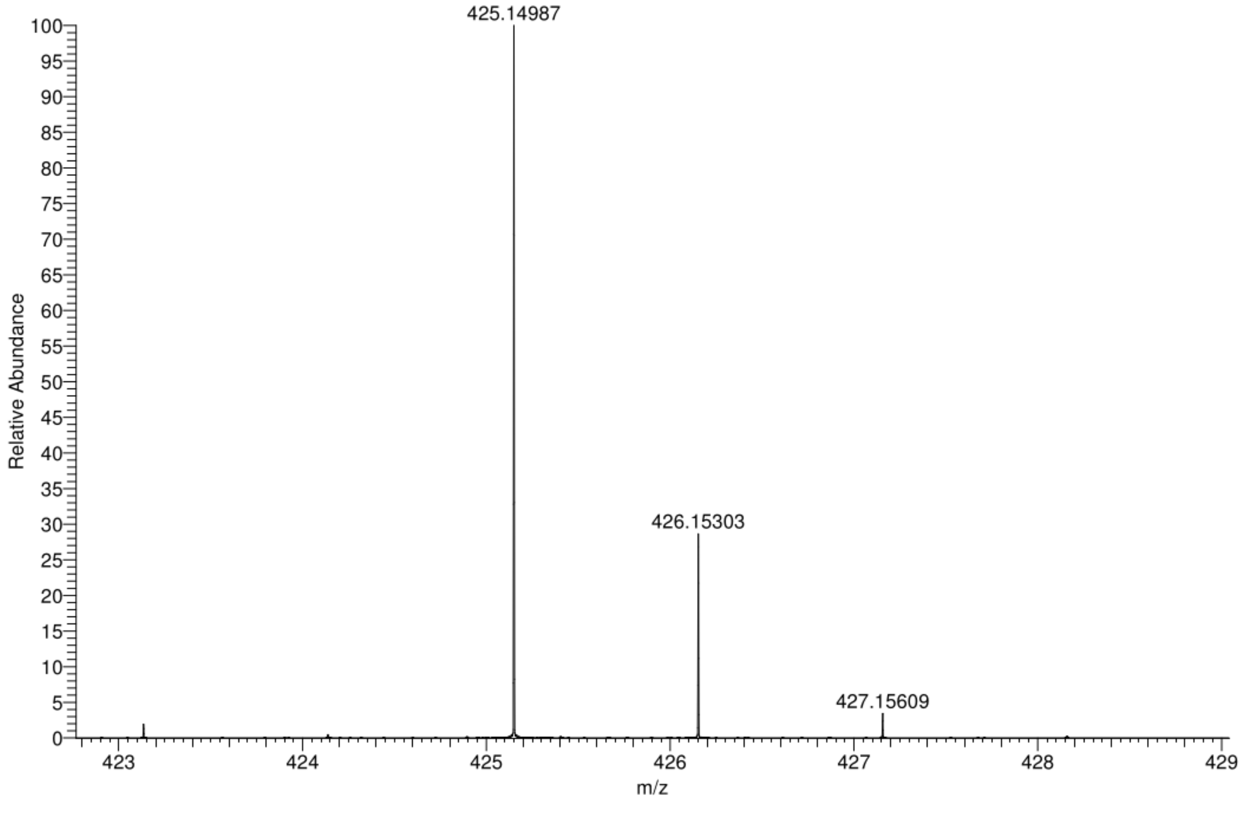

Figure S4.4. HRMS (ESI) of **L1** in MeOH (zoom).

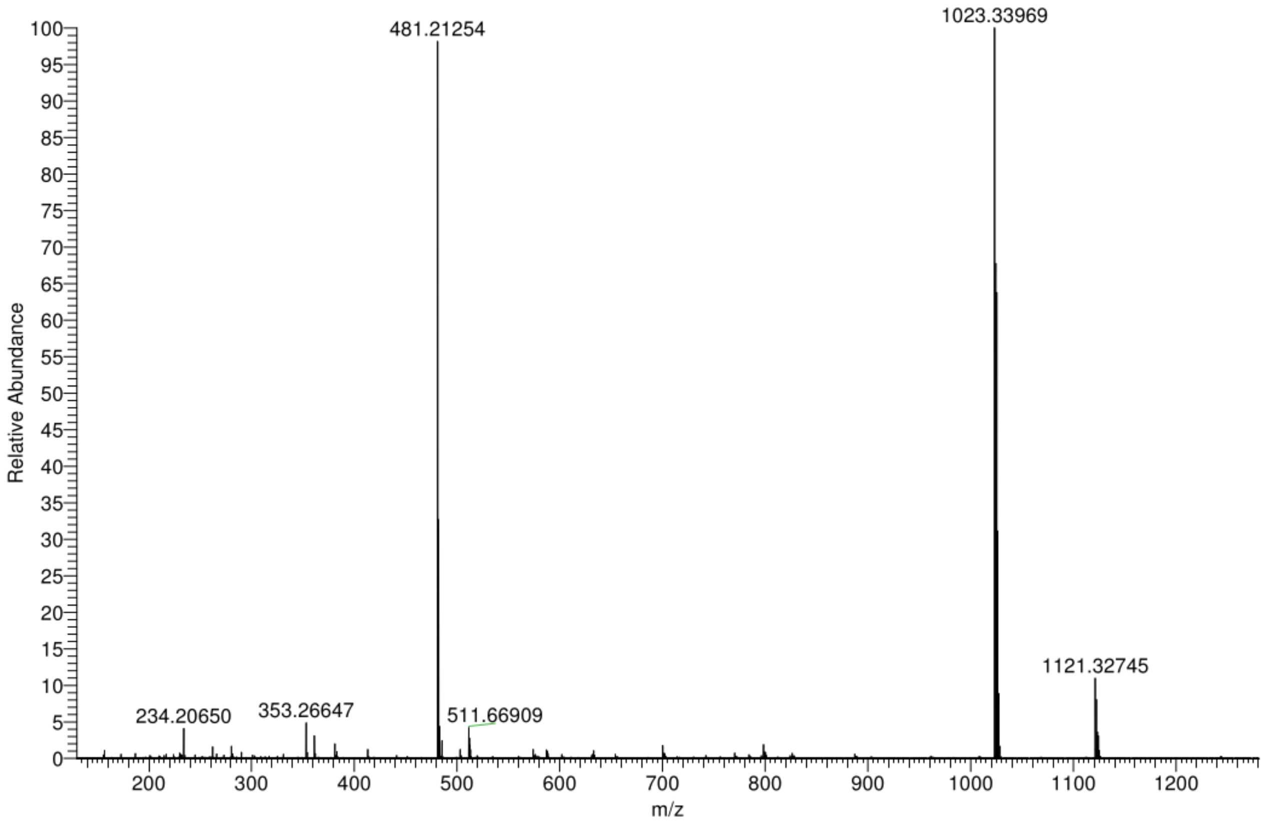

Figure S4.5. HRMS (ESI) of **C1'** in MeOH (fullscan).

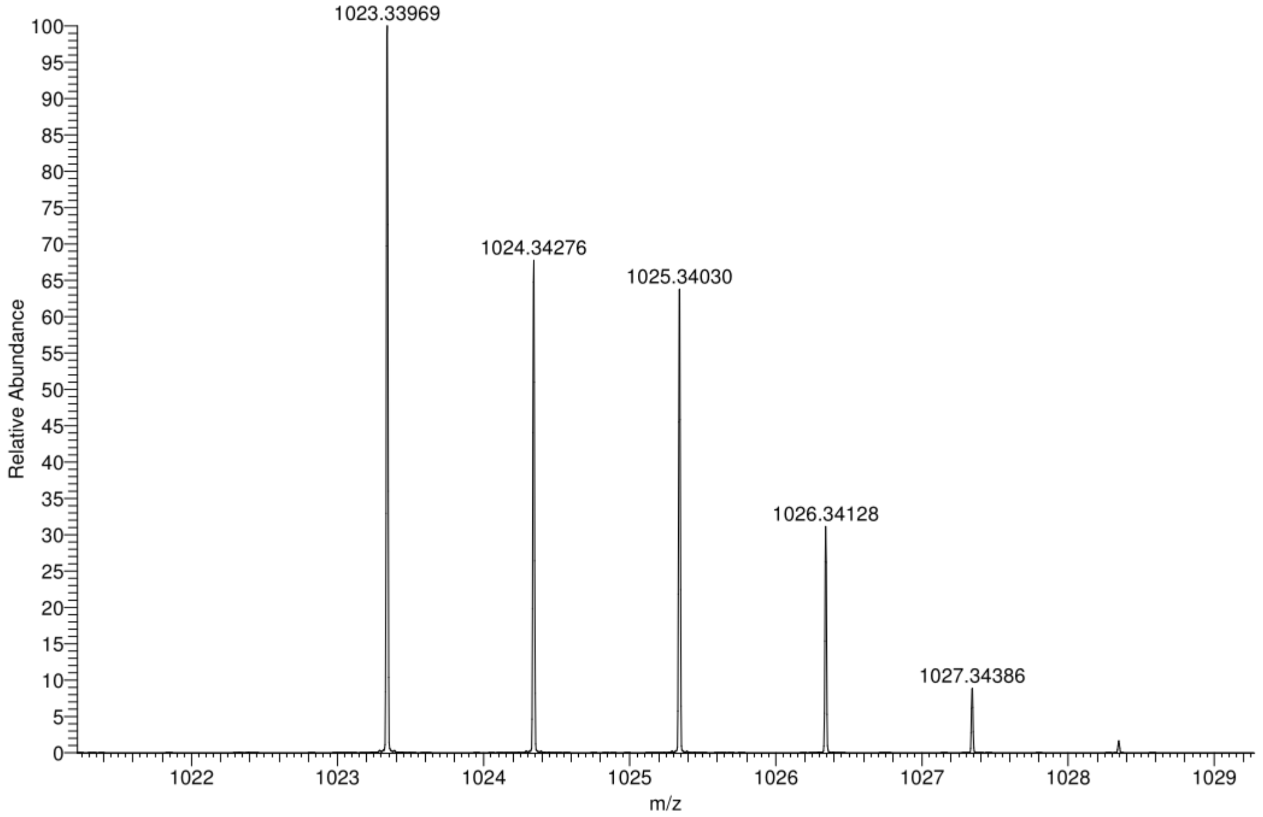

Figure S4.6. HRMS (ESI) of **C1'** in MeOH(zoom).

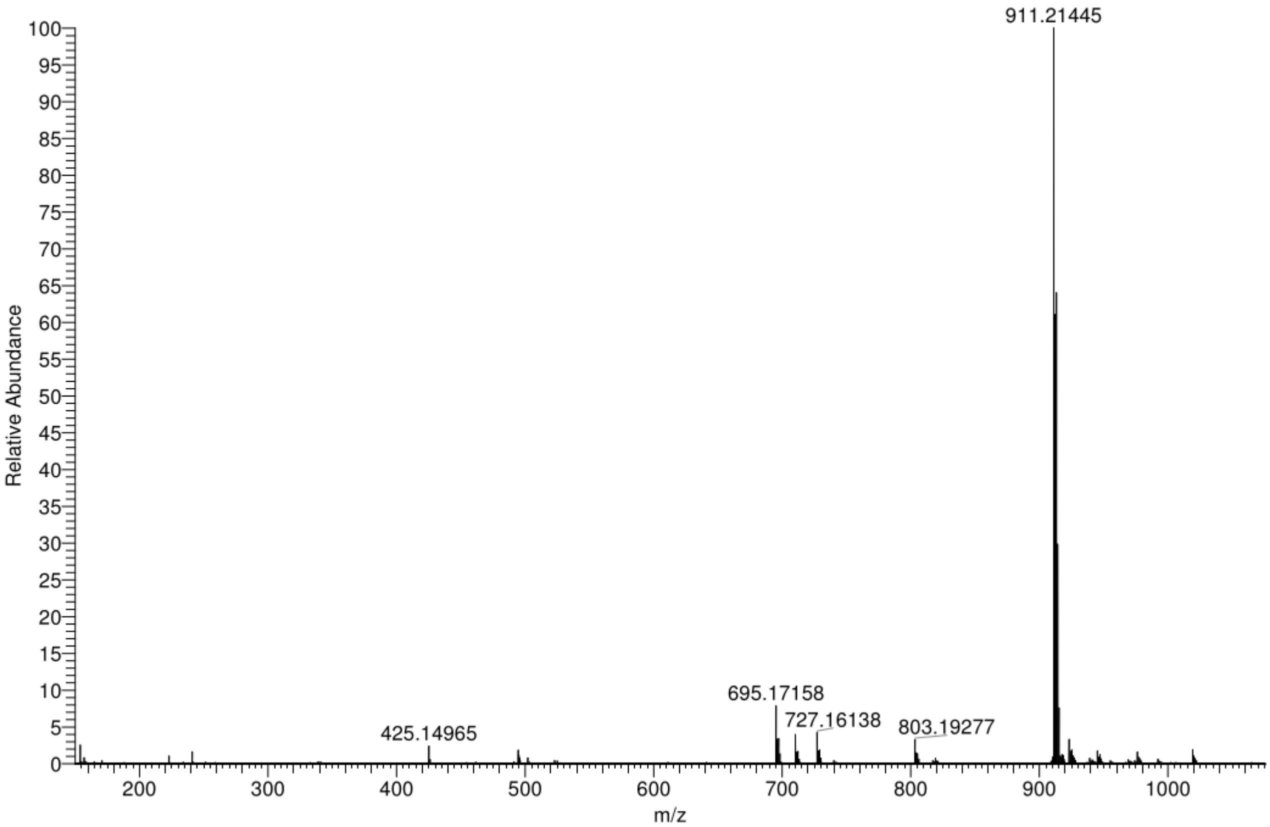

Figure S4.7. HRMS (ESI) of **C1** in MeOH (fullscan).

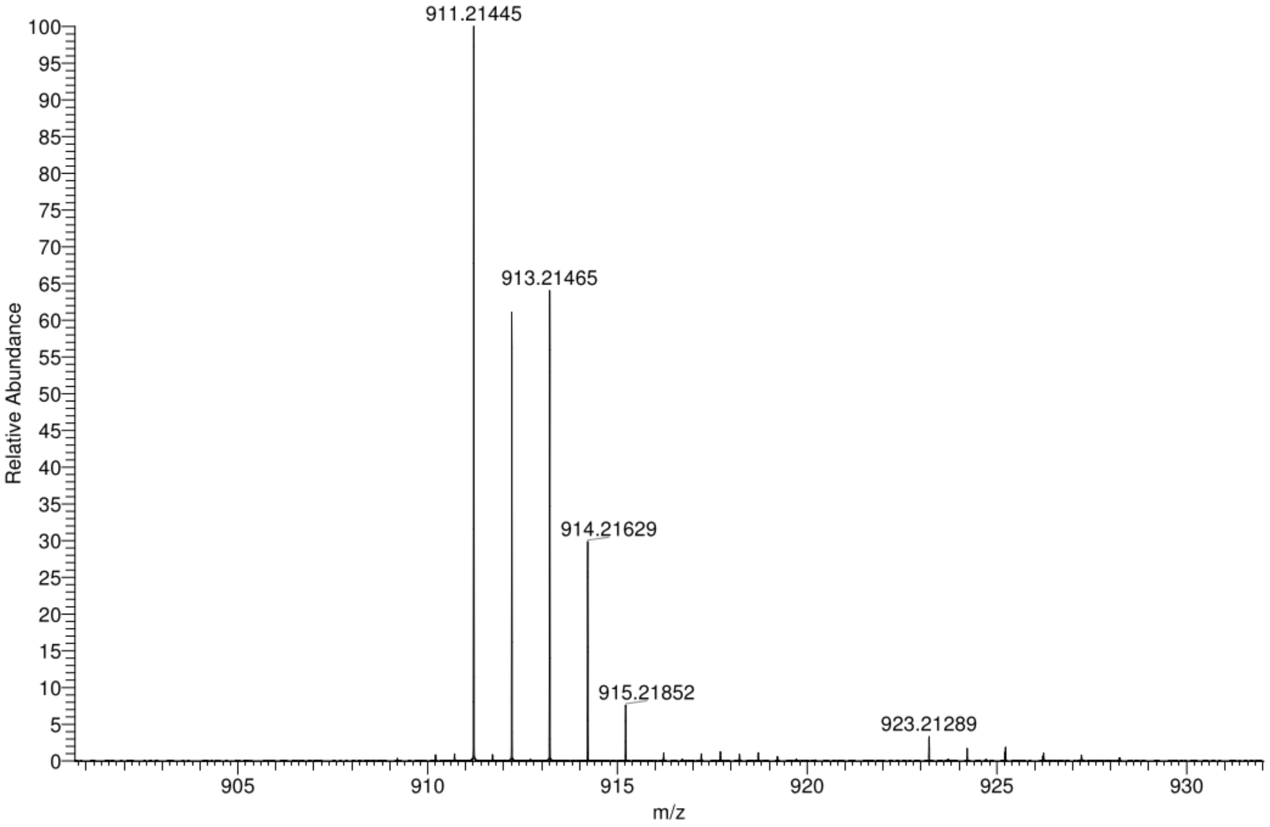

Figure S4.8. HRMS (ESI) of **C1** in MeOH(zoom).

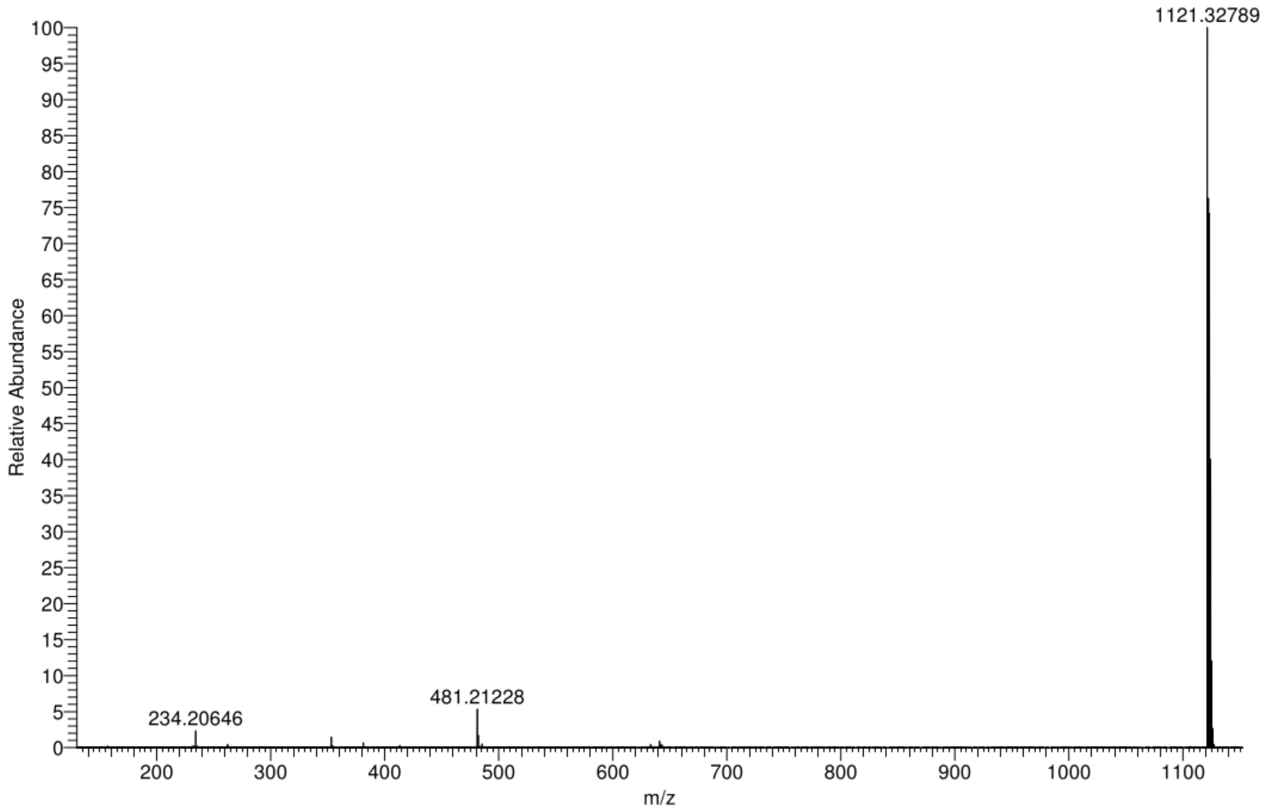

Figure S4.9. HRMS (ESI) of **C2'** in MeOH (fullscan).

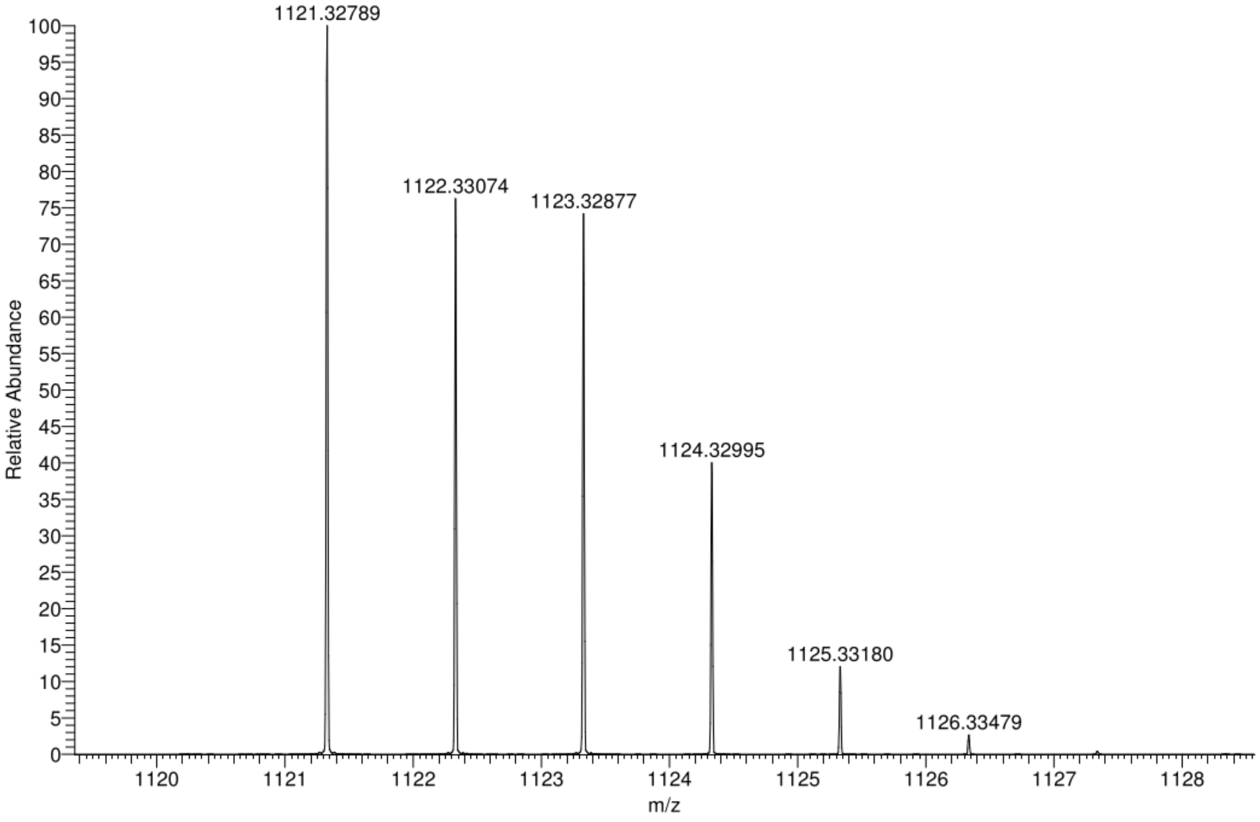

Figure S4.10. HRMS (ESI) of **C2'** in MeOH (zoom).

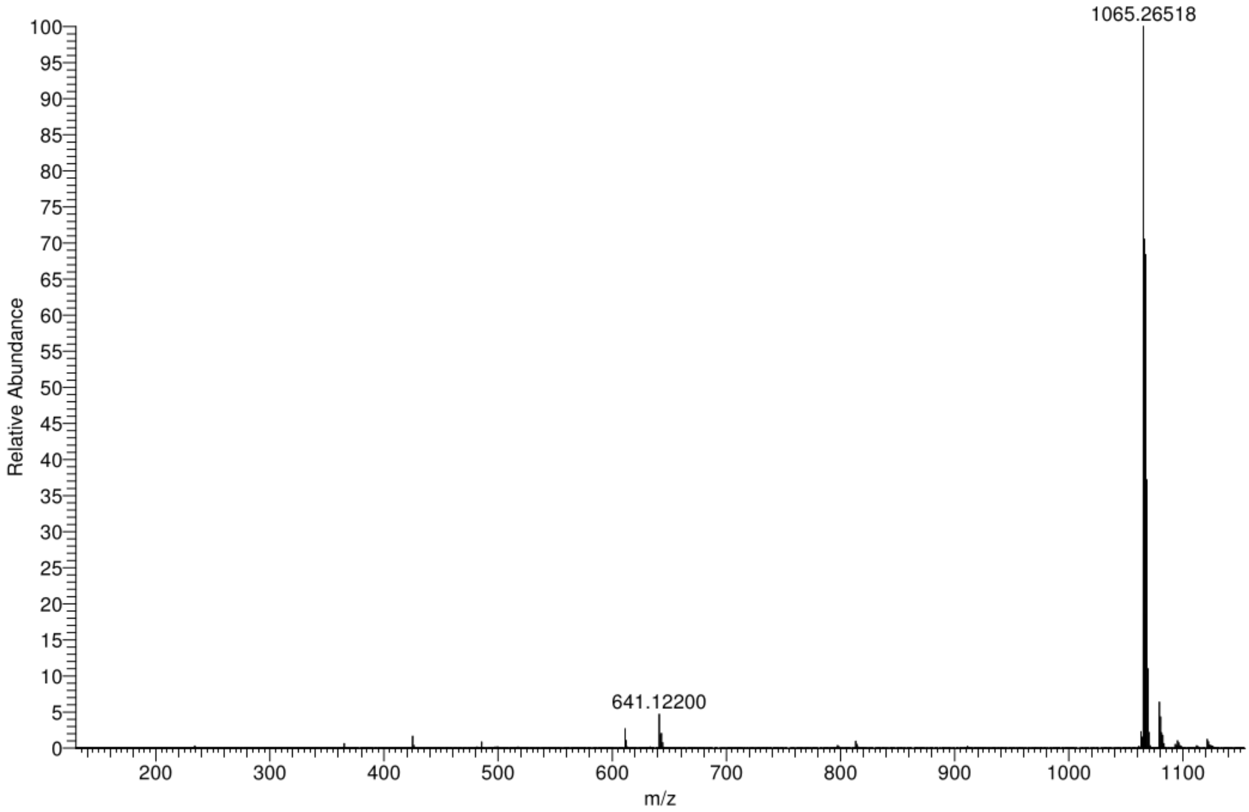

Figure S4.11. HRMS (ESI) of complex **C2** in MeOH (fullscan).

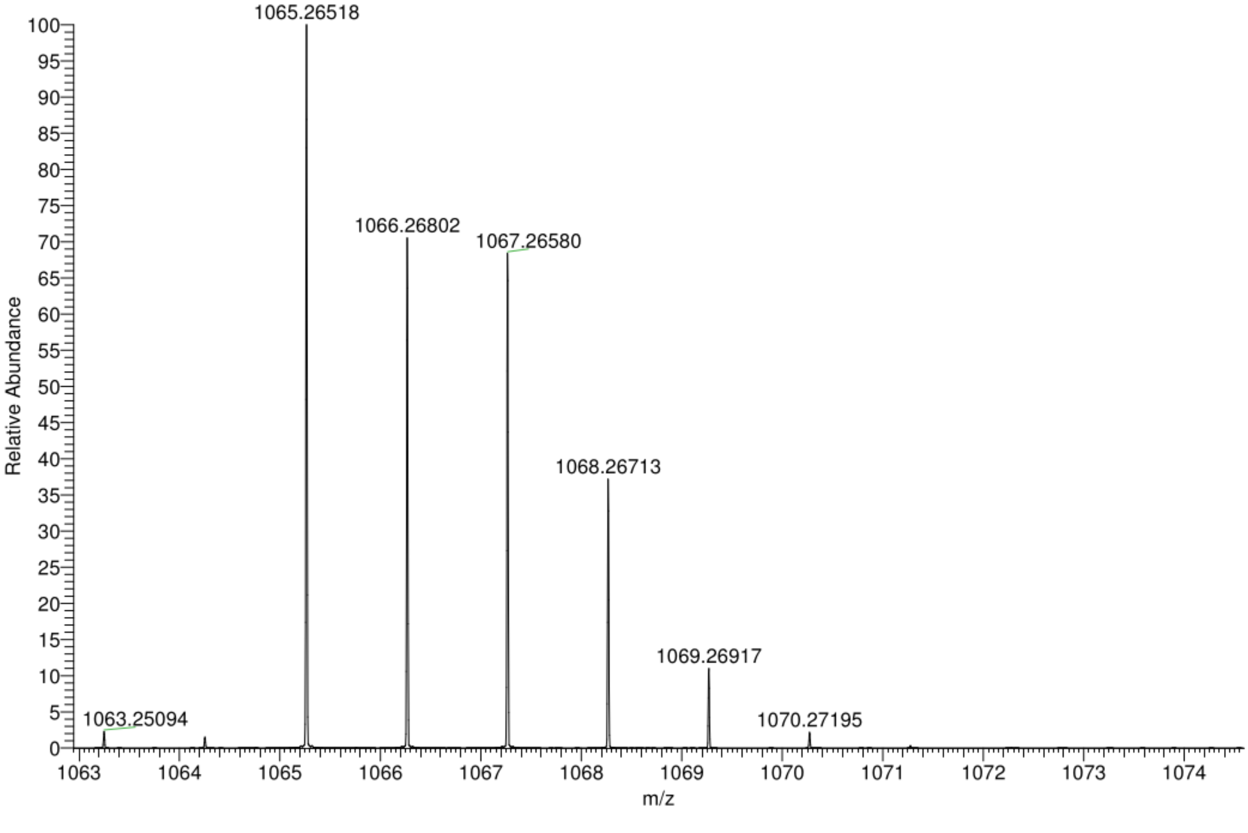

Figure S4.12. HRMS (ESI) of complex **C2** in MeOH (zoom).

5 Crystallographic data and solid-state molecular structure of C2'

Single crystals of **C2'** were obtained by crystallization from a concentrated dichloromethane/*n*-heptane solution at room temperature. Crystal growth was completed after four days at room temperature.

Table S5.1. Crystallographic data of the structural determinations of **C2'**.

	**C2'**$(CH_2Cl_2)_2$[a]
Chemical Formula	$C_{69}H_{60}CuN_2O_5P_2$, F_6P, 2 (CH_2Cl_2)
Formula mass	1267.71 g mol^{-1}
Crystal shape, color	fragment of rod, clear bright yellow
Crystal size	0.58 × 0.22 × 0.22 mm^3
Temperature, Radiation	100(2) K, 0.71073 Å
Abs. coefficient	0.631 mm^{-1}
Crystal system	Monoclinic
Space group type (no.)	$P2_{1/c}$ (14)
Z	4
a, b, c	12.6677(2) Å, 18.7246(3) Å, 28.5620(4) Å
α, β, γ	90°, 101.0530(10)°, 90°
Volume	6649.17(18) Å^3
Refl. collected	526981
indep.	30110
observed [$I>2\sigma(I)$]	24485
Data collection ranges	$-20 \le h \le 20$ / $-28 \le k \le 30$ / $-47 \le l \le 47$
Completeness to $\theta = 35.000°$	99.5%
Data / restr. / param.	30110 / 6 / 857
R_{int}	0.0398
R_1 [$I > 2\sigma(I)$]	0.0480
wR_2 (all data)	0.1297
GoF on F^2	1.037
Largest peak/hole	0.929/–1.168e Å^{-3}
CCDC Number	CCDC 2431283

[a] Two disordered CH_2Cl_2 molecules were refined using split atom models (0.9412(9) and 0.960(1) SOF of main component), applying geometric similarity restraints (SAME). A common ADP was refined for each disordered atom pair (EADP).

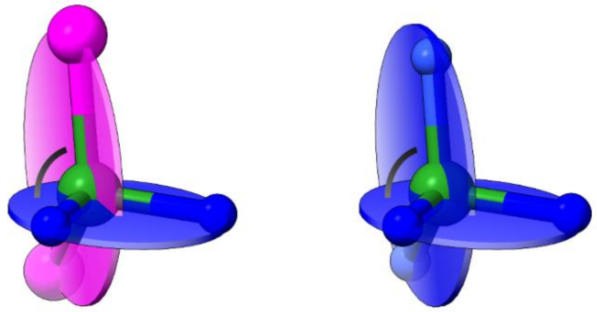

Figure S5.1. Definition of the interplane angle for the heteroleptic complexes (PP-Cu-NN, left) and the homoleptic complexes (NN-Cu-NN, right).

6 Density functional theory (DFT)

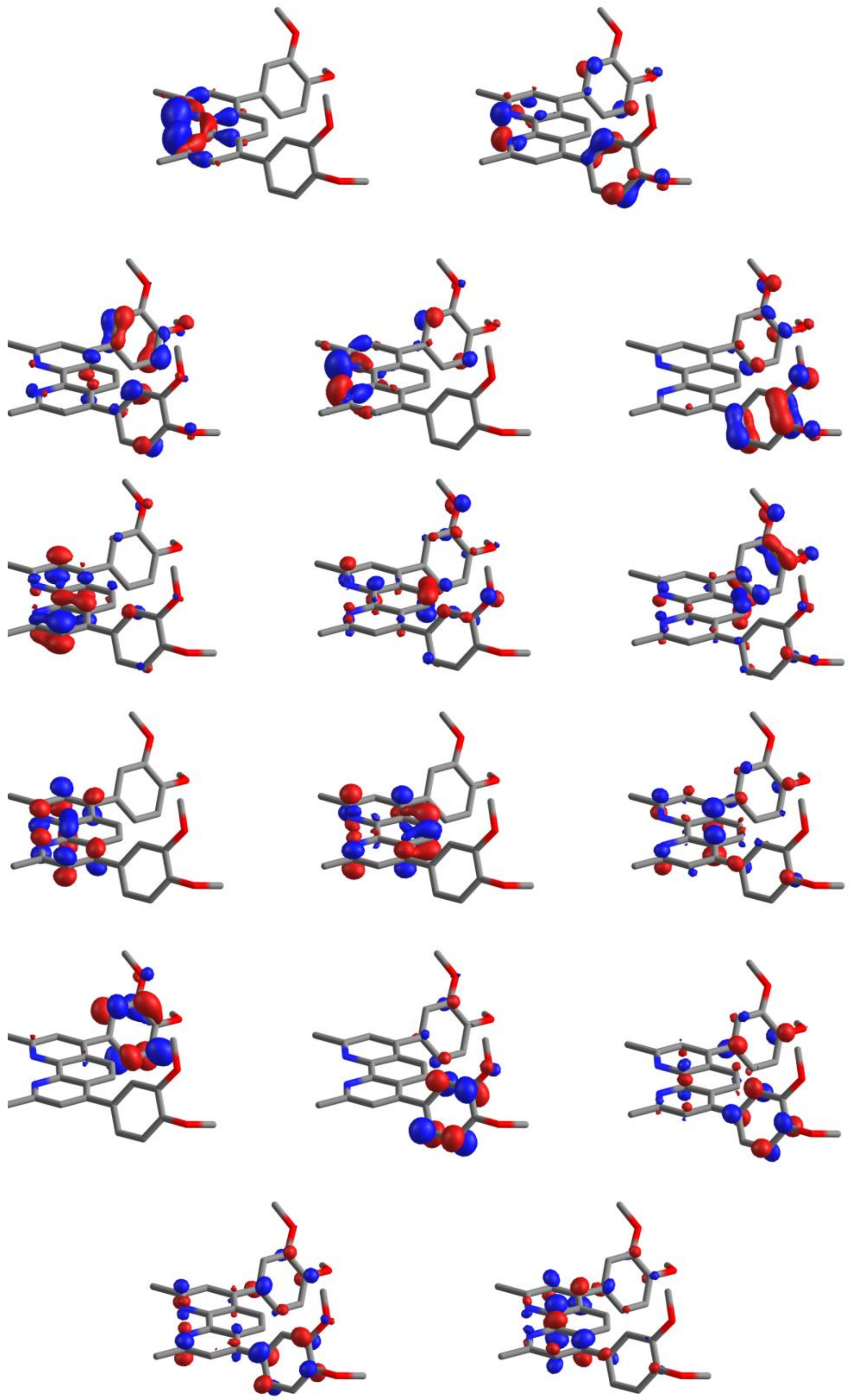

Figure S6.1. Orbital representation of **L1'** showing from top left to bottom right the HOMO-7, HOMO-6, HOMO-5, HOMO-4, HOMO-3, HOMO-2, HOMO-1, HOMO, LUMO, LUMO+1, LUMO+2, LUMO+3, LUMO+4, LUMO+5, LUMO+6 and LUMO+7.

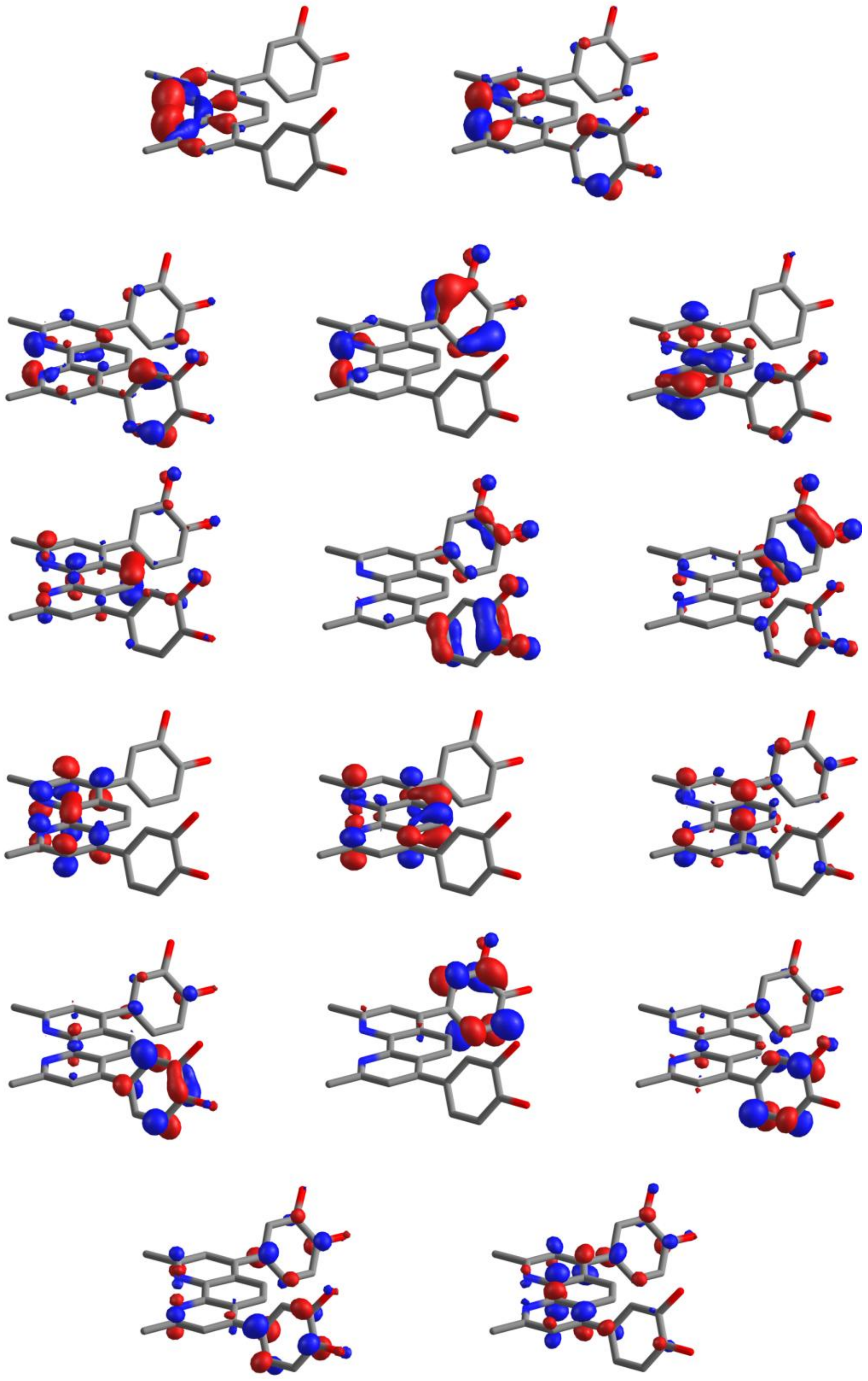

Figure S6.2. Orbital representation of **L1** showing from top left to bottom right the HOMO-7, HOMO-6, HOMO-5, HOMO-4, HOMO-3, HOMO-2, HOMO-1, HOMO, LUMO, LUMO+1, LUMO+2, LUMO+3, LUMO+4, LUMO+5, LUMO+6 and LUMO+7.

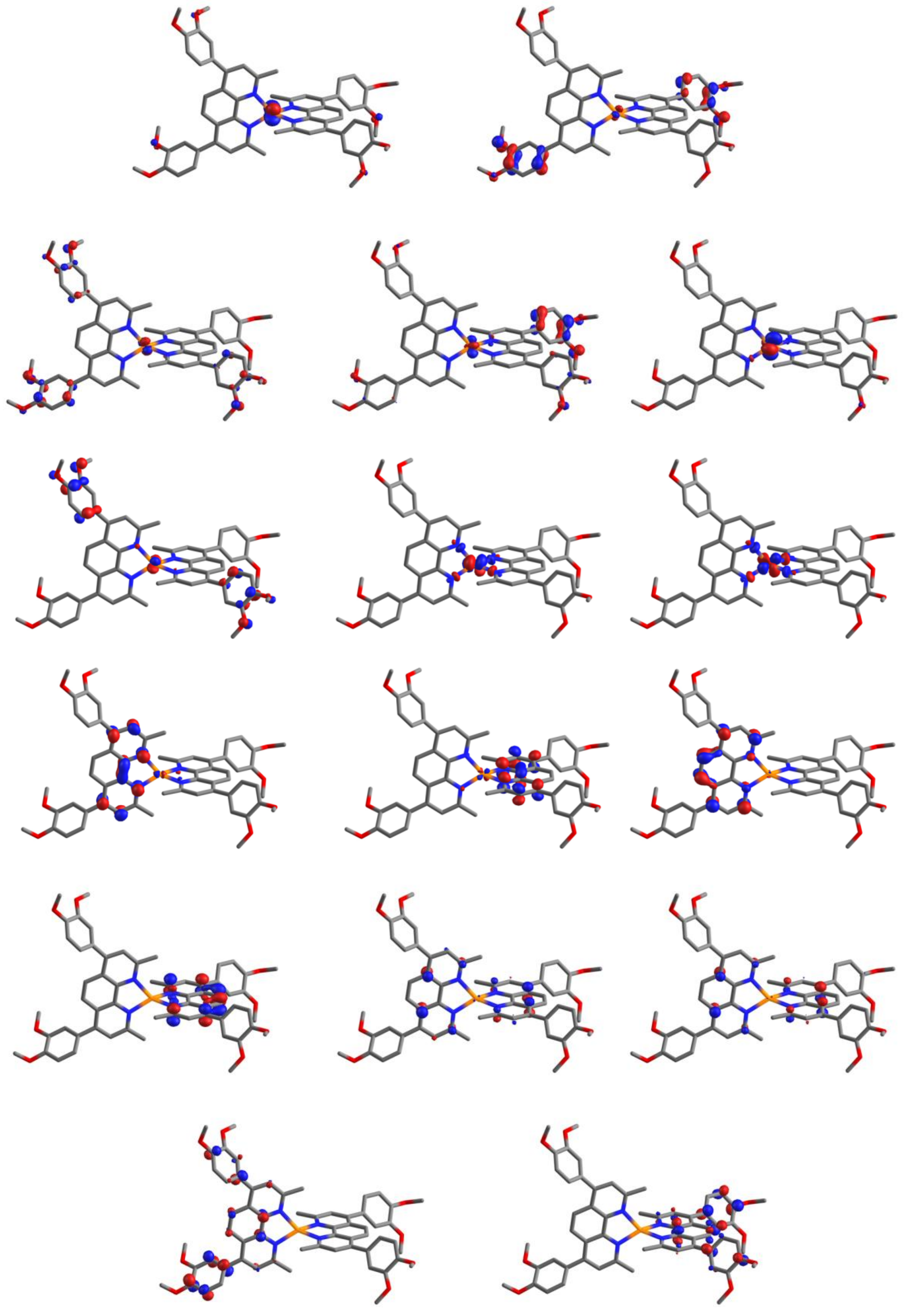

Figure S6.3. Orbital representation of **C1'** showing from top left to bottom right the HOMO-7, HOMO-6, HOMO-5, HOMO-4, HOMO-3, HOMO-2, HOMO-1, HOMO, LUMO, LUMO+1, LUMO+2, LUMO+3, LUMO+4, LUMO+5, LUMO+6 and LUMO+7.

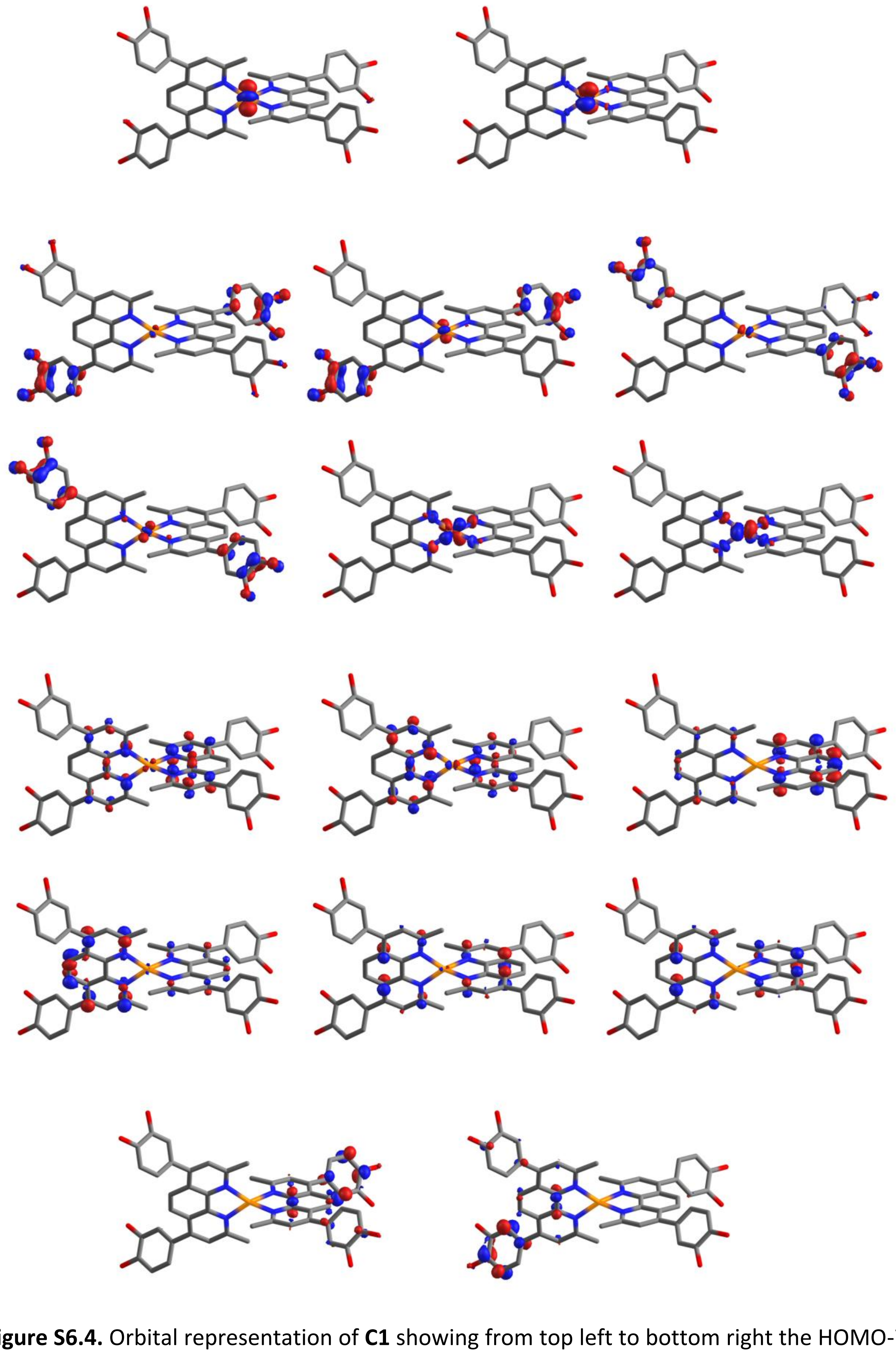

Figure S6.4. Orbital representation of **C1** showing from top left to bottom right the HOMO-7, HOMO-6, HOMO-5, HOMO-4, HOMO-3, HOMO-2, HOMO-1, HOMO, LUMO, LUMO+1, LUMO+2, LUMO+3, LUMO+4, LUMO+5, LUMO+6 and LUMO+7.

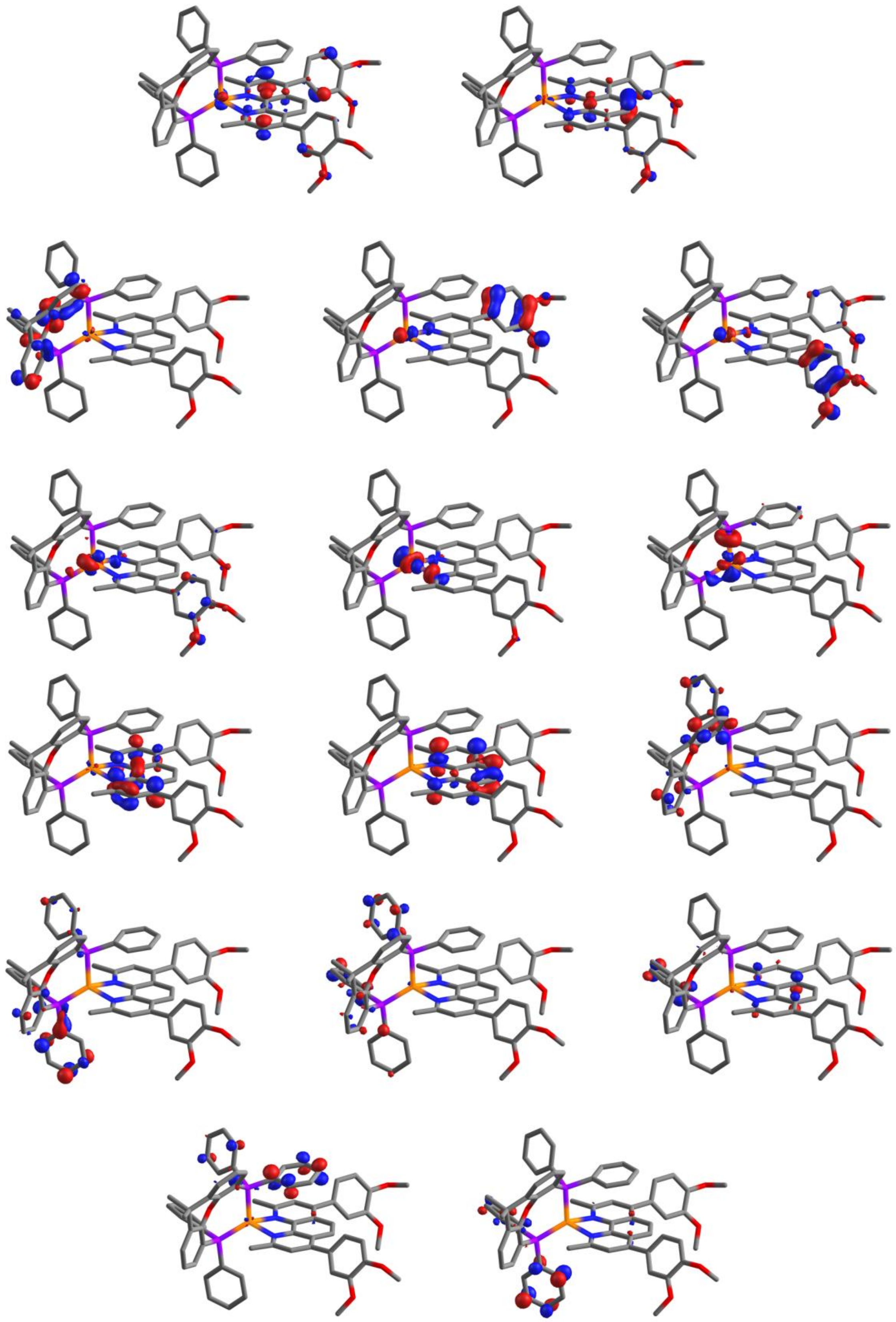

Figure S6.5. Orbital representation of **C2'** showing from top left to bottom right the HOMO-7, HOMO-6, HOMO-5, HOMO-4, HOMO-3, HOMO-2, HOMO-1, HOMO, LUMO, LUMO+1, LUMO+2, LUMO+3, LUMO+4, LUMO+5, LUMO+6 and LUMO+7.

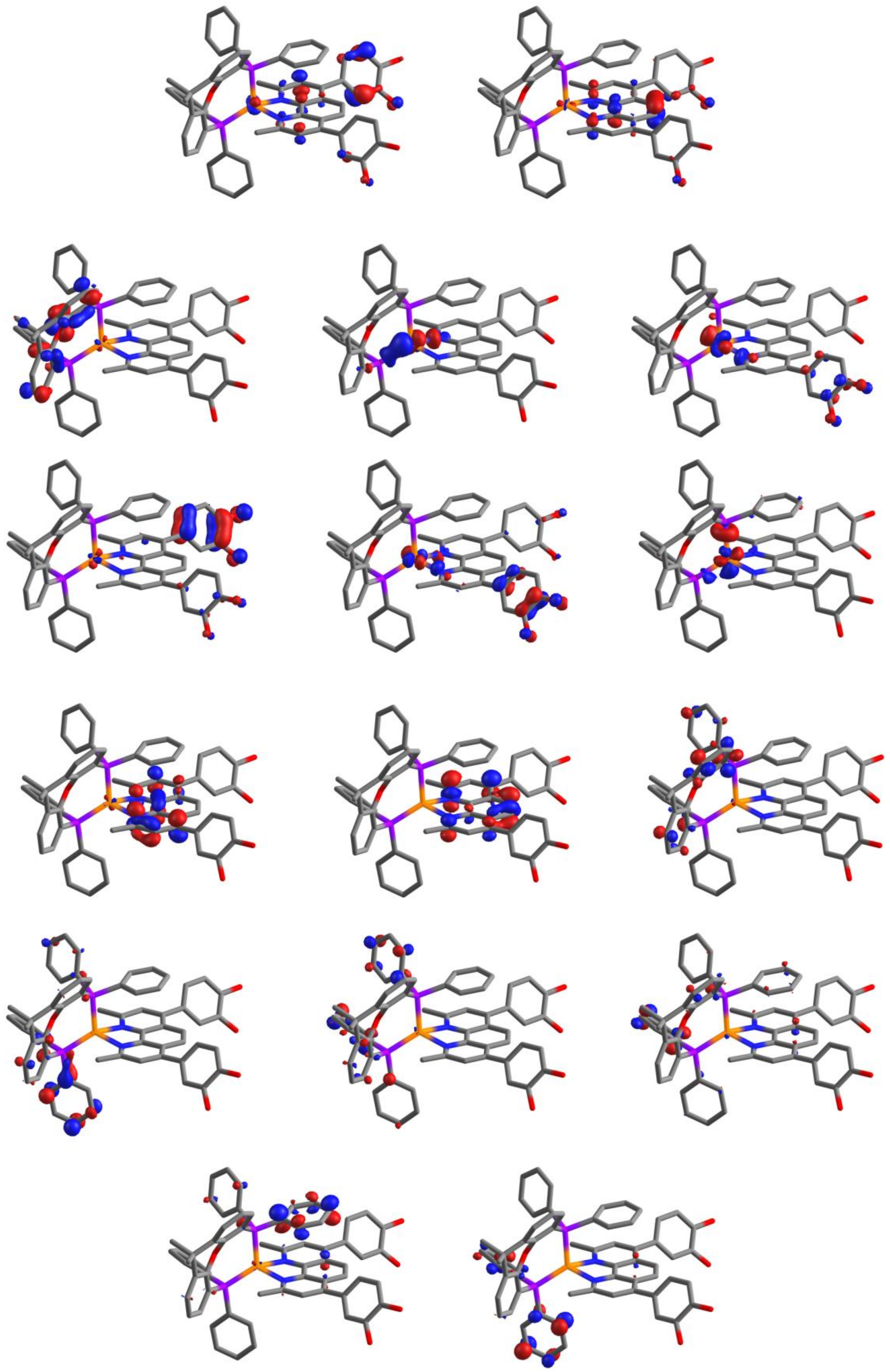

Figure S6.6. Orbital representation of **C2** showing from top left to bottom right the HOMO-7, HOMO-6, HOMO-5, HOMO-4, HOMO-3, HOMO-2, HOMO-1, HOMO, LUMO, LUMO+1, LUMO+2, LUMO+3, LUMO+4, LUMO+5, LUMO+6 and LUMO+7.

7 Time-dependent density functional theory (TDDFT)

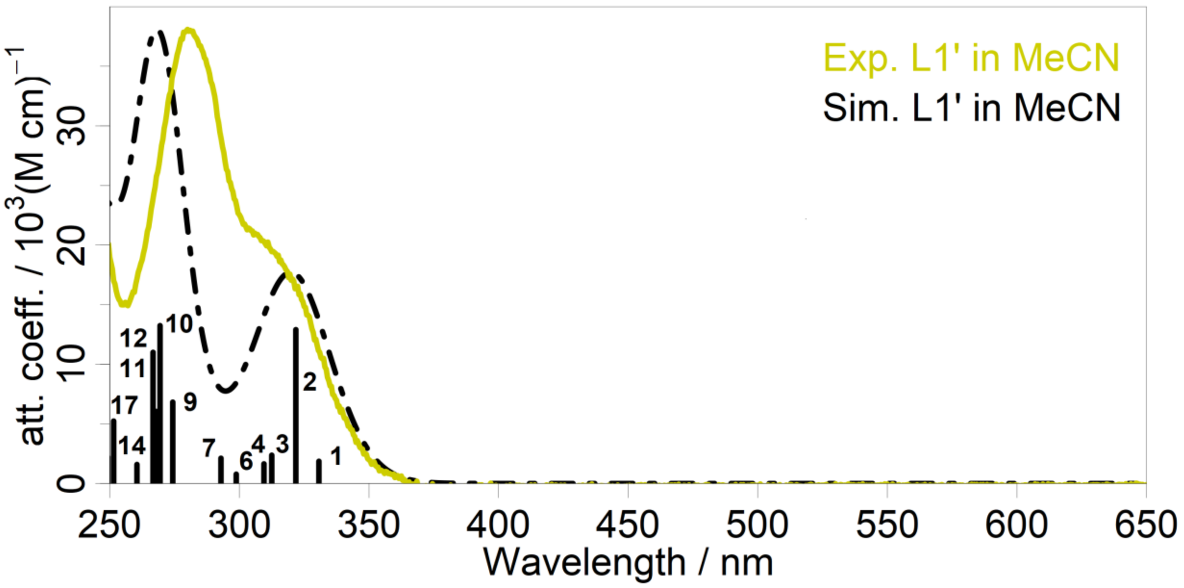

Figure S7.1. Calculated absorption spectrum (dotted dashed, black) and experimental UV/vis spectrum (solid, yellow) of **L1'** in acetonitrile solution. In the calculated spectrum only excitations with oscillator strength above 0.01 are shown. Selected excitations are annotated according to their transition number.

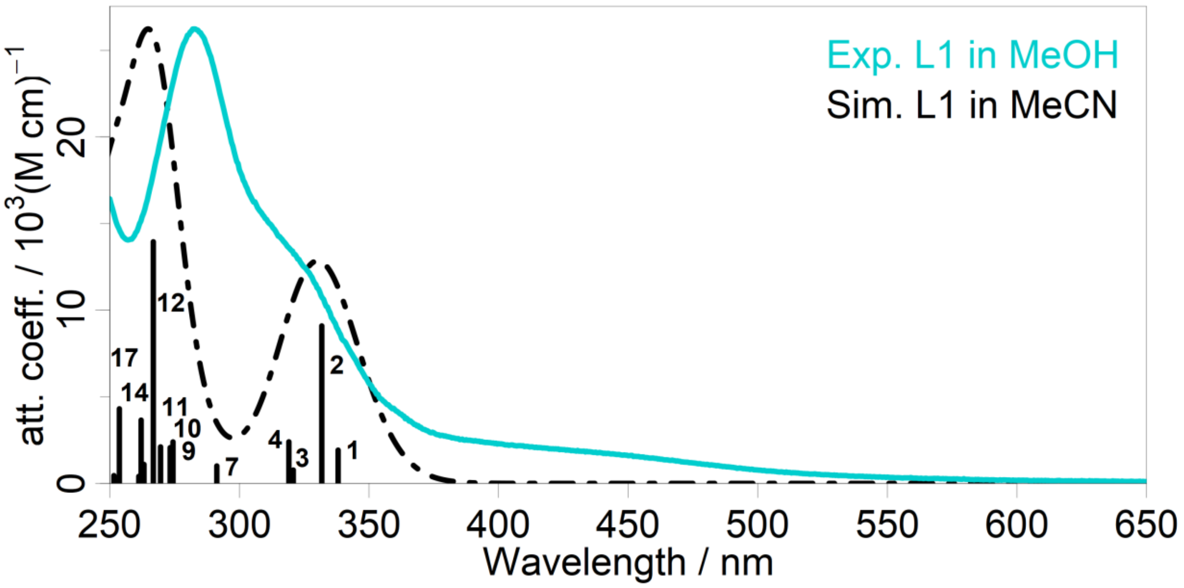

Figure S7.2. Calculated absorption spectrum (dotted dashed, black) simulated in acetonitrile solution and experimental UV/vis spectrum (solid, cyan) in methanol solution of **L1**. In the calculated spectrum only excitations with oscillator strength above 0.01 are shown. Selected excitations are annotated according to their transition number.

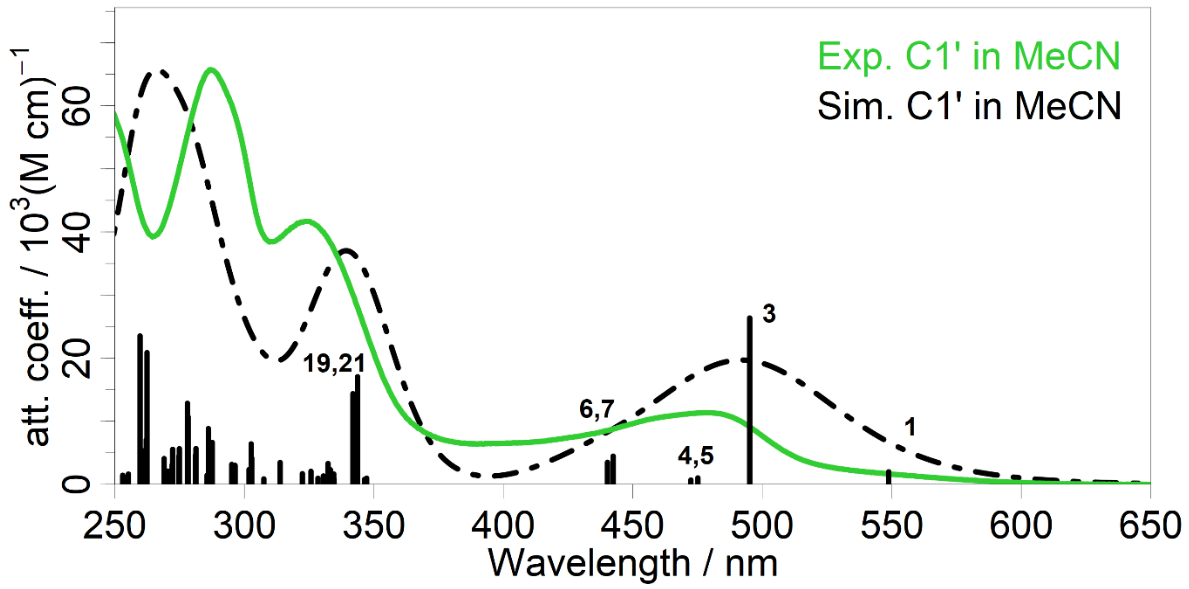

Figure S7.3. Calculated absorption spectrum (dotted dashed, black) and experimental UV/vis spectrum (solid, green) of **C1'** in acetonitrile solution. In the calculated spectrum only excitations with oscillator strength above 0.01 are shown. Selected excitations are annotated according to their transition number.

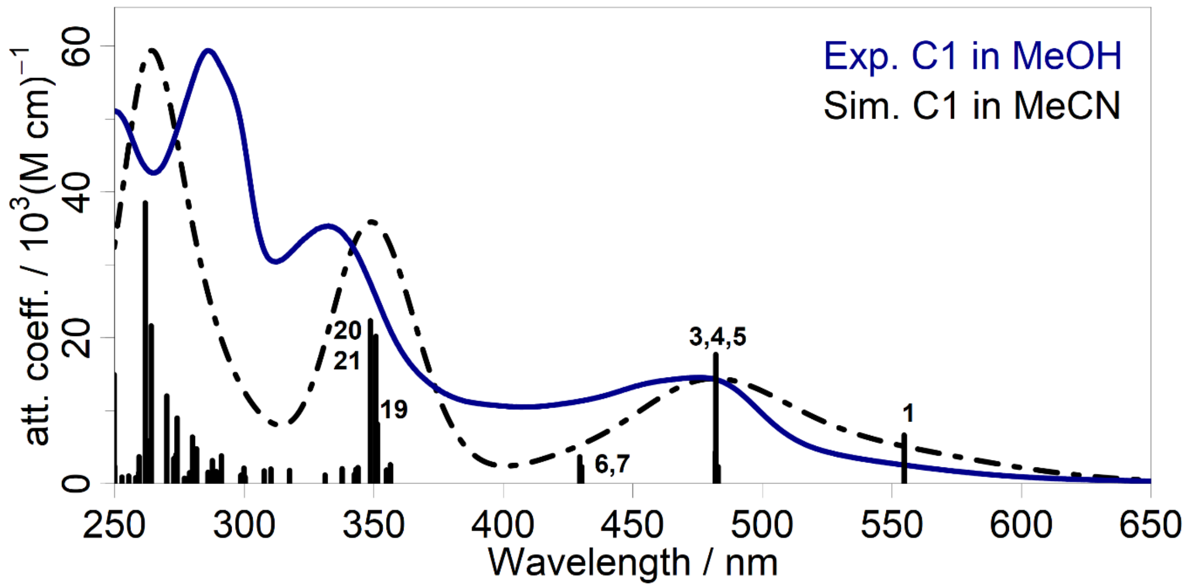

Figure S7.4. Calculated absorption spectrum (dotted dashed, black) simulated in acetonitrile solution and experimental UV/vis spectrum (solid, blue) in methanol solution of **C1**. In the calculated spectrum only excitations with oscillator strength above 0.01 are shown. Selected excitations are annotated according to their transition number.

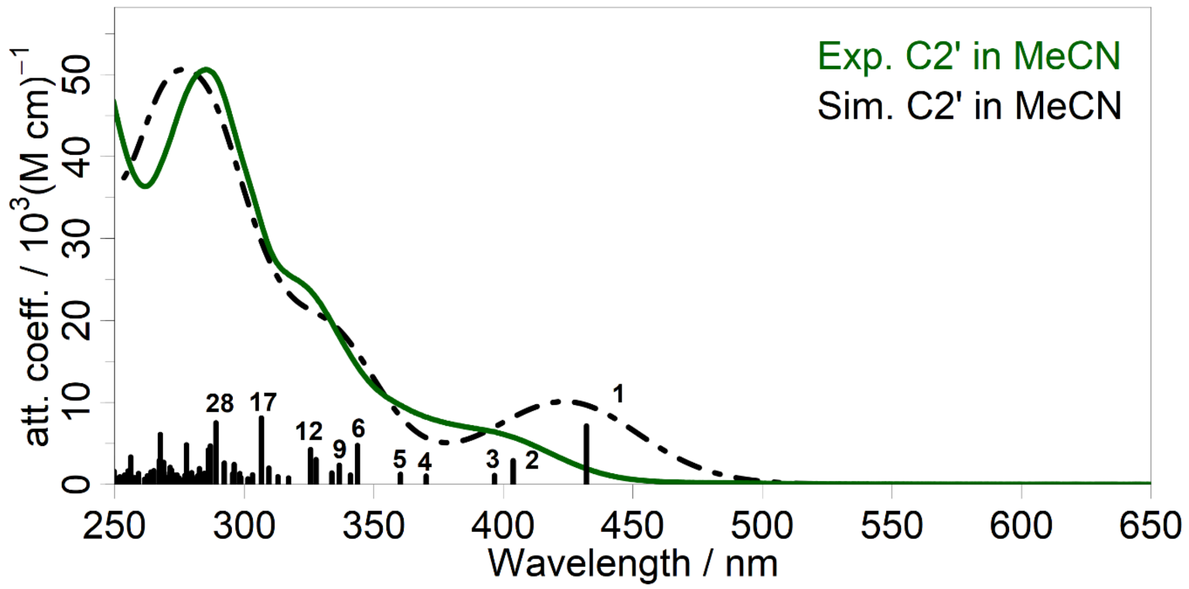

Figure S7.5. Calculated absorption spectrum (dotted dashed, black) and experimental UV/vis spectrum (solid, darkgreen) of **C2'** in acetonitrile solution. In the calculated spectrum only excitations with oscillator strength above 0.01 are shown. Selected excitations are annotated according to their transition number.

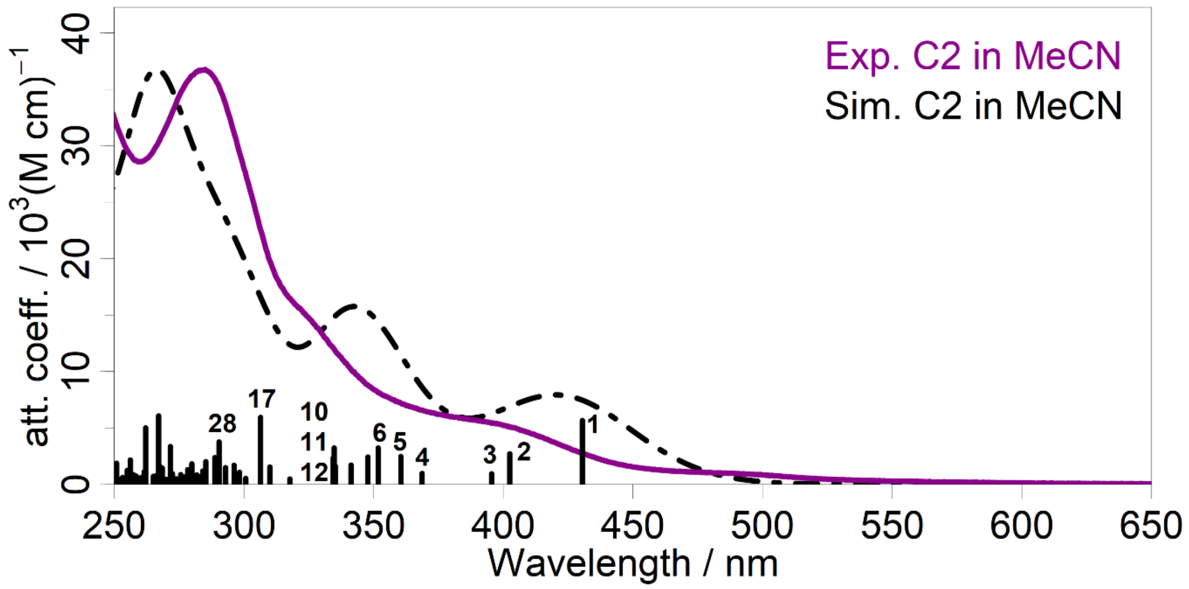

Figure S7.6. Calculated absorption spectrum (dotted dashed, black) and experimental UV/vis spectrum (solid, magenta) of **C2** in acetonitrile solution. In the calculated spectrum only excitations with oscillator strength above 0.01 are shown. Selected excitations are annotated according to their transition number.

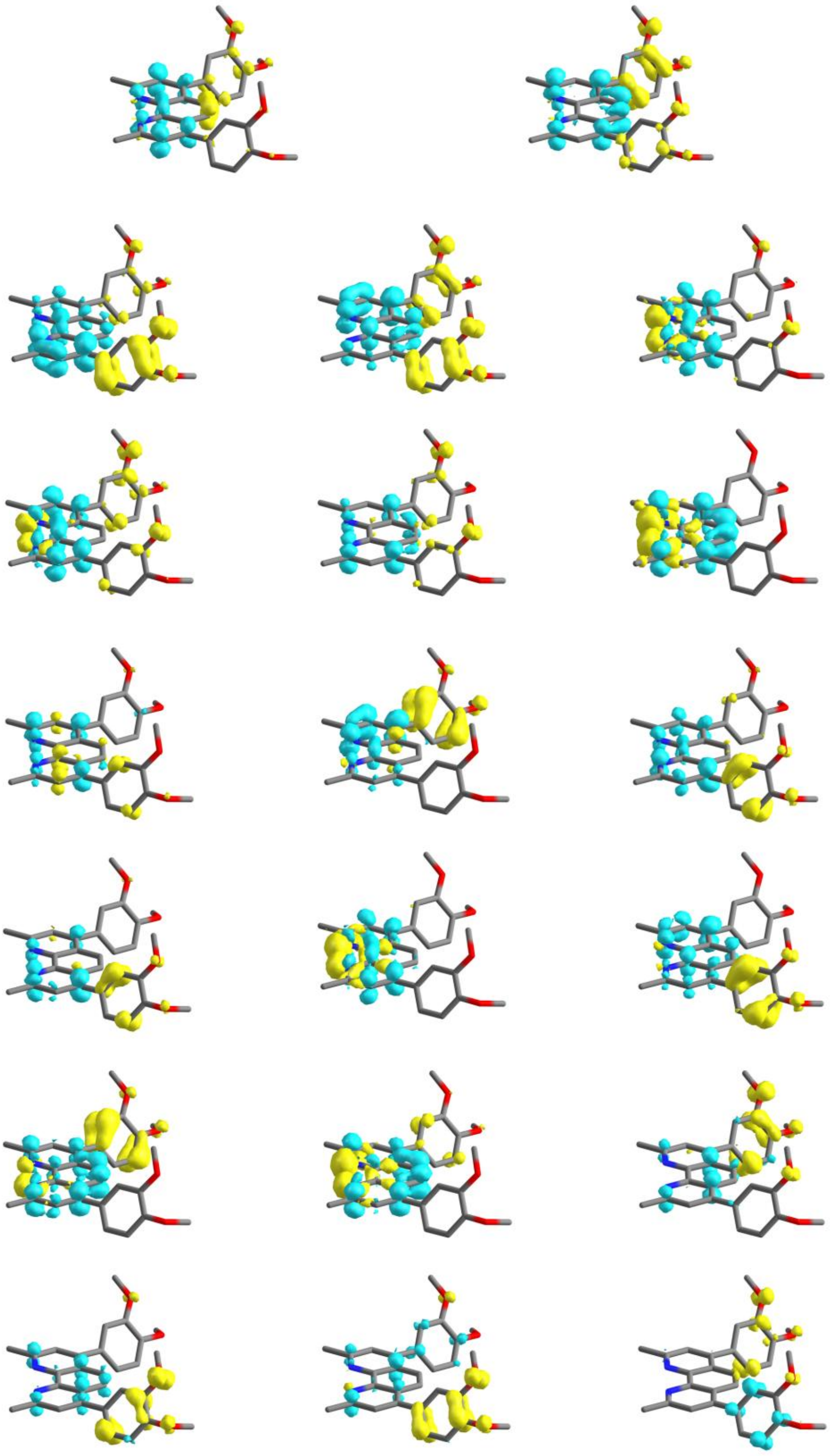

Figure S7.7. Electron difference density plots of **L1'** corresponding to the first 20 transitions (1-20 from top left to bottom right). Yellow depicts loss of electron density, while teal depicts gain of electron density during the transition.

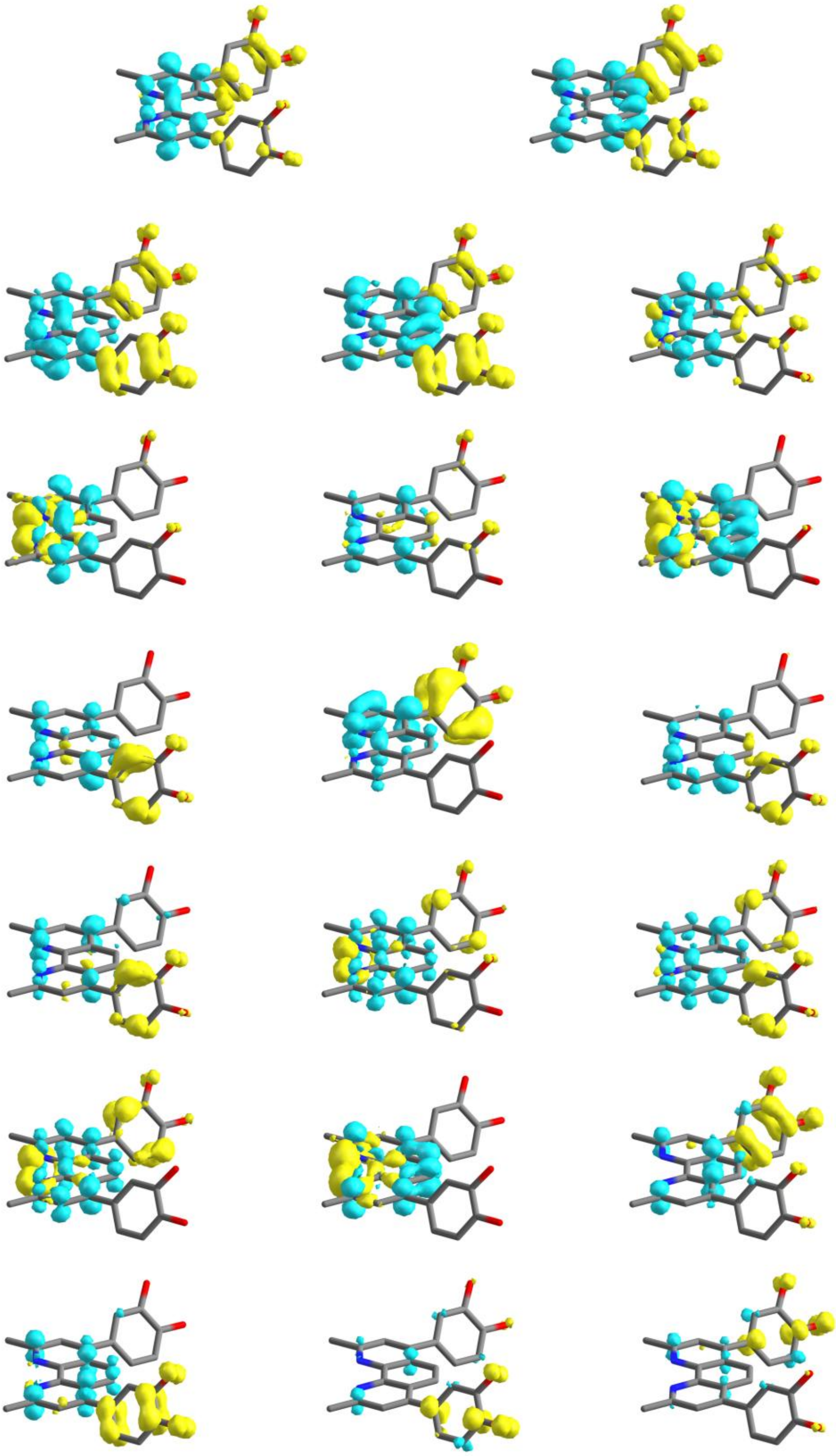

Figure S7.8. Electron difference density plots of **L1** corresponding to the first 20 transitions (1-20 from top left to bottom right). Yellow depicts loss of electron density, while teal depicts gain of electron density during the transition.

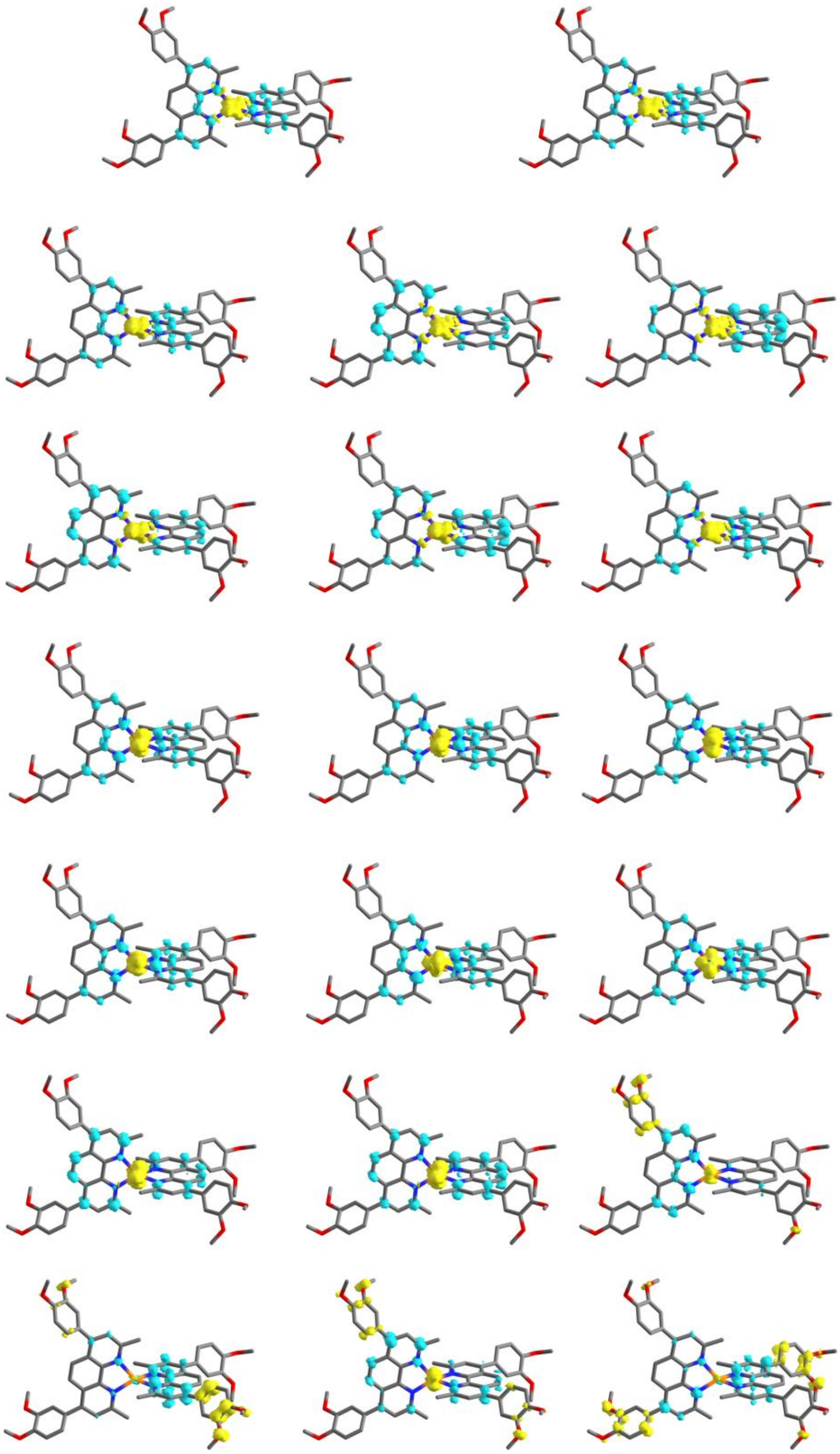

Figure S7.9. Electron difference density plots of **C1'** corresponding to the first 20 transitions (1-20 from top left to bottom right). Yellow depicts loss of electron density, while teal depicts gain of electron density during the transition.

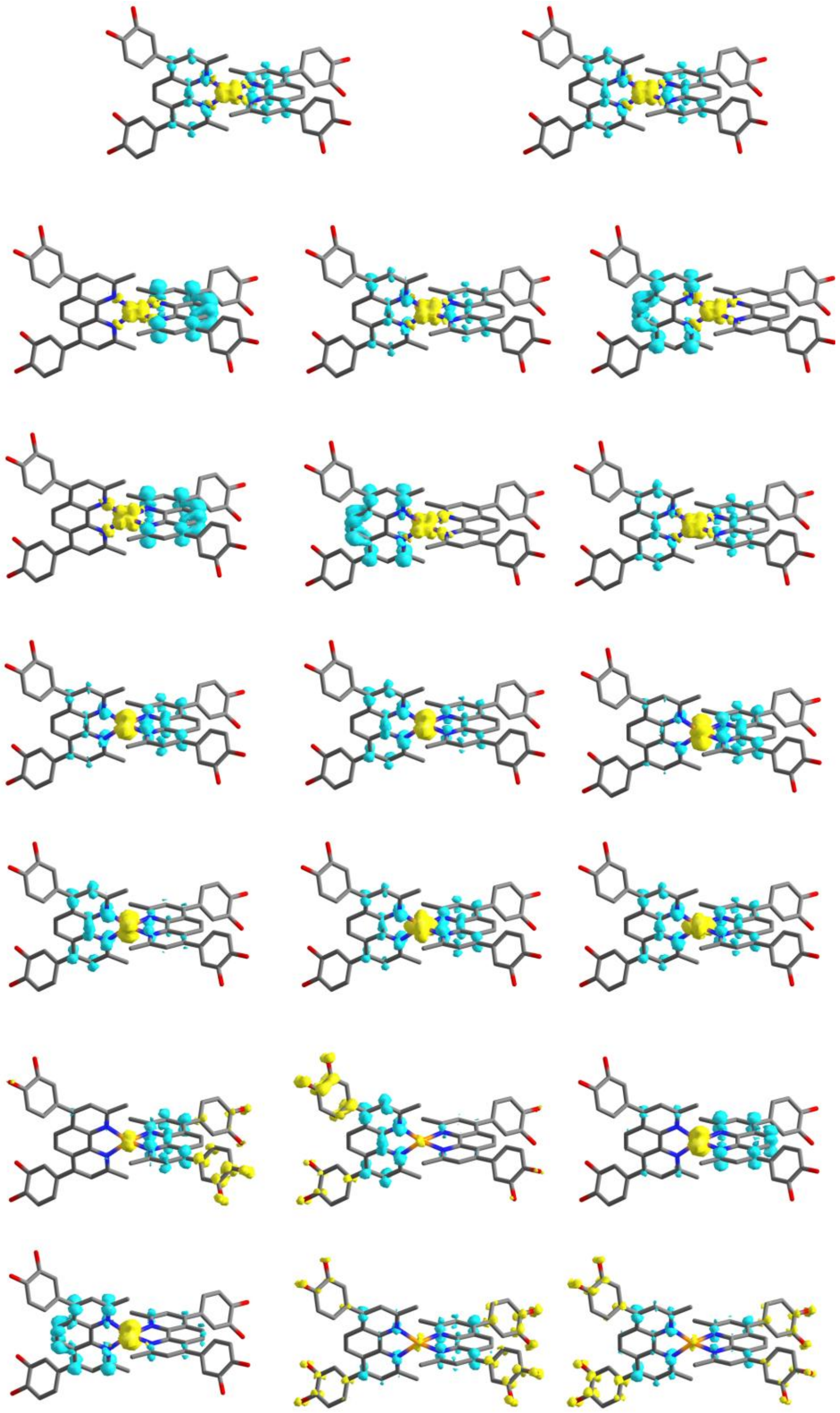

Figure S7.10. Electron difference density plots of **C1** corresponding to the first 20 transitions (1-20 from top left to bottom right). Yellow depicts loss of electron density, while teal depicts gain of electron density during the transition.

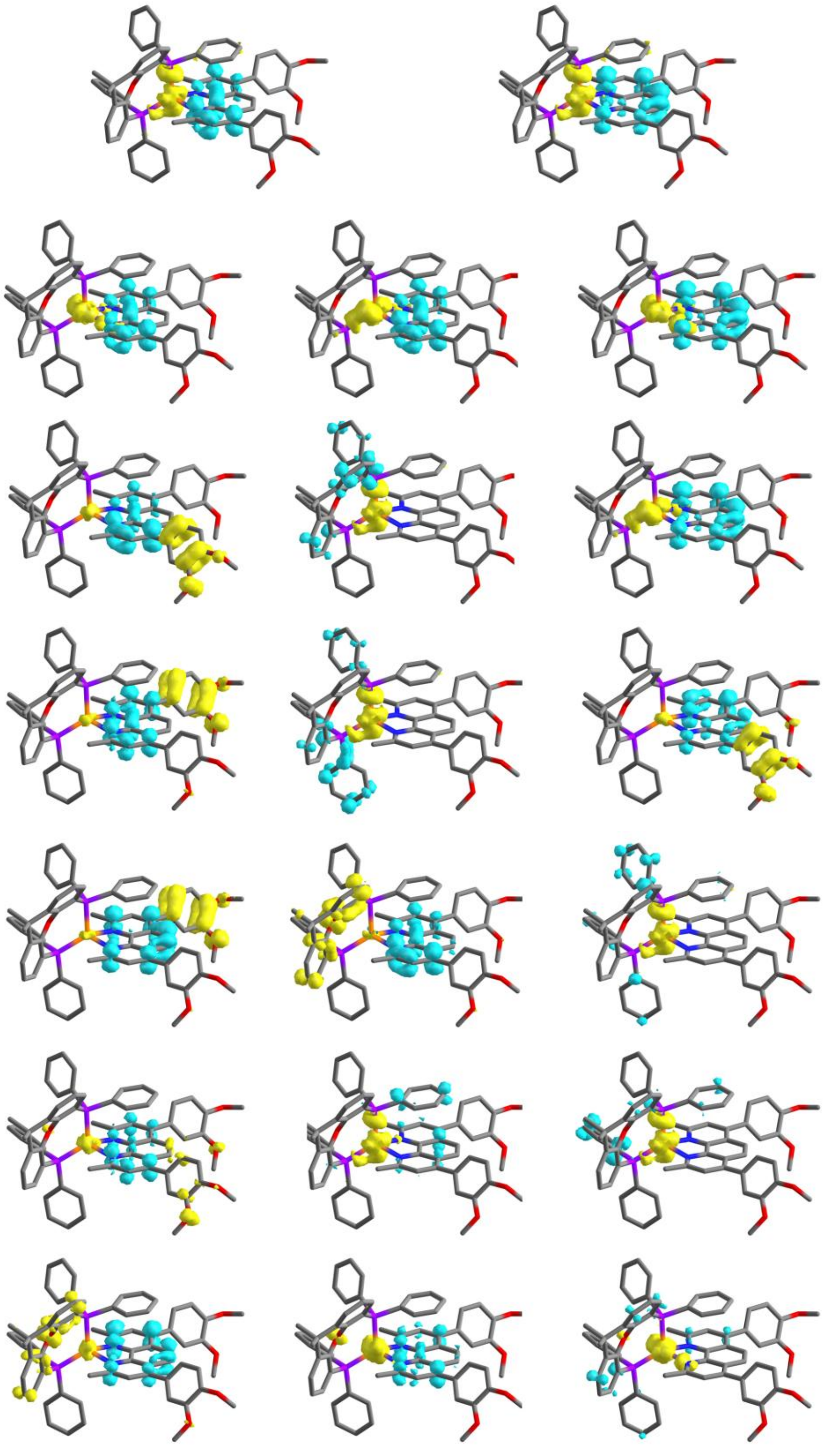

Figure 7.11. Electron difference density plots of **C2'** corresponding to the first 20 transitions (1-20 from top left to bottom right). Yellow depicts loss of electron density, while teal depicts gain of electron density during the transition.

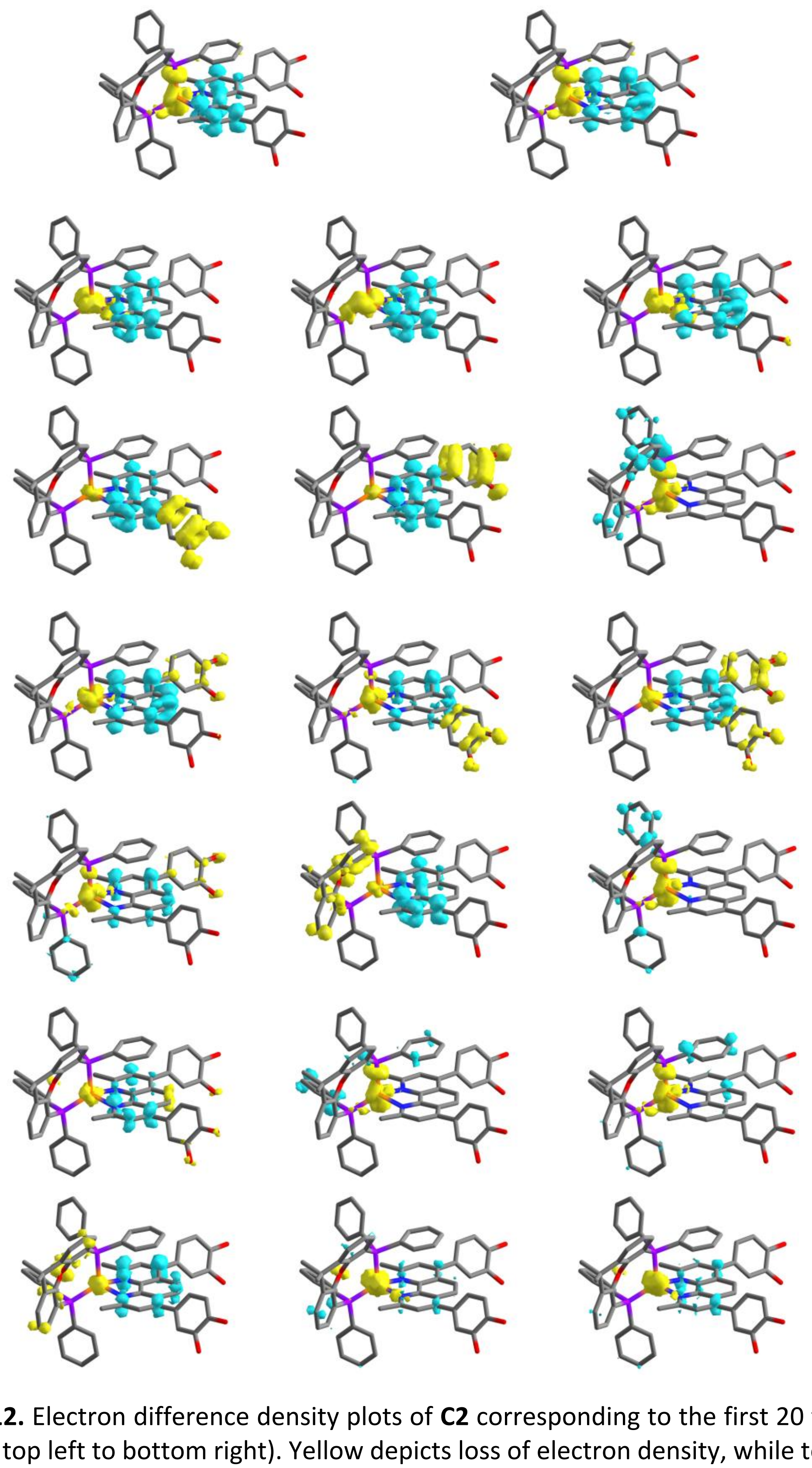

Figure S7.12. Electron difference density plots of **C2** corresponding to the first 20 transitions (1-20 from top left to bottom right). Yellow depicts loss of electron density, while teal depicts gain of electron density during the transition.

Table S7.1. Excitation energies, transition moments and corresponding transitions of **L1'** obtained from TDDFT simulated in acetonitrile. Note that only excitations with an oscillator strength > 0.01 and corresponding orbital contributions with an OC ≥ 0.1 ($|coeff.|^2 100 \geq 0.1$) are shown. Molecular fragments that show only minor contribution to the mixing of orbitals are given in parenthesis (*e.g.*(π_{phen})).

State #	Exc. energy cm^{-1}	nm	Oscillator strength	Dominant contribution OC	occ. orb.	virt. orb.	Transition
1	30238.2	330.7	0.049	0.831	HOMO	LUMO	$\pi_{sub},(\pi_{phen})\rightarrow\pi^*_{phen}$
2	31078.4	321.8	0.339	0.161	HOMO-1	LUMO	$\pi_{phen},\pi_{sub}\rightarrow\pi^*_{phen}$
				0.780	HOMO	LUMO+1	$\pi_{sub},(\pi_{phen})\rightarrow\pi^*_{phen}$
3	31998.1	312.5	0.062	0.165	HOMO-2	LUMO	$\pi_{phen},(\pi_{sub})\rightarrow\pi^*_{phen}$
				0.415	HOMO-1	LUMO	$\pi_{phen},\pi_{sub}\rightarrow\pi^*_{phen}$
				0.296	HOMO-1	LUMO+1	$\pi_{phen},\pi_{sub}\rightarrow\pi^*_{phen}$
4	32309.7	309.5	0.043	0.372	HOMO-1	LUMO	$\pi_{phen},\pi_{sub}\rightarrow\pi^*_{phen}$
				0.387	HOMO-1	LUMO+1	$\pi_{phen},\pi_{sub}\rightarrow\pi^*_{phen}$
6	33472.7	298.8	0.020	0.285	HOMO-4	LUMO	$\pi_{phen},(\pi_{sub})\rightarrow\pi^*_{phen}$
				0.414	HOMO-2	LUMO	$\pi_{phen},(\pi_{sub})\rightarrow\pi^*_{phen}$
				0.125	HOMO-1	LUMO+1	$\pi_{phen},\pi_{sub}\rightarrow\pi^*_{phen}$
7	34137.1	292.9	0.056	0.150	HOMO-3	LUMO	$\pi_{sub}\rightarrow\pi^*_{phen}$
				0.776	HOMO-2	LUMO+1	$\pi_{phen},(\pi_{sub})\rightarrow\pi^*_{phen}$
9	36460.2	274.3	0.179	0.496	HOMO-3	LUMO+1	$\pi_{sub}\rightarrow\pi^*_{phen}$
				0.172	HOMO	LUMO+2	$\pi_{sub},(\pi_{phen})\rightarrow\pi^*_{phen},(\pi^*_{sub})$
10	37113.8	269.4	0.348	0.491	HOMO-5	LUMO	$\pi_{sub},(\pi_{phen})\rightarrow\pi^*_{phen}$
11	37321.1	267.9	0.159	0.201	HOMO-5	LUMO	$\pi_{sub},(\pi_{phen})\rightarrow\pi^*_{phen}$
				0.417	HOMO-3	LUMO	$\pi_{sub}\rightarrow\pi^*_{phen}$
12	37493.1	266.7	0.289	0.145	HOMO-6	LUMO	$\pi_{phen},\pi_{sub}\rightarrow\pi^*_{phen}$
				0.264	HOMO-5	LUMO+1	$\pi_{sub},(\pi_{phen})\rightarrow\pi^*_{phen}$
				0.141	HOMO-3	LUMO	$\pi_{sub}\rightarrow\pi^*_{phen}$
				0.126	HOMO	LUMO+2	$\pi_{sub},(\pi_{phen})\rightarrow\pi^*_{phen},(\pi^*_{sub})$
14	38392.4	260.5	0.042	0.302	HOMO-6	LUMO	$\pi_{phen},\pi_{sub}\rightarrow\pi^*_{phen}$
				0.160	HOMO-6	LUMO+1	$\pi_{phen},\pi_{sub}\rightarrow\pi^*_{phen}$
				0.109	HOMO-5	LUMO+1	$\pi_{sub},(\pi_{phen})\rightarrow\pi^*_{phen}$
17	39767.6	251.5	0.137	0.477	HOMO-1	LUMO+2	$\pi_{phen},\pi_{sub}\rightarrow\pi^*_{phen},(\pi^*_{sub})$
				0.157	HOMO	LUMO+2	$\pi_{sub},(\pi_{phen})\rightarrow\pi^*_{phen},(\pi^*_{sub})$
18	40131.7	249.2	0.058	0.178	HOMO-2	LUMO+2	$\pi_{phen},(\pi_{sub})\rightarrow\pi^*_{phen},(\pi^*_{sub})$
				0.156	HOMO-1	LUMO+2	$\pi_{phen},\pi_{sub}\rightarrow\pi^*_{phen},(\pi^*_{sub})$
				0.192	HOMO	LUMO+2	$\pi_{sub},(\pi_{phen})\rightarrow\pi^*_{phen},(\pi^*_{sub})$
19	40846.8	244.8	0.137	0.117	HOMO-1	LUMO+2	$\pi_{phen},\pi_{sub}\rightarrow\pi^*_{phen},(\pi^*_{sub})$
				0.370	HOMO-1	LUMO+3	$\pi_{phen},\pi_{sub}\rightarrow\pi^*_{sub}$
				0.134	HOMO	LUMO+2	$\pi_{sub},(\pi_{phen})\rightarrow\pi^*_{phen},(\pi^*_{sub})$
20	41120.8	243.2	0.254	0.540	HOMO	LUMO+3	$\pi_{sub},(\pi_{phen})\rightarrow\pi^*_{sub}$
21	41585.4	240.5	0.012	0.223	HOMO-2	LUMO+3	$\pi_{phen},(\pi_{sub})\rightarrow\pi^*_{sub}$
				0.276	HOMO	LUMO+4	$\pi_{sub},(\pi_{phen})\rightarrow\pi^*_{sub}$
22	42449.4	235.6	0.072	0.493	HOMO-2	LUMO+2	$\pi_{phen},(\pi_{sub})\rightarrow\pi^*_{phen},(\pi^*_{sub})$
				0.192	HOMO-1	LUMO+3	$\pi_{phen},\pi_{sub}\rightarrow\pi^*_{sub}$
23	42865.6	233.3	0.051	0.149	HOMO-2	LUMO+3	$\pi_{phen},(\pi_{sub})\rightarrow\pi^*_{sub}$
				0.163	HOMO-1	LUMO+3	$\pi_{phen},\pi_{sub}\rightarrow\pi^*_{sub}$
				0.234	HOMO	LUMO+4	$\pi_{sub},(\pi_{phen})\rightarrow\pi^*_{sub}$
				0.116	HOMO	LUMO+5	$\pi_{sub},(\pi_{phen})\rightarrow\pi^*_{sub},\pi^*_{phen}$
24	43371.1	230.6	0.077	0.245	HOMO-4	LUMO+2	$\pi_{phen},(\pi_{sub})\rightarrow\pi^*_{phen},(\pi^*_{sub})$
				0.245	HOMO-2	LUMO+3	$\pi_{phen},(\pi_{sub})\rightarrow\pi^*_{sub}$
				0.158	HOMO	LUMO+5	$\pi_{sub},(\pi_{phen})\rightarrow\pi^*_{sub},\pi^*_{phen}$
25	43424.2	230.3	0.090	0.313	HOMO-4	LUMO+2	$\pi_{phen},(\pi_{sub})\rightarrow\pi^*_{phen},(\pi^*_{sub})$
				0.121	HOMO-2	LUMO+3	$\pi_{phen},(\pi_{sub})\rightarrow\pi^*_{sub}$
				0.269	HOMO	LUMO+5	$\pi_{sub},(\pi_{phen})\rightarrow\pi^*_{sub},\pi^*_{phen}$
26	43949.2	227.5	0.199	0.181	HOMO-2	LUMO+4	$\pi_{phen},(\pi_{sub})\rightarrow\pi^*_{sub}$
				0.229	HOMO-1	LUMO+4	$\pi_{phen},\pi_{sub}\rightarrow\pi^*_{sub}$
				0.101	HOMO	LUMO+4	$\pi_{sub},(\pi_{phen})\rightarrow\pi^*_{sub}$
27	44348.2	225.5	0.256	0.205	HOMO-9	LUMO	$\pi_{sub}\rightarrow\pi^*_{phen}$
				0.143	HOMO-3	LUMO+2	$\pi_{sub}\rightarrow\pi^*_{phen},(\pi^*_{sub})$
				0.174	HOMO-3	LUMO+3	$\pi_{sub}\rightarrow\pi^*_{sub}$
				0.105	HOMO	LUMO+5	$\pi_{sub},(\pi_{phen})\rightarrow\pi^*_{sub},\pi^*_{phen}$
28	44401.6	225.2	0.016	0.488	HOMO-8	LUMO	$\pi_{sub}\rightarrow\pi^*_{phen}$
				0.204	HOMO-8	LUMO+1	$\pi_{sub}\rightarrow\pi^*_{phen}$
29	44558.7	224.4	0.026	0.348	HOMO-9	LUMO	$\pi_{sub}\rightarrow\pi^*_{phen}$
				0.133	HOMO-8	LUMO+1	$\pi_{sub}\rightarrow\pi^*_{phen}$

				0.144	HOMO-1	LUMO+5	$\pi_{phen},\pi_{sub}\rightarrow\pi^*_{sub},\pi^*_{phen}$
30	44678.2	223.8	0.163	0.204	HOMO-9	LUMO+1	$\pi_{sub}\rightarrow\pi^*_{phen}$
				0.111	HOMO-8	LUMO	$\pi_{sub}\rightarrow\pi^*_{phen}$
				0.214	HOMO-3	LUMO+2	$\pi_{sub}\rightarrow\pi^*_{phen},(\pi^*_{sub})$

Table S7.2. Excitation energies, transition moments and corresponding transitions of **L1** obtained from TDDFT simulated in acetonitrile. Note that only excitations with an oscillator strength > 0.01 and corresponding orbital contributions with an OC ≥ 0.1 ($|coeff.|^2 100 \geq 0.1$) are shown. Molecular fragments that show only minor contribution to the mixing of orbitals are given in parenthesis (*e.g.*(π_{phen})).

State	Exc. energy		Oscillator	Dominant contribution			Transition
#	cm^{-1}	nm	strength	OC	occ. orb.	virt. orb.	
1	29574	338.1	0.073	0.879	HOMO	LUMO	$\pi_{sub},(\pi_{phen})\rightarrow\pi^*_{phen}$
2	30140.8	331.8	0.346	0.153	HOMO-1	LUMO	$\pi_{sub}\rightarrow\pi^*_{phen}$
				0.806	HOMO	LUMO+1	$\pi_{sub},(\pi_{phen})\rightarrow\pi^*_{phen}$
3	31174.7	320.8	0.029	0.677	HOMO-1	LUMO	$\pi_{sub}\rightarrow\pi^*_{phen}$
				0.125	HOMO-1	LUMO+1	$\pi_{sub}\rightarrow\pi^*_{phen}$
				0.127	HOMO	LUMO+1	$\pi_{sub},(\pi_{phen})\rightarrow\pi^*_{phen}$
4	31342.6	319.1	0.091	0.134	HOMO-1	LUMO	$\pi_{sub}\rightarrow\pi^*_{phen}$
				0.733	HOMO-1	LUMO+1	$\pi_{sub}\rightarrow\pi^*_{phen}$
7	34323.6	291.3	0.038	0.212	HOMO-3	LUMO	$\pi_{phen},(\pi_{sub})\rightarrow\pi^*_{phen}$
				0.704	HOMO-2	LUMO+1	$\pi_{phen},(\pi_{sub})\rightarrow\pi^*_{phen}$
9	36440.4	274.4	0.091	0.131	HOMO-5	LUMO	$\pi_{sub},(\pi_{phen})\rightarrow\pi^*_{phen}$
				0.401	HOMO-3	LUMO+1	$\pi_{phen},(\pi_{sub})\rightarrow\pi^*_{phen}$
				0.120	HOMO	LUMO+2	$\pi_{sub},(\pi_{phen})\rightarrow\pi^*_{phen},(\pi^*_{sub})$
10	36586.7	273.3	0.079	0.136	HOMO-6	LUMO	$\pi_{phen},\pi_{sub}\rightarrow\pi^*_{phen}$
				0.162	HOMO-5	LUMO	$\pi_{phen},\pi_{sub}\rightarrow\pi^*_{phen}$
				0.370	HOMO-4	LUMO	$\pi_{sub},(\pi_{phen})\rightarrow\pi^*_{phen}$
11	37089.4	269.6	0.080	0.157	HOMO-5	LUMO	$\pi_{phen},\pi_{sub}\rightarrow\pi^*_{phen}$
				0.103	HOMO-5	LUMO+1	$\pi_{phen},\pi_{sub}\rightarrow\pi^*_{phen}$
				0.195	HOMO-3	LUMO	$\pi_{phen},(\pi_{sub})\rightarrow\pi^*_{phen}$
				0.197	HOMO	LUMO+2	$\pi_{sub},(\pi_{phen})\rightarrow\pi^*_{phen},(\pi^*_{sub})$
12	37480.1	266.8	0.532	0.104	HOMO-6	LUMO+1	$\pi_{phen},\pi_{sub}\rightarrow\pi^*_{phen}$
				0.174	HOMO-5	LUMO+1	$\pi_{phen},\pi_{sub}\rightarrow\pi^*_{phen}$
				0.367	HOMO-3	LUMO	$\pi_{phen},(\pi_{sub})\rightarrow\pi^*_{phen}$
13	37993.2	263.2	0.042	0.384	HOMO-7	LUMO	$\pi_{phen}\rightarrow\pi^*_{phen}$
				0.107	HOMO-6	LUMO+1	$\pi_{phen},\pi_{sub}\rightarrow\pi^*_{phen}$
				0.235	HOMO-4	LUMO+1	$\pi_{sub},(\pi_{phen})\rightarrow\pi^*_{phen}$
14	38151.9	262.1	0.139	0.259	HOMO-6	LUMO	$\pi_{phen},\pi_{sub}\rightarrow\pi^*_{phen}$
				0.114	HOMO-5	LUMO	$\pi_{phen},\pi_{sub}\rightarrow\pi^*_{phen}$
				0.121	HOMO-5	LUMO+1	$\pi_{phen},\pi_{sub}\rightarrow\pi^*_{phen}$
15	38279.2	261.2	0.015	0.482	HOMO-7	LUMO	$\pi_{phen}\rightarrow\pi^*_{phen}$
				0.149	HOMO-6	LUMO+1	$\pi_{phen},\pi_{sub}\rightarrow\pi^*_{phen}$
				0.154	HOMO-4	LUMO+1	$\pi_{sub},(\pi_{phen})\rightarrow\pi^*_{phen}$
17	39410.7	253.7	0.164	0.560	HOMO-1	LUMO+2	$\pi_{sub}\rightarrow\pi^*_{phen},(\pi^*_{sub})$
				0.138	HOMO	LUMO+2	$\pi_{sub},(\pi_{phen})\rightarrow\pi^*_{phen},(\pi^*_{sub})$
18	39752.7	251.6	0.017	0.130	HOMO-5	LUMO+1	$\pi_{phen},\pi_{sub}\rightarrow\pi^*_{phen}$
				0.103	HOMO-2	LUMO+2	$\pi_{phen},(\pi_{sub})\rightarrow\pi^*_{phen},(\pi^*_{sub})$
				0.192	HOMO-1	LUMO+2	$\pi_{sub}\rightarrow\pi^*_{phen},(\pi^*_{sub})$
				0.297	HOMO	LUMO+2	$\pi_{sub},(\pi_{phen})\rightarrow\pi^*_{phen},(\pi^*_{sub})$
19	40174.3	248.9	0.214	0.237	HOMO-1	LUMO+3	$\pi_{sub}\rightarrow\pi^*_{sub},(\pi^*_{phen})$
				0.128	HOMO	LUMO+3	$\pi_{sub},(\pi_{phen})\rightarrow\pi^*_{sub},(\pi^*_{phen})$
				0.173	HOMO	LUMO+4	$\pi_{sub},(\pi_{phen})\rightarrow\pi^*_{sub}$
20	40578.8	246.4	0.129	0.107	HOMO-1	LUMO+2	$\pi_{sub}\rightarrow\pi^*_{phen},(\pi^*_{sub})$
				0.140	HOMO	LUMO+3	$\pi_{sub},(\pi_{phen})\rightarrow\pi^*_{sub},(\pi^*_{phen})$
				0.318	HOMO	LUMO+4	$\pi_{sub},(\pi_{phen})\rightarrow\pi^*_{sub}$
21	40993	243.9	0.121	0.359	HOMO-1	LUMO+3	$\pi_{sub}\rightarrow\pi^*_{sub},(\pi^*_{phen})$
				0.320	HOMO	LUMO+3	$\pi_{sub},(\pi_{phen})\rightarrow\pi^*_{sub},(\pi^*_{phen})$
23	42639.7	234.5	0.026	0.181	HOMO-2	LUMO+2	$\pi_{phen},(\pi_{sub})\rightarrow\pi^*_{phen},(\pi^*_{sub})$
				0.448	HOMO-1	LUMO+4	$\pi_{sub}\rightarrow\pi^*_{sub}$
				0.171	HOMO	LUMO+4	$\pi_{sub},(\pi_{phen})\rightarrow\pi^*_{sub}$
24	43033.2	232.4	0.024	0.140	HOMO-2	LUMO+2	$\pi_{phen},(\pi_{sub})\rightarrow\pi^*_{phen},(\pi^*_{sub})$
				0.227	HOMO-2	LUMO+3	$\pi_{phen},(\pi_{sub})\rightarrow\pi^*_{sub},(\pi^*_{phen})$
				0.384	HOMO	LUMO+5	$\pi_{sub},(\pi_{phen})\rightarrow\pi^*_{sub},(\pi^*_{phen})$
25	43554.4	229.6	0.243	0.191	HOMO-2	LUMO+2	$\pi_{phen},(\pi_{sub})\rightarrow\pi^*_{phen},(\pi^*_{sub})$

				0.383	HOMO-2	LUMO+3	$\pi_{phen},(\pi_{sub})\rightarrow \pi^*_{sub},(\pi^*_{phen})$
				0.117	HOMO-1	LUMO+4	$\pi_{sub}\rightarrow\pi^*_{sub}$
26	43653.5	229.1	0.021	0.114	HOMO-7	LUMO+1	$\pi_{phen}\rightarrow\pi^*_{phen}$
				0.296	HOMO-6	LUMO+2	$\pi_{phen},\pi_{sub}\rightarrow\pi^*_{phen},(\pi^*_{sub})$
				0.155	HOMO-5	LUMO+2	$\pi_{phen},\pi_{sub}\rightarrow\pi^*_{phen},(\pi^*_{sub})$
				0.305	HOMO-4	LUMO+2	$\pi_{sub},(\pi_{phen})\rightarrow\pi^*_{phen},(\pi^*_{sub})$
27	44043.8	227	0.122	0.169	HOMO-2	LUMO+3	$\pi_{phen},(\pi_{sub})\rightarrow \pi^*_{sub},(\pi^*_{phen})$
				0.556	HOMO-1	LUMO+5	$\pi_{sub}\rightarrow\pi^*_{sub},(\pi^*_{phen})$
28	44612.9	224.2	0.307	0.140	HOMO-8	LUMO	$\pi_{phen}\rightarrow\pi^*_{phen}$
				0.357	HOMO-3	LUMO+2	$\pi_{phen},(\pi_{sub})\rightarrow\pi^*_{phen},(\pi^*_{sub})$
				0.209	HOMO-2	LUMO+4	$\pi_{phen},(\pi_{sub})\rightarrow\pi^*_{sub}$
29	44949.2	222.5	0.180	0.128	HOMO-3	LUMO+2	$\pi_{phen},(\pi_{sub})\rightarrow\pi^*_{phen},(\pi^*_{sub})$
				0.490	HOMO-2	LUMO+4	$\pi_{phen},(\pi_{sub})\rightarrow\pi^*_{sub}$

Table S7.3. Excitation energies, transition moments and corresponding transitions of **C1'** obtained from TDDFT simulated in acetonitrile. Note that only excitations with an oscillator strength > 0.01 and corresponding orbital contributions with an OC ≥ 0.1 ($|\text{coeff.}|^2 100 \geq 0.1$) are shown. Molecular fragments that show only minor contribution to the mixing of orbitals are given in parenthesis (*e.g.*(π_{phen})).

State	Exc. energy		Oscillator	Dominant contribution			Transition
#	cm^{-1}	nm	strength	OC	occ. orb.	virt. orb.	
1	18220.7	548.8	0.030	0.169	HOMO-1	LUMO+1	$d_{Cu}\rightarrow\pi^*_{phen},(d_{Cu})$
				0.613	HOMO	LUMO	$d_{Cu}\rightarrow\pi^*_{phen},(d_{Cu})$
				0.149	HOMO	LUMO+1	$d_{Cu}\rightarrow\pi^*_{phen},(d_{Cu})$
3	20198.3	495.1	0.401	0.155	HOMO-1	LUMO	$d_{Cu}\rightarrow\pi^*_{phen},(d_{Cu})$
				0.605	HOMO-1	LUMO+1	$d_{Cu}\rightarrow\pi^*_{phen},(d_{Cu})$
				0.177	HOMO	LUMO	$d_{Cu}\rightarrow\pi^*_{phen},(d_{Cu})$
4	21050	475.1	0.015	0.820	HOMO	LUMO+2	$d_{Cu}\rightarrow\pi^*_{phen}$
5	21170.5	472.4	0.010	0.802	HOMO	LUMO+3	$d_{Cu}\rightarrow\pi^*_{phen}$
6	22602	442.4	0.068	0.754	HOMO-1	LUMO+2	$d_{Cu}\rightarrow\pi^*_{phen}$
				0.120	HOMO-1	LUMO+3	$d_{Cu}\rightarrow\pi^*_{phen}$
7	22716.5	440.2	0.053	0.125	HOMO-1	LUMO+2	$d_{Cu}\rightarrow\pi^*_{phen}$
				0.786	HOMO-1	LUMO+3	$d_{Cu}\rightarrow\pi^*_{phen}$
17	28805.3	347.2	0.015	0.251	HOMO-5	LUMO	$d_{Cu},\pi_{sub}\rightarrow\pi^*_{phen},(d_{Cu})$
				0.263	HOMO-2	LUMO	$d_{Cu},\pi_{sub}\rightarrow\pi^*_{phen},(d_{Cu})$
18	28869.8	346.4	0.012	0.205	HOMO-5	LUMO	$d_{Cu},\pi_{sub}\rightarrow\pi^*_{phen},(d_{Cu})$
				0.294	HOMO-4	LUMO+1	$d_{Cu},\pi_{sub}\rightarrow\pi^*_{phen},(d_{Cu})$
				0.257	HOMO-2	LUMO+1	$d_{Cu},\pi_{sub}\rightarrow\pi^*_{phen},(d_{Cu})$
19	29095.3	343.7	0.259	0.120	HOMO-2	LUMO+2	$d_{Cu},\pi_{sub}\rightarrow\pi^*_{phen}$
				0.325	HOMO-2	LUMO+3	$d_{Cu},\pi_{sub}\rightarrow\pi^*_{phen}$
20	29218.2	342.3	0.136	0.107	HOMO-7	LUMO+1	$d_{Cu}\rightarrow\pi^*_{phen},(d_{Cu})$
				0.273	HOMO-6	LUMO	$\pi_{sub},(d_{Cu})\rightarrow\pi^*_{phen},(d_{Cu})$
21	29251.1	341.9	0.218	0.247	HOMO-6	LUMO+1	$\pi_{sub},(d_{Cu})\rightarrow\pi^*_{phen},(d_{Cu})$
				0.102	HOMO-4	LUMO	$d_{Cu},\pi_{sub}\rightarrow\pi^*_{phen},(d_{Cu})$
				0.127	HOMO-4	LUMO+3	$d_{Cu},\pi_{sub}\rightarrow\pi^*_{phen}$
23	29887.5	334.6	0.012	0.690	HOMO	LUMO+5	$d_{Cu}\rightarrow\pi^*_{phen}$
24	29896.2	334.5	0.025	0.107	HOMO-8	LUMO+2	$d_{Cu}\rightarrow\pi^*_{phen}$
				0.162	HOMO-2	LUMO+2	$d_{Cu},\pi_{sub}\rightarrow\pi^*_{phen}$
				0.141	HOMO	LUMO+4	$d_{Cu}\rightarrow\pi^*_{phen}$
25	30016	333.2	0.035	0.392	HOMO-5	LUMO+1	$d_{Cu},\pi_{sub}\rightarrow\pi^*_{phen},(d_{Cu})$
				0.163	HOMO-4	LUMO+3	$d_{Cu},\pi_{sub}\rightarrow\pi^*_{phen}$
				0.141	HOMO-2	LUMO+1	$d_{Cu},\pi_{sub}\rightarrow\pi^*_{phen},(d_{Cu})$
26	30062.3	332.6	0.038	0.166	HOMO-5	LUMO+2	$d_{Cu},\pi_{sub}\rightarrow\pi^*_{phen}$
				0.364	HOMO-4	LUMO	$d_{Cu},\pi_{sub}\rightarrow\pi^*_{phen},(d_{Cu})$
27	30092.6	332.3	0.050	0.179	HOMO-5	LUMO+2	$d_{Cu},\pi_{sub}\rightarrow\pi^*_{phen}$
				0.122	HOMO-2	LUMO	$d_{Cu},\pi_{sub}\rightarrow\pi^*_{phen},(d_{Cu})$
28	30254	330.5	0.020	0.274	HOMO-8	LUMO+2	$d_{Cu}\rightarrow\pi^*_{phen}$
				0.135	HOMO-4	LUMO+3	$d_{Cu},\pi_{sub}\rightarrow\pi^*_{phen}$
30	30447.7	328.4	0.014	0.671	HOMO-8	LUMO+3	$d_{Cu}\rightarrow\pi^*_{phen}$
				0.116	HOMO-7	LUMO+3	$d_{Cu}\rightarrow\pi^*_{phen}$

Table S7.4. Excitation energies, transition moments and corresponding transitions of **C1** obtained from TDDFT simulated in acetonitrile. Note that only excitations with an oscillator strength > 0.01 and corresponding orbital contributions with an OC ≥ 0.1 ($|\text{coeff.}|^2 100 \geq 0.1$) are shown. Molecular fragments that show only minor contribution to the mixing of orbitals are given in parenthesis (*e.g.*(π_{phen})).

State #	Exc. energy cm^{-1}	nm	Oscillator strength	Dominant contribution OC	occ. orb.	virt. orb.	Transition
1	18029	554.7	0.112	0.888	HOMO	LUMO	$d_{Cu}\rightarrow\pi^*_{phen},(d_{Cu})$
3	20706.5	482.9	0.039	0.854	HOMO	LUMO+2	$d_{Cu}\rightarrow\pi^*_{phen}$
4	20745.6	482	0.298	0.757	HOMO-1	LUMO+1	$d_{Cu}\rightarrow\pi^*_{phen},(d_{Cu})$
				0.126	HOMO	LUMO+3	$d_{Cu}\rightarrow\pi^*_{phen}$
5	20757.1	481.8	0.072	0.120	HOMO-1	LUMO+1	$d_{Cu}\rightarrow\pi^*_{phen},(d_{Cu})$
				0.732	HOMO	LUMO+3	$d_{Cu}\rightarrow\pi^*_{phen}$
6	23238.9	430.3	0.039	0.892	HOMO-1	LUMO+2	$d_{Cu}\rightarrow\pi^*_{phen}$
7	23284.7	429.5	0.062	0.886	HOMO-1	LUMO+3	$d_{Cu}\rightarrow\pi^*_{phen}$
15	28059.5	356.4	0.043	0.115	HOMO-6	LUMO+3	$d_{Cu}\rightarrow\pi^*_{phen}$
				0.423	HOMO-3	LUMO	$\pi_{sub},(d_{Cu})\rightarrow\pi^*_{phen},(d_{Cu})$
				0.212	HOMO-2	LUMO+1	$\pi_{sub},(d_{Cu})\rightarrow\pi^*_{phen},(d_{Cu})$
17	28178	354.9	0.031	0.151	HOMO-6	LUMO+2	$d_{Cu}\rightarrow\pi^*_{phen}$
				0.663	HOMO-6	LUMO+3	$d_{Cu}\rightarrow\pi^*_{phen}$
19	28454.6	351.4	0.137	0.355	HOMO-5	LUMO	$\pi_{sub},(d_{Cu})\rightarrow\pi^*_{phen},(d_{Cu})$
				0.140	HOMO-3	LUMO+3	$\pi_{sub},(d_{Cu})\rightarrow\pi^*_{phen}$
				0.192	HOMO-2	LUMO+2	$\pi_{sub},(d_{Cu})\rightarrow\pi^*_{phen}$
20	28509.2	350.8	0.340	0.124	HOMO-7	LUMO	$d_{Cu}\rightarrow\pi^*_{phen},(d_{Cu})$
				0.259	HOMO-5	LUMO+1	$\pi_{sub},(d_{Cu})\rightarrow\pi^*_{phen},(d_{Cu})$
				0.118	HOMO-4	LUMO	$\pi_{sub},(d_{Cu})\rightarrow\pi^*_{phen},(d_{Cu})$
				0.176	HOMO-3	LUMO+2	$\pi_{sub},(d_{Cu})\rightarrow\pi^*_{phen}$
				0.168	HOMO-2	LUMO+3	$\pi_{sub},(d_{Cu})\rightarrow\pi^*_{phen}$
21	28681.3	348.7	0.376	0.111	HOMO-7	LUMO+2	$d_{Cu}\rightarrow\pi^*_{phen}$
				0.400	HOMO-4	LUMO+2	$\pi_{sub},(d_{Cu})\rightarrow\pi^*_{phen}$
				0.197	HOMO-2	LUMO+2	$\pi_{sub},(d_{Cu})\rightarrow\pi^*_{phen}$
23	29075.6	343.9	0.037	0.106	HOMO-4	LUMO+1	$\pi_{sub},(d_{Cu})\rightarrow\pi^*_{phen},(d_{Cu})$
				0.113	HOMO-4	LUMO+2	$\pi_{sub},(d_{Cu})\rightarrow\pi^*_{phen}$
				0.192	HOMO-3	LUMO	$\pi_{sub},(d_{Cu})\rightarrow\pi^*_{phen},(d_{Cu})$
				0.115	HOMO-3	LUMO+3	$\pi_{sub},(d_{Cu})\rightarrow\pi^*_{phen}$
				0.106	HOMO-2	LUMO+2	$\pi_{sub},(d_{Cu})\rightarrow\pi^*_{phen}$
24	29140.6	343.2	0.022	0.136	HOMO-5	LUMO	$\pi_{sub},(d_{Cu})\rightarrow\pi^*_{phen},(d_{Cu})$
				0.160	HOMO-3	LUMO+1	$\pi_{sub},(d_{Cu})\rightarrow\pi^*_{phen},(d_{Cu})$
				0.167	HOMO-2	LUMO+1	$\pi_{sub},(d_{Cu})\rightarrow\pi^*_{phen},(d_{Cu})$
25	29144.3	343.1	0.033	0.117	HOMO-3	LUMO	$\pi_{sub},(d_{Cu})\rightarrow\pi^*_{phen},(d_{Cu})$
				0.222	HOMO-3	LUMO+1	$\pi_{sub},(d_{Cu})\rightarrow\pi^*_{phen},(d_{Cu})$
				0.132	HOMO-3	LUMO+2	$\pi_{sub},(d_{Cu})\rightarrow\pi^*_{phen}$
				0.135	HOMO-2	LUMO+1	$\pi_{sub},(d_{Cu})\rightarrow\pi^*_{phen},(d_{Cu})$
26	29195.4	342.5	0.021	0.176	HOMO-5	LUMO+1	$\pi_{sub},(d_{Cu})\rightarrow\pi^*_{phen},(d_{Cu})$
				0.104	HOMO-4	LUMO+3	$\pi_{sub},(d_{Cu})\rightarrow\pi^*_{sub}$
				0.241	HOMO-3	LUMO+1	$\pi_{sub},(d_{Cu})\rightarrow\pi^*_{phen},(d_{Cu})$
				0.156	HOMO-2	LUMO	$\pi_{sub},(d_{Cu})\rightarrow\pi^*_{phen},(d_{Cu})$
27	29599.1	337.8	0.034	0.878	HOMO	LUMO+5	$d_{Cu}\rightarrow\pi^*_{sub}$

Table S7.5. Excitation energies, transition moments and corresponding transitions of **C2'** obtained from TDDFT simulated in acetonitrile. Note that only excitations with an oscillator strength > 0.01 and corresponding orbital contributions with an OC ≥ 0.1 ($|\text{coeff.}|^2 100 \geq 0.1$) are shown. Molecular fragments that show only minor contribution to the mixing of orbitals are given in parenthesis (*e.g.*(π_{phen})).

State #	Exc. energy cm^{-1}	nm	Oscillator strength	Dominant contribution OC	occ. orb.	virt. orb.	Transition
1	23140.3	432.1	0.141	0.948	HOMO	LUMO	$d_{Cu}\rightarrow\pi^*_{phen}$
2	24762.5	403.8	0.057	0.939	HOMO	LUMO+1	$d_{Cu}\rightarrow\pi^*_{phen}$
3	25211.9	396.6	0.022	0.827	HOMO-1	LUMO	$d_{Cu}\rightarrow\pi^*_{phen}$
4	27010.4	370.2	0.020	0.128	HOMO-4	LUMO	$d_{Cu},\pi_{sub}\rightarrow\pi^*_{phen}$
				0.110	HOMO-3	LUMO	$d_{Cu},\pi_{sub}\rightarrow\pi^*_{phen}$
				0.654	HOMO-2	LUMO	$d_{Cu},(\pi_{sub})\rightarrow\pi^*_{phen}$
5	27758.2	360.3	0.024	0.860	HOMO-1	LUMO+1	$d_{Cu}\rightarrow\pi^*_{phen}$

6	29098.2	343.7	0.094	0.469	HOMO-3	LUMO	$d_{Cu},\pi_{sub}\rightarrow\pi^*_{phen}$
				0.230	HOMO-2	LUMO	$d_{Cu},(\pi_{sub})\rightarrow\pi^*_{phen}$
8	29326.7	341	0.022	0.684	HOMO-2	LUMO+1	$d_{Cu},(\pi_{sub})\rightarrow\pi^*_{phen}$
9	29699.6	336.7	0.046	0.552	HOMO-4	LUMO	$d_{Cu},\pi_{sub}\rightarrow\pi^*_{phen}$
				0.100	HOMO-4	LUMO+1	$d_{Cu},\pi_{sub}\rightarrow\pi^*_{phen}$
				0.223	HOMO-3	LUMO	$d_{Cu},\pi_{sub}\rightarrow\pi^*_{phen}$
10	29957.9	333.8	0.027	0.941	HOMO	LUMO+3	$d_{Cu}\rightarrow\pi^*_{xant}$
11	30518.1	327.7	0.060	0.134	HOMO-6	LUMO	$\pi_{phen},(d_{Cu})\rightarrow\pi^*_{phen}$
				0.532	HOMO-3	LUMO+1	$d_{Cu},\pi_{sub}\rightarrow\pi^*_{phen}$
				0.115	HOMO-2	LUMO+1	$d_{Cu},(\pi_{sub})\rightarrow\pi^*_{phen}$
12	30708.3	325.6	0.084	0.708	HOMO-4	LUMO+1	$d_{Cu},\pi_{sub}\rightarrow\pi^*_{phen}$
14	31532.7	317.1	0.015	0.826	HOMO	LUMO+4	$d_{Cu}\rightarrow\pi^*_{xant}$
15	31947.7	313	0.018	0.561	HOMO-6	LUMO	$\pi_{phen},(d_{Cu})\rightarrow\pi^*_{phen}$
				0.132	HOMO-3	LUMO+1	$d_{Cu},\pi_{sub}\rightarrow\pi^*_{phen}$
16	32307.5	309.5	0.039	0.553	HOMO	LUMO+5	$d_{Cu}\rightarrow\pi^*_{phen}$
				0.305	HOMO	LUMO+6	$d_{Cu}\rightarrow\pi^*_{phen}$
17	32614.8	306.6	0.160	0.293	HOMO	LUMO+5	$d_{Cu}\rightarrow\pi^*_{phen},\pi^*_{xant}$
				0.588	HOMO	LUMO+6	$d_{Cu}\rightarrow\pi^*_{xant}$
18	32978.1	303.2	0.023	0.134	HOMO-7	LUMO	$d_{Cu},\pi_{phen},\pi_{sub}\rightarrow\pi^*_{phen}$
				0.151	HOMO-6	LUMO+1	$\pi_{phen},(d_{Cu})\rightarrow\pi^*_{phen}$
				0.649	HOMO-5	LUMO+1	$\pi_{xant},(d_{Cu})\rightarrow\pi^*_{phen}$
19	33181.1	301.4	0.013	0.279	HOMO-7	LUMO	$d_{Cu},\pi_{phen},\pi_{sub}\rightarrow\pi^*_{phen}$
				0.186	HOMO-5	LUMO+1	$\pi_{xant},(d_{Cu})\rightarrow\pi^*_{phen}$
				0.112	HOMO-1	LUMO+2	$d_{Cu}\rightarrow\pi^*_{xant}$
21	33483.5	298.7	0.016	0.121	HOMO-6	LUMO+1	$\pi_{phen},(d_{Cu})\rightarrow\pi^*_{phen}$
				0.561	HOMO	LUMO+7	$d_{Cu}\rightarrow\pi^*_{xant}$
22	33519.1	298.3	0.026	0.237	HOMO-13	LUMO	$d_{Cu},(\pi_{xant})\rightarrow\pi^*_{phen}$
				0.118	HOMO	LUMO+7	$d_{Cu}\rightarrow\pi^*_{xant}$
23	33770.2	296.1	0.048	0.120	HOMO-14	LUMO	$d_{Cu},(\pi_{xant})\rightarrow\pi^*_{phen}$
				0.131	HOMO	LUMO+7	$d_{Cu}\rightarrow\pi^*_{xant}$
				0.399	HOMO	LUMO+8	$d_{Cu}\rightarrow\pi^*_{xant}$
24	33841.3	295.5	0.025	0.156	HOMO-14	LUMO	$d_{Cu},(\pi_{xant})\rightarrow\pi^*_{phen}$
				0.102	HOMO-6	LUMO+1	$\pi_{phen},(d_{Cu})\rightarrow\pi^*_{phen}$
				0.338	HOMO	LUMO+8	$d_{Cu}\rightarrow\pi^*_{xant}$
26	34215.9	292.3	0.051	0.144	HOMO-4	LUMO+2	$d_{Cu},\pi_{sub}\rightarrow\pi^*_{xant}$
				0.108	HOMO-3	LUMO+2	$d_{Cu},\pi_{sub}\rightarrow\pi^*_{xant}$
				0.381	HOMO-2	LUMO+2	$d_{Cu},(\pi_{sub})\rightarrow\pi^*_{xant}$
28	34589.8	289.1	0.148	0.129	HOMO-10	LUMO	$d_{Cu},\pi_{phen},\pi_{sub}\rightarrow\pi^*_{phen}$
				0.517	HOMO-8	LUMO	$\pi_{xant}\rightarrow\pi^*_{phen}$
29	34858	286.9	0.093	0.285	HOMO-2	LUMO+3	$d_{Cu},(\pi_{sub})\rightarrow\pi^*_{xant}$
30	34898.6	286.5	0.031	0.275	HOMO	LUMO+9	$d_{Cu}\rightarrow\pi^*_{phen},\pi^*_{sub},\pi^*_{xant}$
				0.365	HOMO	LUMO+10	$d_{Cu}\rightarrow\pi^*_{xant}$

Table S7.6. Excitation energies, transition moments and corresponding transitions of **C2** obtained from TDDFT simulated in acetonitrile. Note that only excitations with an oscillator strength > 0.01 and corresponding orbital contributions with an OC ≥ 0.1 ($|coeff.|^2 100 \geq 0.1$) are shown. Molecular fragments that show only minor contribution to the mixing of orbitals are given in parenthesis (*e.g.*(π_{phen})).

State	Exc. energy		Oscillator	Dominant contribution			Transition
#	cm^{-1}	nm	strength	OC	occ. orb.	virt. orb.	
1	23229.6	430.5	0.154	0.944	HOMO	LUMO	$d_{Cu}\rightarrow\pi^*_{phen}$
2	24845.7	402.5	0.074	0.933	HOMO	LUMO+1	$d_{Cu}\rightarrow\pi^*_{phen}$
3	25285.7	395.5	0.025	0.126	HOMO-4	LUMO	$d_{Cu}\rightarrow\pi^*_{phen}$
				0.334	HOMO-3	LUMO	$d_{Cu},(\pi_{sub})\rightarrow\pi^*_{phen}$
				0.467	HOMO-1	LUMO	$d_{Cu},\pi_{sub}\rightarrow\pi^*_{phen}$
4	27119.2	368.7	0.026	0.493	HOMO-4	LUMO	$d_{Cu}\rightarrow\pi^*_{phen}$
				0.304	HOMO-3	LUMO	$d_{Cu},(\pi_{sub})\rightarrow\pi^*_{phen}$
				0.108	HOMO-2	LUMO	$\pi_{sub},(d_{Cu})\rightarrow\pi^*_{phen}$
5	27738.9	360.5	0.067	0.203	HOMO-3	LUMO+1	$d_{Cu},(\pi_{sub})\rightarrow\pi^*_{phen}$
				0.606	HOMO-1	LUMO+1	$d_{Cu},\pi_{sub}\rightarrow\pi^*_{phen}$
6	28424.9	351.8	0.088	0.219	HOMO-4	LUMO	$d_{Cu}\rightarrow\pi^*_{phen}$
				0.270	HOMO-3	LUMO	$d_{Cu},(\pi_{sub})\rightarrow\pi^*_{phen}$
				0.385	HOMO-1	LUMO	$d_{Cu},\pi_{sub}\rightarrow\pi^*_{phen}$
7	28755.3	347.8	0.066	0.103	HOMO-4	LUMO	$d_{Cu}\rightarrow\pi^*_{phen}$
				0.716	HOMO-2	LUMO	$\pi_{sub},(d_{Cu})\rightarrow\pi^*_{phen}$

9	29296.1	341.3	0.047	0.141	HOMO-4	LUMO+1	$d_{Cu}\rightarrow\pi^*_{phen}$
				0.387	HOMO-3	LUMO+1	$d_{Cu},(\pi_{sub})\rightarrow\pi^*_{phen}$
				0.269	HOMO-2	LUMO+1	$\pi_{sub},(d_{Cu})\rightarrow\pi^*_{phen}$
10	29830.2	335.2	0.044	0.308	HOMO-4	LUMO+1	$d_{Cu}\rightarrow\pi^*_{phen}$
				0.152	HOMO-1	LUMO+1	$d_{Cu},\pi_{sub}\rightarrow\pi^*_{phen}$
				0.324	HOMO	LUMO+3	$d_{Cu}\rightarrow\pi^*_{xant}$
11	29865.8	334.8	0.088	0.177	HOMO-3	LUMO+1	$d_{Cu},(\pi_{sub})\rightarrow\pi^*_{phen}$
				0.506	HOMO-2	LUMO+1	$\pi_{sub},(d_{Cu})\rightarrow\pi^*_{phen}$
				0.197	HOMO	LUMO+3	$d_{Cu}\rightarrow\pi^*_{xant}$
12	29905.2	334.4	0.063	0.373	HOMO-4	LUMO+1	$d_{Cu}\rightarrow\pi^*_{phen}$
				0.425	HOMO	LUMO+3	$d_{Cu}\rightarrow\pi^*_{xant}$
14	31475.9	317.7	0.013	0.772	HOMO	LUMO+4	$d_{Cu}\rightarrow\pi^*_{xant}$
16	32253.4	310	0.042	0.608	HOMO	LUMO+5	$d_{Cu}\rightarrow\pi^*_{xant},(\pi^*_{phen})$
				0.201	HOMO	LUMO+6	$d_{Cu}\rightarrow\pi^*_{xant},(\pi^*_{phen})$
17	32638.8	306.4	0.161	0.153	HOMO	LUMO+5	$d_{Cu}\rightarrow\pi^*_{xant},(\pi^*_{phen})$
				0.716	HOMO	LUMO+6	$d_{Cu}\rightarrow\pi^*_{xant},(\pi^*_{phen})$
19	33261.2	300.7	0.014	0.216	HOMO-5	LUMO+1	$\pi_{xant},(d_{Cu})\rightarrow\pi^*_{phen}$
				0.281	HOMO-3	LUMO+2	$d_{Cu},(\pi_{sub})\rightarrow\pi^*_{xant}$
				0.191	HOMO-1	LUMO+2	$d_{Cu},\pi_{sub}\rightarrow\pi^*_{xant}$
21	33527	298.3	0.029	0.605	HOMO	LUMO+7	$d_{Cu}\rightarrow\pi^*_{xant},(\pi^*_{phen})$
22	33691.2	296.8	0.016	0.201	HOMO-13	LUMO	$d_{Cu}\rightarrow\pi^*_{phen}$
				0.153	HOMO-6	LUMO+1	$\pi_{phen},(d_{Cu})\rightarrow\pi^*_{phen}$
				0.177	HOMO	LUMO+8	$d_{Cu}\rightarrow\pi^*_{xant},\pi^*_{phen}$
23	33756.1	296.2	0.046	0.110	HOMO	LUMO+7	$d_{Cu}\rightarrow\pi^*_{xant},(\pi^*_{phen})$
				0.460	HOMO	LUMO+8	$d_{Cu}\rightarrow\pi^*_{xant},\pi^*_{phen}$
26	34138.1	292.9	0.040	0.430	HOMO-4	LUMO+2	$d_{Cu}\rightarrow\pi^*_{xant}$
28	34434.7	290.4	0.102	0.550	HOMO-8	LUMO	$\pi_{sub},(\pi_{phen}),(d_{Cu})\rightarrow\pi^*_{phen}$
29	34630.8	288.8	0.064	0.450	HOMO-9	LUMO	$\pi_{xant},(\pi_{sub}),(\pi_{phen})\rightarrow \pi^*_{phen}$
				0.124	HOMO-7	LUMO+1	$d_{Cu},\pi_{phen},\pi_{sub}\rightarrow\pi^*_{phen}$

8 UV/vis absorption

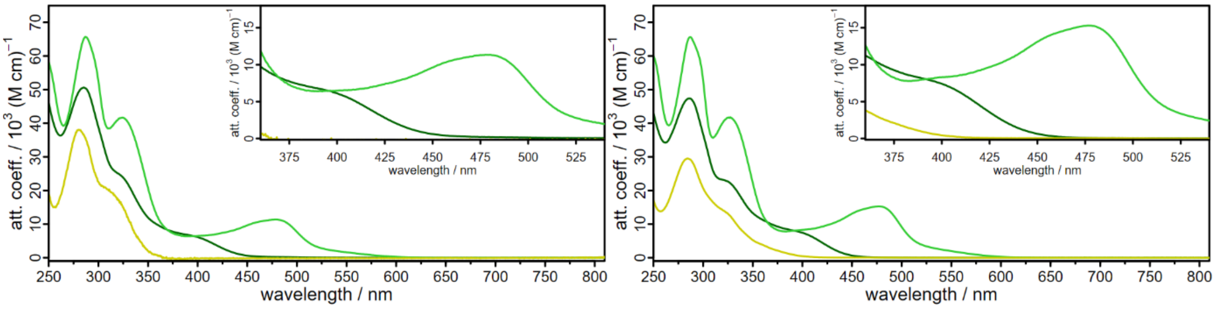

Figure S8.1. Experimental UV/vis absorption spectra of the methoxy protected dyes, **L1'** (yellow), **C1'** (green) and **C2'** (dark green) in acetonitrile (left) and methanol (right) under ambient conditions.

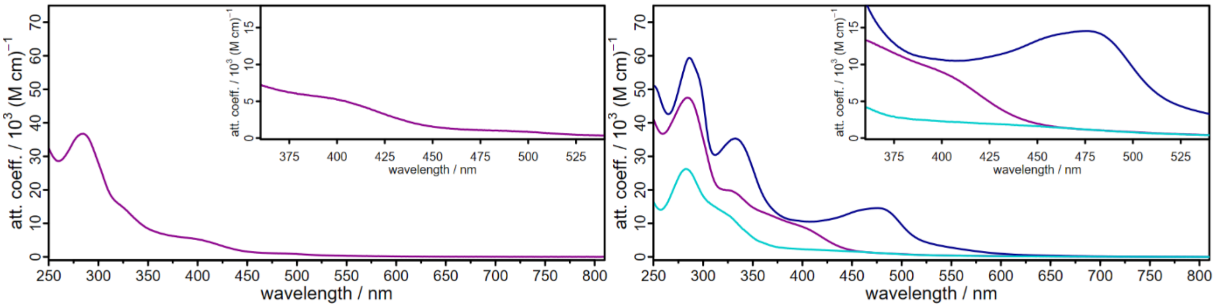

Figure S8.2. Experimental UV/vis absorption spectra of the deprotected dyes, **L1** (light blue), **C1** (dark blue) and **C2** (magenta) in acetonitrile (left) and methanol (right) under ambient conditions.

9 Steady-state emission

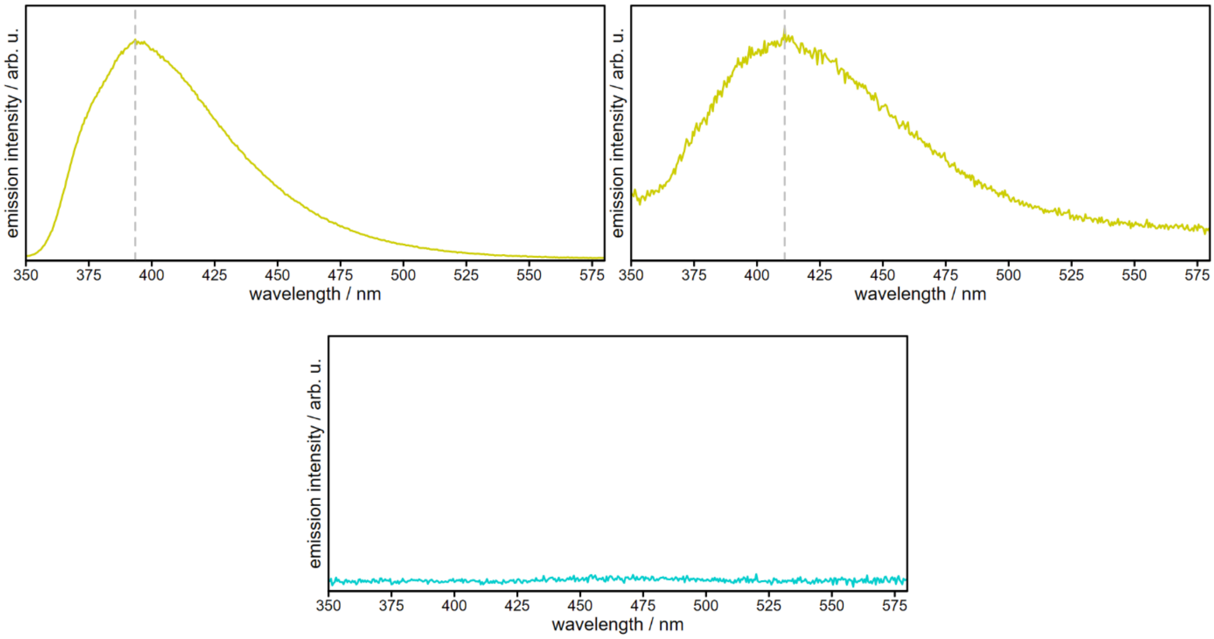

Figure S9.1. Steady-state emission spectra of the ligands **L1'** in acetonitrile (top-left, OD_{310} = 0.080) and in methanol (top-right, OD_{310} = 0.125) and **L1** in methanol (bottom-center, OD_{310} = 0.103) under inert conditions. Excitation wavelength was set to λ_{exc} = 310 nm. The grey dashed line denotes the wavelength of the emission maximum.

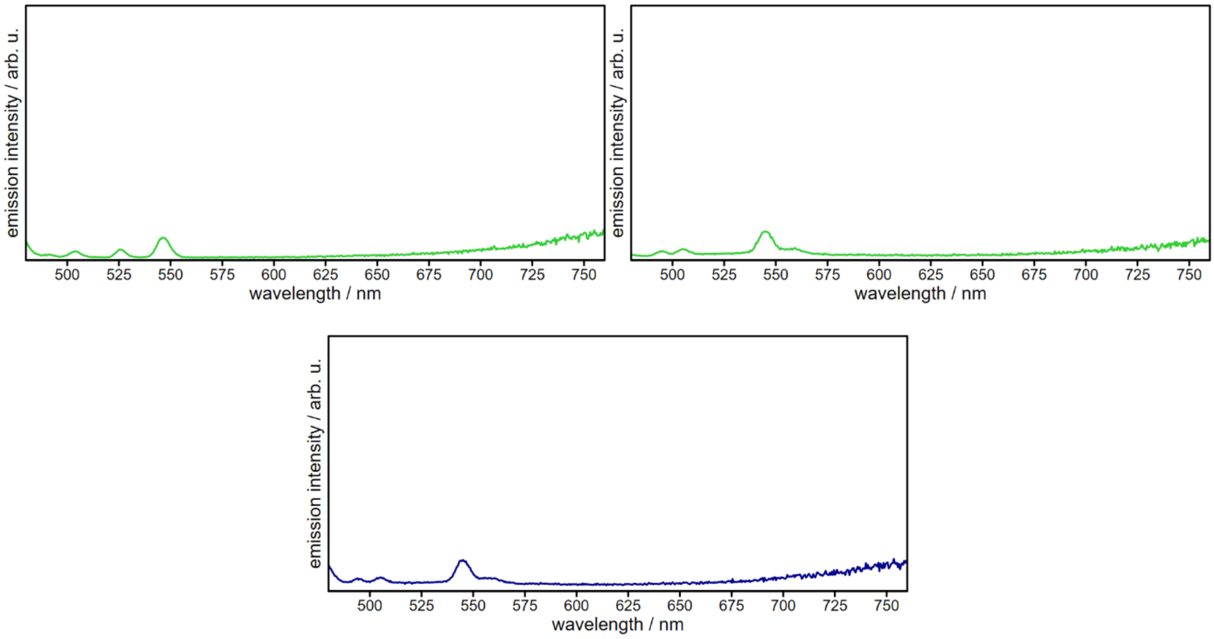

Figure S9.2. Steady-state emission spectra of the homoleptic complexes **C1'** in acetonitrile (top-left, OD_{470} = 0.091) and in methanol (top-right, OD_{470} = 0.092) and **C1** in methanol (bottom-center, OD_{470} = 0.095) under inert conditions. Excitation wavelength was set to λ_{exc} = 470 nm.

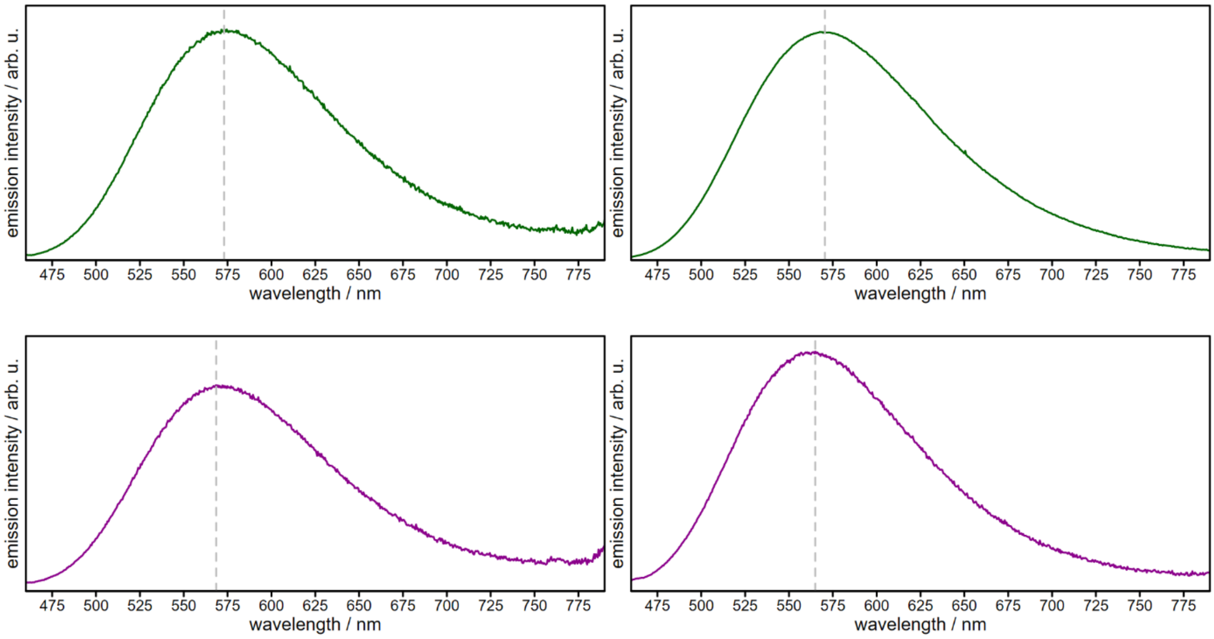

Figure S9.3. Steady-state emission spectra of the heteroleptic complexes **C2'** in acetonitrile (top-left, $OD_{400} = 0.058$) and in methanol (top-right, $OD_{400} = 0.063$) and **C2** in acetonitrile (bottom-left, $OD_{400} = 0.066$) and in methanol (bottom-right, $OD_{400} = 0.073$) under inert conditions. Excitation wavelength was set to λ_{exc} = 400 nm. The grey dashed line denotes the wavelength of the emission maximum.

10 Time-resolved emission

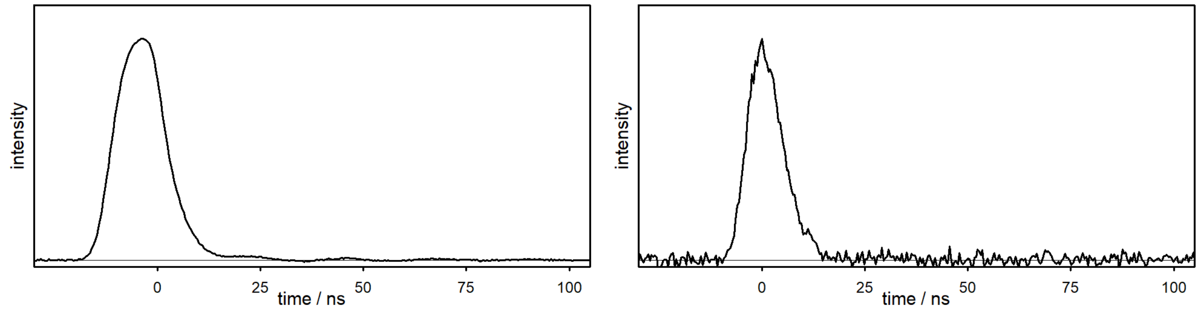

Figure S10.1. Time-resolved emission measurement of **L1'** in acetonitrile (left, OD_{355} = 0.092) and in methanol (right, OD_{355} = 0.103) under inert conditions detected at λ_{det} = 400 nm. Emission lifetimes were not determineable as they are below the detection limit of the setup.

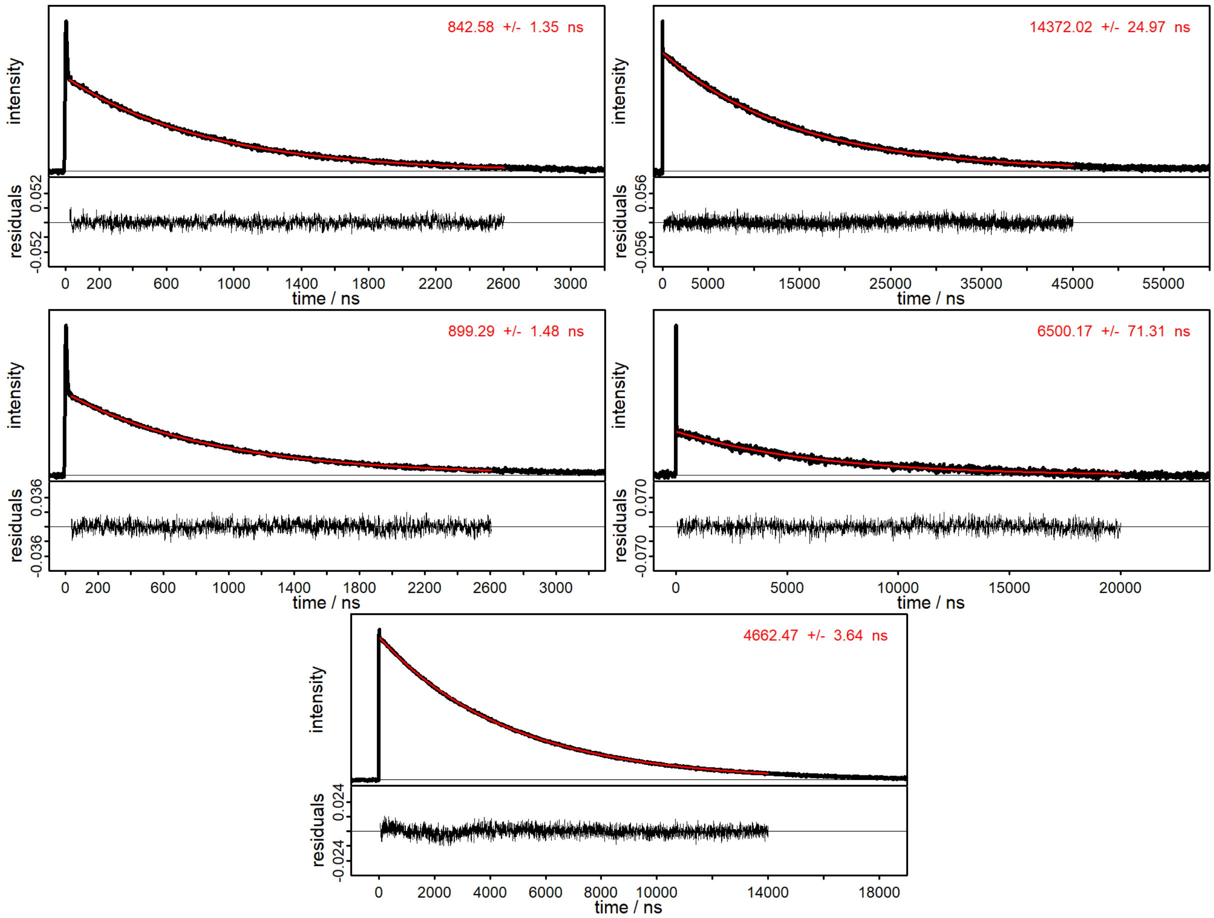

Figure S10.2. Emission decay curves of **C2'** in acetonitrile (top-left, OD_{355} = 0.097) and in methanol (top-right, OD_{355} = 0.095), of **C2** in acetonitrile (middle-left, OD_{355} = 0.107) and in methanol (middle-right, OD_{355} = 0.100) and of [Cu(xant)(2,9-dimethyl-4,7-diphenyl-1,10-phenanthroline)]PF_6 (**CRef**, bottom, OD_{355} = 0.101) in methanol detected at λ_{det} = 570 nm under inert conditions. All lifetimes were estimated using a mono-exponential fit function (shown in red). Residuals of the fit are depicted below.

11 Electrochemical data

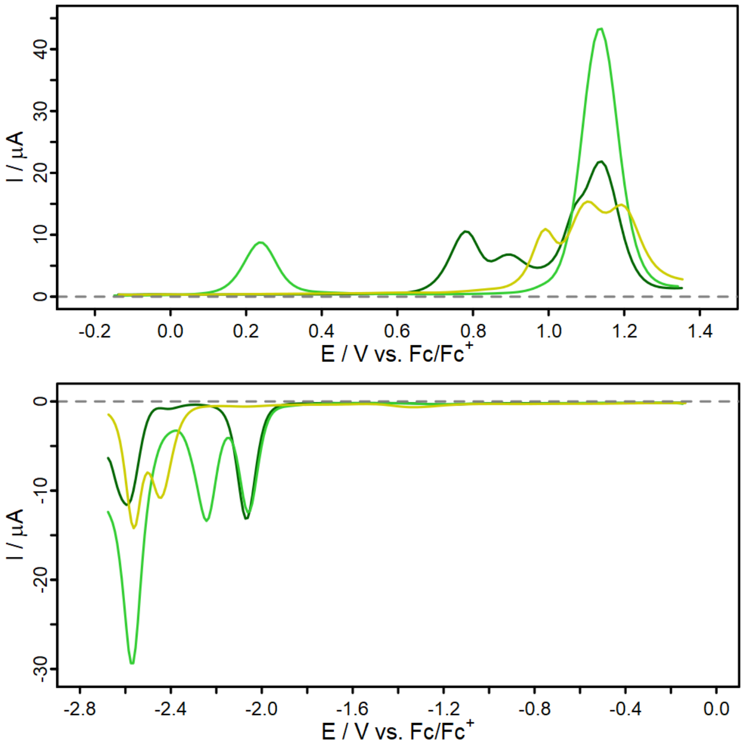

Figure S11.1. Differential pulse voltammograms of **L1'** (yellow), **C1'** (green) and **C2'** (dark green) in deaerated acetonitrile (c = 1 mM).

12 IR spectroscopy

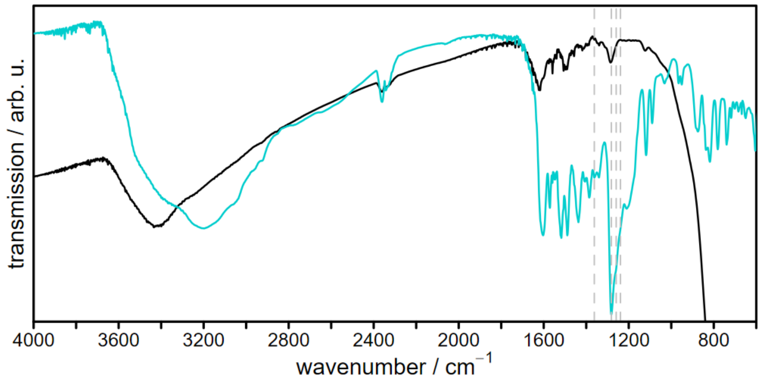

Figure S12.1. IR spectra of the free ligand **L1** (light blue) and the immobilized **L1** on TiO_2 nanoparticles (black). C-OH stretching vibrations (1239 cm^{-1}, 1259 cm^{-1} and 1282 cm^{-1}) and C-OH bending vibration (1362 cm^{-1}) of the catechol groups of the free ligand are denoted by the grey dashed lines.

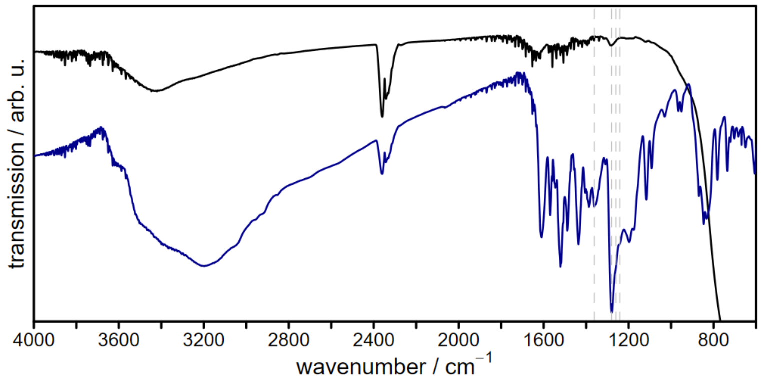

Figure S12.2. IR spectra of the free homoleptic complex **C1** (blue) and the immobilized **C1** on TiO_2 nanoparticles (black). C-OH stretching vibrations (1241 cm^{-1}, 1260 cm^{-1} and 1280 cm^{-1}) and C-OH bending vibration (1362 cm^{-1}) of the catechol groups of the free complex are denoted by the grey dashed lines.

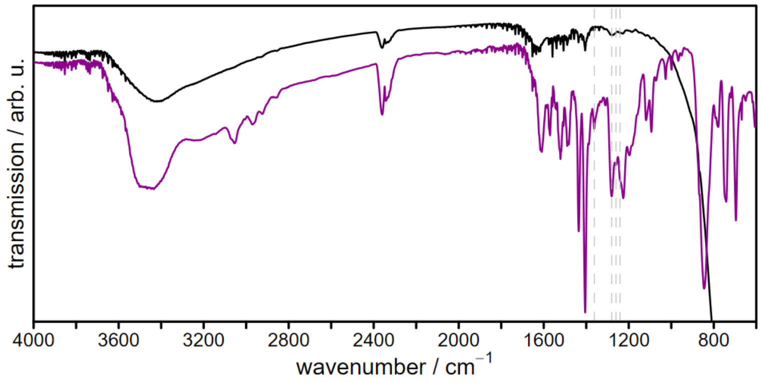

Figure S12.3. IR spectra of the free heteroleptic complex **C2** (dark blue) and the immobilized **C2** on TiO_2 nanoparticles (black). C-OH stretching vibrations (1241 cm^{-1}, 1260 cm^{-1} and 1280 cm^{-1}) and C-OH bending vibration (1362 cm^{-1}) of the catechol groups of the free complex are denoted by the grey dashed lines.

13 Photostability

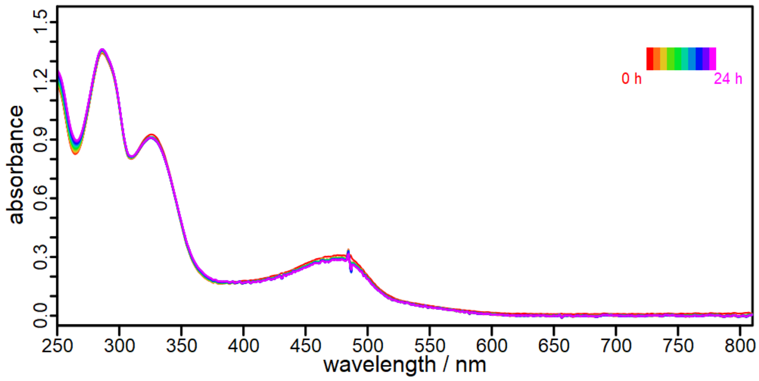

Figure S13.1. UV/vis absorption spectra of **C1'** (c = 0.02 mM) in ambient acetonitrile over a time course of 24 h. The changes in absorbance around 485 nm are inherent to the lamp of the spectrometer used in the experiment.

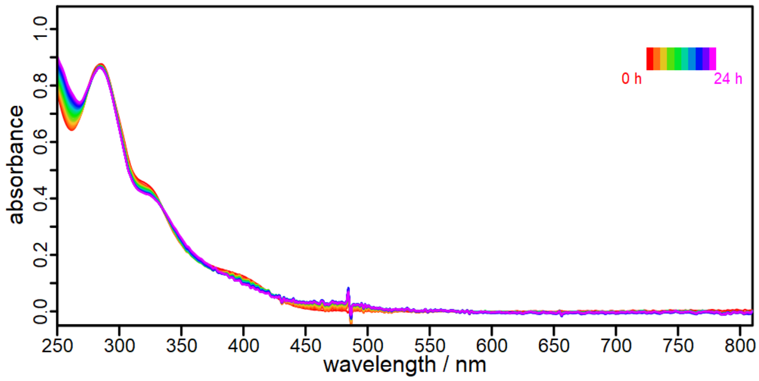

Figure S13.2. UV/vis absorption spectra of **C2'** (c = 0.02 mM) in ambient acetonitrile over a time course of 24 h. The changes in absorbance around 485 nm are inherent to the lamp of the spectrometer used in the experiment.

14 Dye-sensitized solar cells

General information

Table S14.1. Evolution of the figures of merit and overall performances of **L1**-based DSSCs in presence of TBP or GuSCN in the electrolyte.

Sensitizer	Conditions	J_{sc} / mA·cm^{-2}	V_{oc} / mV	FF / %	PCE / %
**L1**	No deviation	3.60 ± 0.24	486.5 ± 5.1	61.3 ± 0.7	1.08 ± 0.08
	0.1 M GuSCN	2.66 ± 0.12	508.7 ± 4.2	64.0 ± 0.7	0.87 ± 0.03
	0.1 M TBP	1.64 ± 0.09	541.5 ± 0.9	65.1 ± 0.5	0.58 ± 0.04
	0.25 TBP	1.26 ± 0.04	561.0 ± 2.1	64.8 ± 0.6	0.46 ± 0.01
	0.5 M TBP	1.17 ± 0.04	574.4 ± 0.2	64.0 ± 0.3	0.43 ± 0.01

Table S14.2. Evolution of the figures of merit and overall performances of **L1**-based DSSCs upon introduction of controlled quantities of CDCA in the dyeing bath.

Sensitizer	Conditions	J_{sc} / mA·cm^{-2}	V_{oc} / mV	FF / %	PCE / %
**L1**	0 mM CDCA	3.85 ± 0.06	501.2 ± 12.5	62.6 ± 0.8	1.21 ± 0.00
	0.5 mM CDCA	3.91 ± 0.13	493.7 ± 1.6	61.7 ± 0.4	1.19 ± 0.05
	5 mM CDCA	3.96 ± 0.17	500.8 ± 2.4	60.6 ± 0.0	1.20 ± 0.06
	10 mM CDCA	4.10 ± 0.02	495 ± 1.5	60.0 ± 0.3	1.22 ± 0 .01
	20 mM CDCA	3.78 ± 0.12	497.2 ± 4.1	61.4 ± 0.5	1.16 ± 0.03
	100 mM CDCA	1.48 ± 0.33	500.9 ± 9.4	48.4 ± 7.4	0.35 ± 0.02

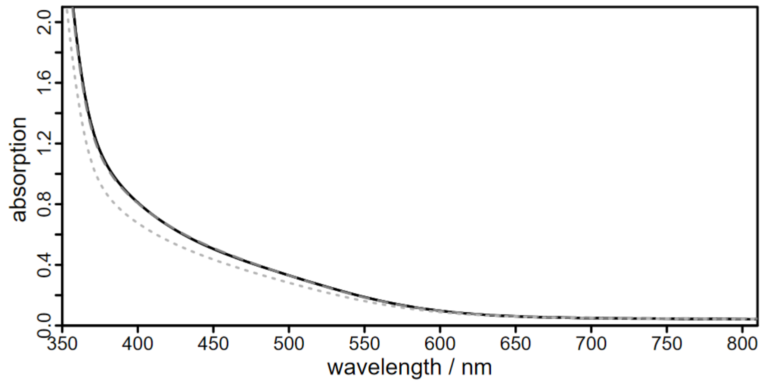

Figure S14.1. UV/vis absorption spectra of **L1** adsorbed on TiO_2 with varying concentrations of CDCA in the dyeing bath (0 mM black, solid line; 0.5 mM dark grey, dashed line; 5 mM CDCA, grey dotted line).

Table S14.3. Evolution of the figures of merit and overall performances of **C2**-based DSSCs in presence of TBP in the electrolyte E2.

Sensitizer	Conditions	J_{sc} / mA·cm^{-2}	V_{oc} / mV	FF / %	PCE / %
**C2**	0 M TBP	4.49 ± 0.35	459.0 ± 32.1	64.5 ± 2.2	1.32 ± 0.05
	0.25 M TBP	4.41 ± 0.28	587.7 ± 1.3	70.0 ± 0.4	1.81 ± 0.10
	0.5 M TBP	4.40 ± 0.10	603.6 ± 1.0	70.8 ± 0.1	1.88 ± 0.05

Table S14.4. Evolution of the figures of merit and overall performances of **C2**-based DSSCs upon introduction of controlled quantities of CDCA in the dyeing bath.

Sensitizer	Conditions	J_{sc} / mA·cm^{-2}	V_{oc} / mV	FF / %	PCE / %
**C2**	0 mM CDCA	3.82 ± 0.18	608.1 ± 5.8	72.8 ± 0.2	1.69 ± 0.09
	0.5 mM CDCA	3.34 ± 0.02	623.9 ± 1.1	73.3 ± 0.5	1.53 ± 0.02
	5 mM CDCA	2.28 ± 0.1	630.5 ± 2.0	72.4 ± 0.2	1.04 ± 0.05

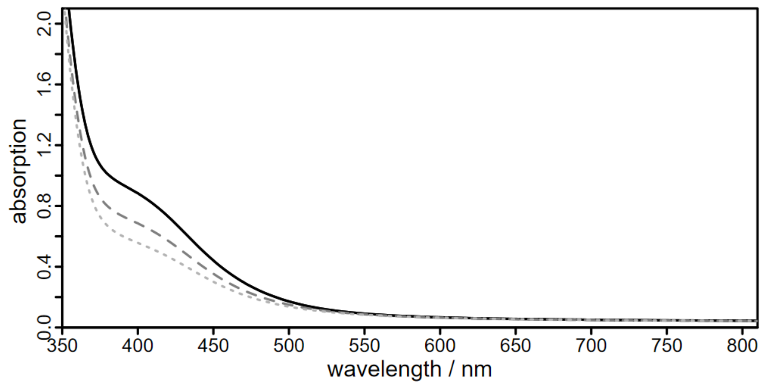

Figure S14.2. UV/vis absorption spectra of **C2** adsorbed on TiO_2 with varying concentrations of CDCA in the dyeing bath (0 mM black, solid line; 0.5 mM dark grey, dashed line; 5 mM CDCA, grey dotted line).

Table S14.5. J_{sc} values obtained from integration of the IPCE spectra.

Sensitizer	Electrolyte	J_{sc} / mA·cm^{-2}
**L1**	E1	4.39
**C1**	E2	1.56
**C2**	E2	5.61

Electrochemical impedance spectroscopy

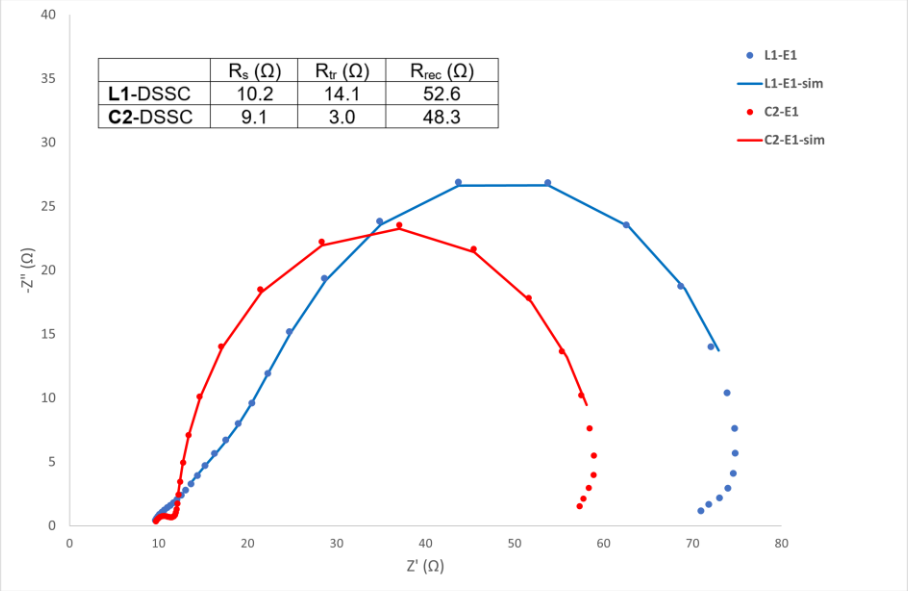

Figure S14.3. EIS−Nyquist plot of DSSCs based on **L1** (blue) and **C2** (red) with electrolyte E1, under AM 1.5 illumination. Dots are experimental data; lines are fits of the experimental data. The first high frequency semi-circle is assigned to the transport resistance (R_{tr}), while the second, mid-frequency semi-circle is assigned to recombination resistance (R_{rec}). For **L1**-based DSSC, the spectrum is significantly distorted owing to strong charge recombination as previously published.[23] Table shows summary of the key parameters extracted from the fittings.

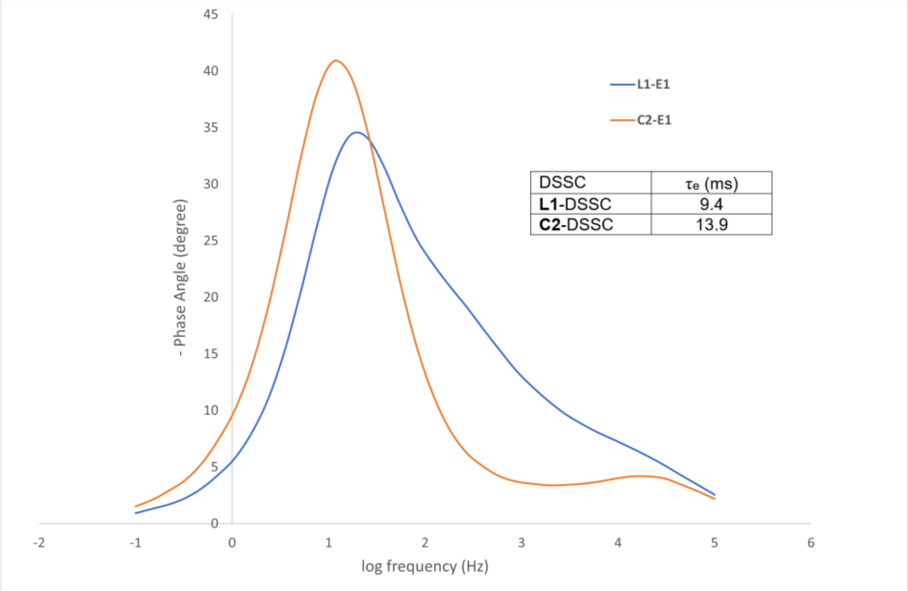

Figure S14.4. Bode phase plot for DSSCs based on **L1** and **C2** with electrolyte E1, under AM 1.5 illumination. τ_e was determined from the maximum peak frequency f_{max} using the formula $\tau_e = 1/(2\pi f_{max})$.[25,26]

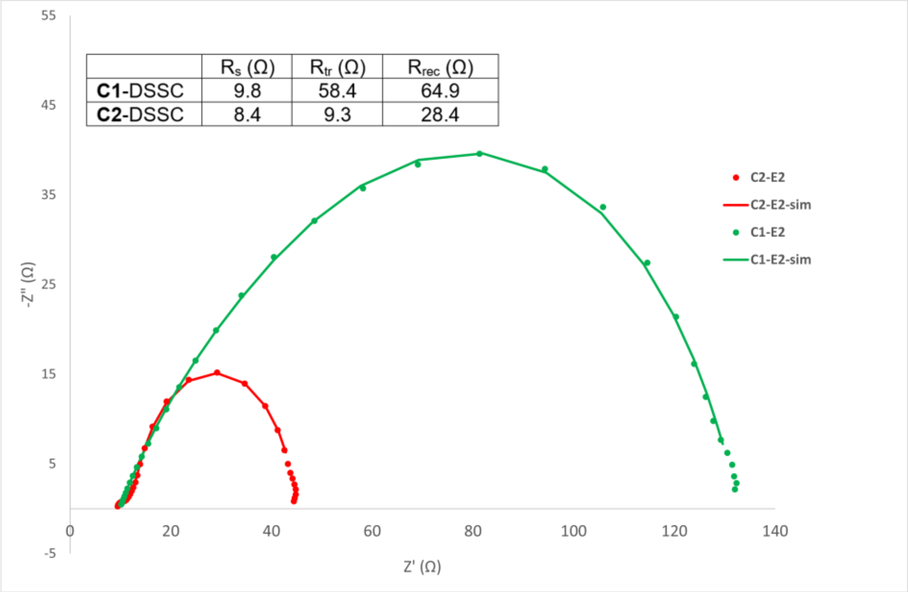

Figure S14.5. EIS−Nyquist plot of DSSCs based on **C1** (green) and **C2** (red) with electrolyte E2, under AM 1.5 illumination. Dots are experimental data; lines are fits of the experimental data. Table shows summary of the key parameters extracted from the fittings.

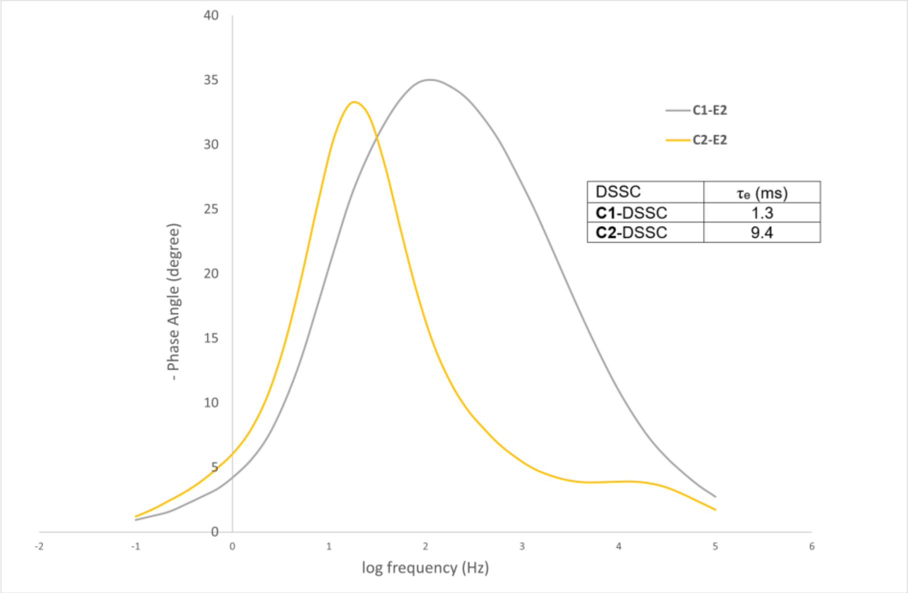

Figure S14.6. Bode phase plot for DSSCs based on **C1** and **C2** with electrolyte E2, under AM 1.5 illumination. τ_e was determined from the maximum peak frequency f_{max} using the formula $\tau_e = 1/(2\pi f_{max})$.[25,26]

Electrochemistry of C1 and C2 on TiO_2

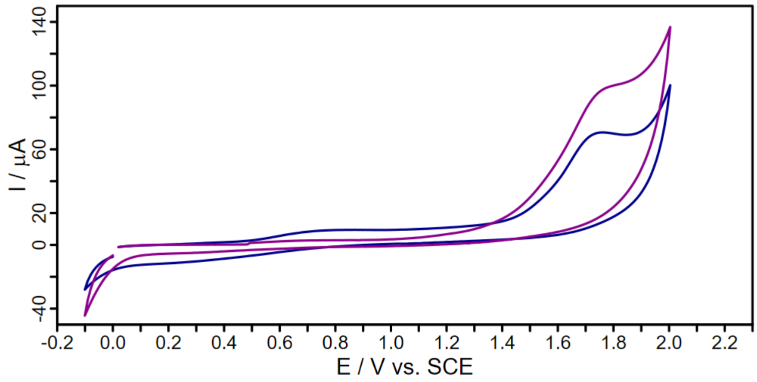

Figure S14.7. Cyclic voltammograms of **C1** (dark blue) and **C2** (magenta) adsorbed on TiO_2 (scan rate = 100mV/s). Working electrode: TiO_2 on FTO substrate as prepared for DSSC; auxiliary electrode: platinum gauze; reference: Ag/AgCl in ethanol. CH_2Cl_2 and 0.1M $TBAPF_6$. The potentials were all calibrated *vs*. SCE by measuring the potential difference between Ag/AgCl and SCE in the same supporting electrolyte.

Energy required to form TiO_2^--Cat^+ charge transfer state was estimated by the onset potential of the oxidation of the TiO_2-Cat chromophore (1.5 V *vs.* SCE for **C1** and **C2**) and the conduction band potential of TiO_2 (-0.7 V vs. SCE) resulting in an energy gap of ≈2.2 eV.

Long-term stability of DSSCs with aqueous electrolytes

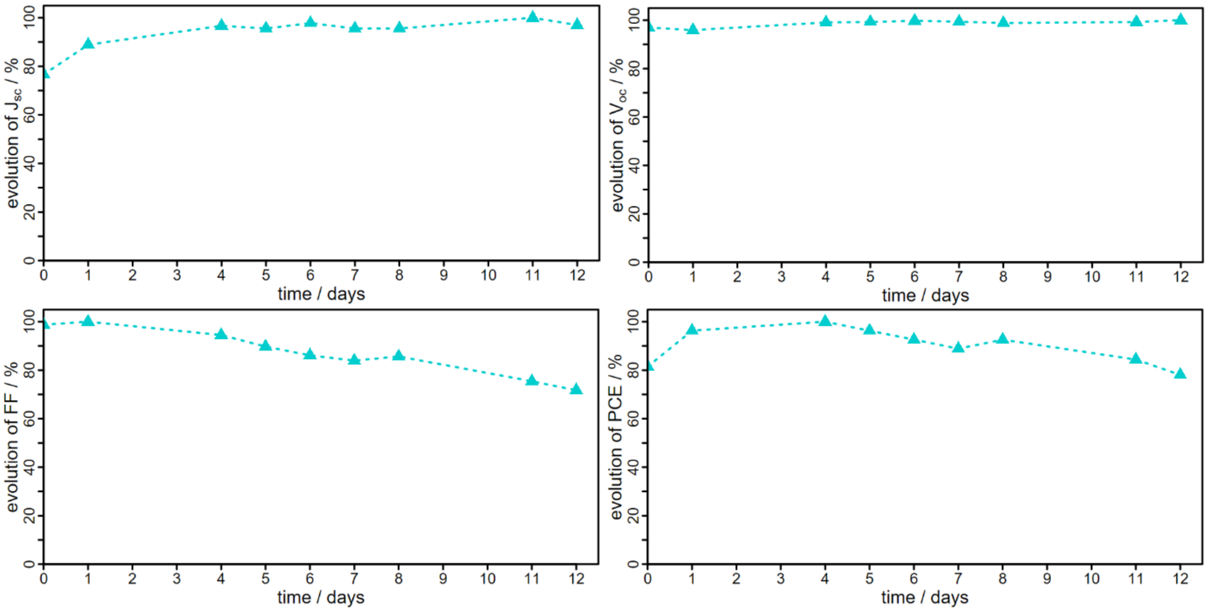

Figure S14.8. Evolution of J_{sc} (top left), V_{oc} (top right) FF (bottom left) and PCE (bottom right) of DSSCs containing **L1** and the aqueous electrolyte E3 over a time course of 12 days.

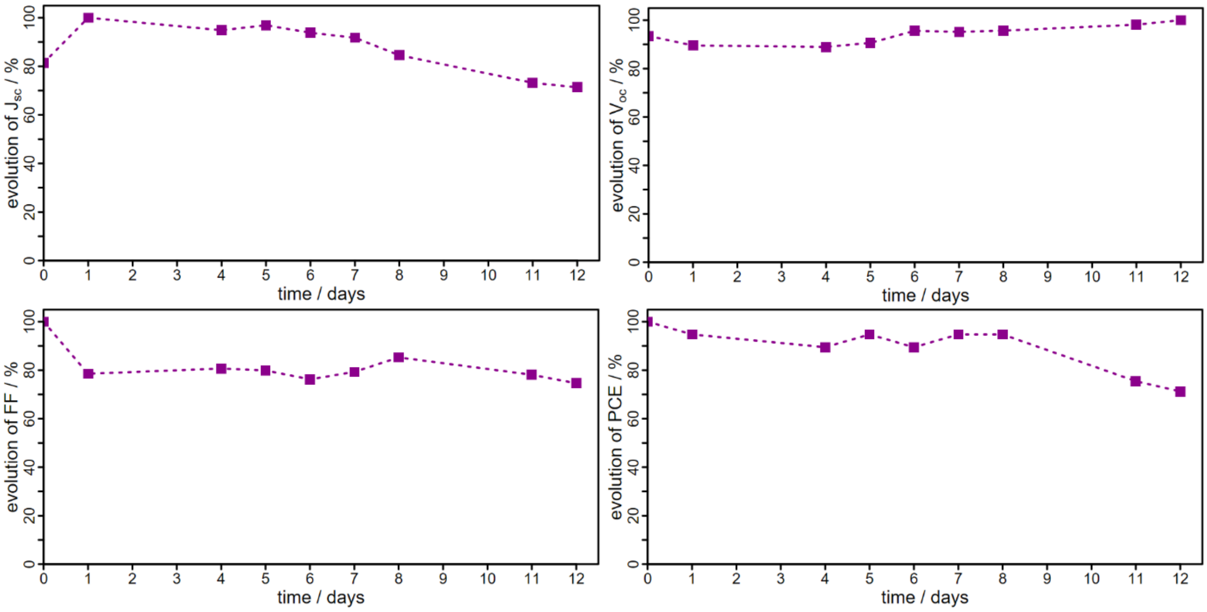

Figure S14.9. Evolution of J_{sc} (top left), V_{oc} (top right) FF (bottom left) and PCE (bottom right) of DSSCs containing **C2** and the aqueous electrolyte E3 over a time course of 12 days.

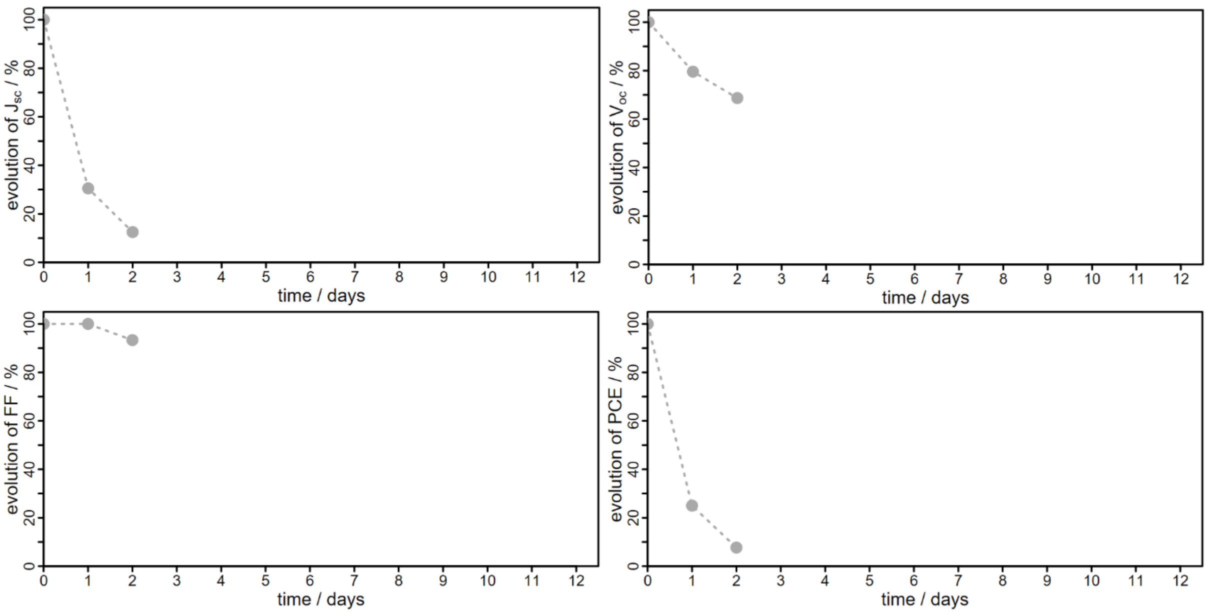

Figure S14.10. Evolution of J_{sc} (top left), V_{oc} (top right) FF (bottom left) and PCE (bottom right) of DSSCs containing **N719** and the aqueous electrolyte E3 over a time course of 12 days.

Previously published heteroleptic Cu(I) complex by Robertson et al.[27]

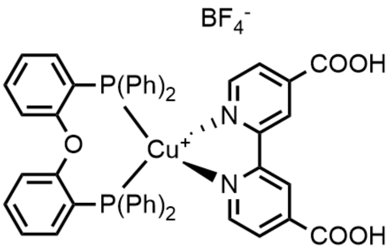

Figure S14.11. Molecular structure of the heteroleptic complex $[Cu(dcbpy)(DPEPhos)]^+$ applied in a DSSC by Roberston and co-workers in 2010.[27]

Table S14.6. Figures of merit and overall performances of the complex $[Cu(dcbpy)(DPEPhos)]^+$ under the conditions with the highest PCE reported by Robertson and co-workers.[27]

Sensitizer	J_{sc} / mA·cm^{-2}	V_{oc} / mV	FF / %	PCE / %
**$[Cu(dcbpy)(DPEPhos)]^+$**	0.233	347	65	0.053

15 References

(1) CrysalisPro, Agilent Technologies, Version 1.171.40.84–1.171.41.122, 2020–2021.
(2) Sheldrick, G. M. A short history of SHELX. *Acta Cryst.* **2008**, *A64*, 112–122.
(3) Sheldrick, G. M. Crystal structure refinement with SHELXL. *Acta Cryst.* **2015**, *C71*, 3–8.
(4) Sheldrick, G. M. SHELXT - Integrated space-group and crystal-structure determination. *Acta Cryst.* **2015**, *A71*, 3–8.
(5) Dolomanov, O. V.; Bourhis, L. J.; Gildea, R. J.; Howard, J. A. K.; Puschmann, H. OLEX2 : a complete structure solution, refinement and analysis program. *J. Appl. Crystallogr.* **2009**, *42*, 339–341.
(6) Diamond – Crystal and Molecular Structure Visualization, Crystal Impact - H. Putz and K. Brandenburg GbR, Bonn, Germany, 2018.
(7) Macrae, C. F.; Sovago, I.; Cottrell, S. J.; Galek, P. T. A.; McCabe, P.; Pidcock, E.; Platings, M.; Shields, G. P.; Stevens, J. S.; Towler, M.*; et al.* Mercury 4.0: from visualization to analysis, design and prediction. *J. Appl. Crystallogr.* **2020**, *53*, 226–235.
(8) Spek, A. L. Structure validation in chemical crystallography. *Acta Cryst.* **2009**, *D65*, 148–155.
(9) Neese, F.; Wennmohs, F.; Becker, U.; Riplinger, C. The ORCA quantum chemistry program package. *J. Chem. Phys.* **2020**, *152*, 224108.
(10) Eichkorn, K.; Treutler, O.; Öhm, H.; Häser, M.; Ahlrichs, R. Auxiliary basis sets to approximate Coulomb potentials. *Chem. Phys. Lett.* **1995**, *240*, 283–290.
(11) Treutler, O.; Ahlrichs, R. Efficient molecular numerical integration schemes. *J. Chem. Phys.* **1995**, *102*, 346–354.
(12) Grimme, S.; Antony, J.; Ehrlich, S.; Krieg, H. A consistent and accurate *ab initio* parametrization of density functional dispersion correction (DFT-D) for the 94 elements H-Pu. *J. Chem. Phys.* **2010**, *132*, 154104.
(13) Grimme, S.; Ehrlich, S.; Goerigk, L. Effect of the Damping Function in Dispersion Corrected Density Functional Theory. *J. Comput. Chem.* **2011**, *32*, 1456–1465.
(14) Weigend, F.; Ahlrichs, R. Balanced basis sets of split valence, triple zeta valence and quadruple zeta valence quality for H to Rn: Design and assessment of accuracy. *Phys. Chem. Chem. Phys.* **2005**, *7*, 3297–3305.
(15) Barone, V.; Cossi, M. Quantum Calculation of Molecular Energies and Energy Gradients in Solution by a Conductor Solvent Model. *J. Phys. Chem. A* **1998**, *102*, 1995–2001.
(16) Chemcraft - graphical software for visualization of quantum chemistry computations. Version 1.8, build 682. https://www.chemcraftprog.com.
(17) Demas, J. N.; Crosby, G. A. The Measurement of Photoluminescence Quantum Yields. A Review. *J. Phys. Chem.* **1971**, *75*, 991-1024.
(18) Karpovich, D. S.; Blanchard, G. J. Relating the Polarity-Dependent Fluorescence Response of Pyrene to Vibronic Coupling. Achieving a Fundamental Understanding of the *py* Polarity Scale. *J. Phys. Chem.* **1995**, *99*, 3951–3958.
(19) Beaudelot, J.; Oger, S.; Peruško, S.; Phan, T.-A.; Teunens, T.; Moucheron, C.; Evano, G. Photoactive Copper Complexes: Properties and Applications. *Chem. Rev.* **2022**, *122*, 16365–16609.
(20) Krivacic, J. R.; Urry, D. W. Ultraviolet and Visible Refractive Indices of Spectro-Quality Solvents. *Anal. Chem.* **1970**, *42*, 596–599.
(21) Grätzel, M. Solar Energy Conversion by Dye-Sensitized Photovoltaic Cells. *Inorg. Chem.* **2005**, *44*, 6841–6851.
(22) Sandroni, M.; Kayanuma, M.; Planchat, A.; Szuwarski, N.; Blart, E.; Pellegrin, Y.; Daniel, C.; Boujtita, M.; Odobel, F. First application of the HETPHEN concept to new heteroleptic bis(diimine) copper(I) complexes as sensitizers in dye sensitized solar cells. *Dalton Trans.* **2013**, *42*, 10818–10827.
(23) Fabregat-Santiago, F.; Garcia-Belmonte, G.; Mora-Seró, I.; Bisquert, J. Characterization of nanostructured hybrid and organic solar cells by impedance spectroscopy. *Phys. Chem. Chem. Phys.* **2011**, *13*, 9083–9118.
(24) Bonnet, S.; Collin, J.-P.; Sauvage, J.-P. Synthesis and Photochemistry of a Two-Position Ru(terpy)(phen)(L)$^{2+}$ Scorpionate Complex. *Inorg. Chem.* **2006**, *45*, 4024–4034.
(25) Asiam, F. K.; Kaliamurthy, A. K.; Rahman, M. M.; Yadagiri, B.; Chen, C.; Kang, H. C.; Sadiq, M.; Ryu, J.; Ewusi Mensah, A.; Zain Qamar, M.*; et al.* Direct charge-transfer mechanism (Type-II) in coordination complexes for sensitization in solar cells: A comprehensive review. *Coord. Chem. Rev.* **2024**, *514*, 215908.
(26) Asiam, F. K.; Rahman, M. M.; Kaliamurthy, A. K.; Muthu, S.; Yadagiri, B.; Kang, H. C.; Chen, C.; Yoo, K.; Lee, J.-J. Role of Pi-Electron Density at the Interface of Small Molecule-Sensitized Solar Cells. *J. Phys. Chem. C* **2023**, *127*, 3928–3939.
(27) Linfoot, C. L.; Richardson, P.; Hewat, T. E.; Moudam, O.; Forde, M. M.; Collins, A.; White, F.; Robertson, N. Substituted [Cu(I)(POP)(bipyridyl)] and related complexes: synthesis, structure, properties and applications to dye-sensitised solar cells. *Dalton Trans.* **2010**, *39*, 8945–8956.
